# Competing risk model with a nonparametric form of relative risks

**DOI:** 10.1007/s10985-026-09728-8

**Published:** 2026-08-19

**Authors:** Youngjin Cho, Pang Du

**Affiliations:** 1 Department of Mathematical Sciences, University of Nevada at Las Vegas, 4505 S. Maryland Pkwy., Las Vegas, NV 89154 USA; 2 Department of Statistics, Virginia Tech, 250 Drillfield Drive, Blacksburg, VA 24061 USA

**Keywords:** Cause-specific hazard, Competing risks, Cox model, Smoothing spline ANOVA, 62N02, 62G05

## Abstract

Competing risks, referring to multiple mutually exclusive cause-specific events in survival data, are commonly seen in medical and engineering studies. In the presence of covariates, the Cox proportional hazards model is the most common approach to modeling covariate effects as the relative risk component for each cause-specific hazard function. However, the linear form of covariate effects in the existing models can be too restrictive to satisfy in practice. In this work, we propose a Cox regression model for competing risk survival data where the relative risk of each cause-specific hazard takes a flexible nonparametric form. The effects of multiple covariates are modeled under a smoothing spline ANOVA framework allowing for nonparametric effect components among covariates. For each cause-specific hazard, we develop an estimation procedure based on the optimization of penalized partial likelihood. We show that our spline estimators of the log relative risk functions can achieved the optimal nonparametric rates of convergence. Through simulation studies, we demonstrate the numerical performance of the proposed method. We then apply our approach to a multiple myeloma dataset, where the effects of gene expressions on cancer and non-cancer related death are investigated. The nonlinear trend revealed in our analysis offers a strong support to the need of a nonparametric modeling approach like ours.

**Supplementary Information:**

The online version contains supplementary material available at https://doi.org/10.1007/s10985-026-09728-8.

## Introduction

In survival analysis, it is common to observe subjects susceptible to multiple, mutually exclusive causes of failure, giving rise to competing risks. When covariates are present, various statistical models have been developed to account for the structure imposed by competing risks. Among these, the cause-specific hazards model based on the Cox proportional hazards framework, or the competing risk Cox model, is one of the most widely adopted approaches and is treated in detail in Prentice et al. (1978) and Chapter 8 of Kalbfleisch and Prentice (2002). Several alternative or extended approaches of this method have since been proposed. For example, Fine and Gray (1999) proposed a proportional subdistribution hazards model to directly assess the effect of each risk factor on the cumulative incidence function. Lunn and Mcneil (1995) proposed a data augmentation method to model multiple cause-specific hazards simultaneously using standard Cox regression. Modeling of correlated latent cause-specific event times using a copula is presented in Lo and Wilke (2010). For a comprehensive review of methodological developments in competing risks analysis, see Monterrubio-Gómez et al. (2024).

Meanwhile, nonparametric smooth modeling in survival analysis becomes essential when parametric assumptions become doubtful in practice. For example, kernel-based survival methods were considered in López-Cheda et al. (2017, 2023) for cure rate data and Akritas and Keilegom (2003) for bivariate survival data. Penalty-based regression spline methods were studied in Joly et al. (1998), Joly et al. (2002) and Commenges et al. (2014) for single event survival data with various censoring/truncation schemes, Rondeau et al. (2003) for correlated survival data, and Joly and Commenges (1999) for three-state data. Among nonparametric methods, smoothing spline survival models (Gu 2013) provide an especially appealing approach due to its rigorous mathematical foundation in the reproducing kernel Hilbert space framework, straightforward extension to the multi-dimensional setting through tensor product spaces and the functional ANOVA structure, and reliable performance even in estimating multivariate functions. Besides the single event survival models (Gu 2013), smoothing splines based methods have been studied in various other survival settings, such as high dimensional survival data (Du et al. 2010), recurrent event data (Du et al. 2011), and cure rate data (Wang et al. 2012).

Despite the rich literature in their respective areas, an integration of nonparametric smoothing into competing risk models have been rarely studied, with a few exceptions described below. For discrete-time competing risks survival data, Luo et al. (2016) proposed a Cox–logistic regression model of the cause-specific hazards, where only the baseline hazards are estimated nonparametrically by simple quadratic or cubic spline expansions but the covariate effects are modeled linearly. Fermanian (2003) proposed a joint distribution approach based on kernel methods. In particular, the joint survival function of the competing failure times is assumed to have a form defined by three components, an unknown cumulative distribution function, a set of cause-specific time functions, and a set of cause-specific covariate functions. Various constraints on the component functions, such as boundary conditions, monotonicity, and differenitability are necessary to make the model identifiable. Under these constraints, these three components are estimated separately by kernel methods. Despite its nonparametric nature, this method can be cumbersome to implement and its estimates are hard to interpret in practice due to its complex structure.

In this paper, we consider a nonparametric competing risk model based on the smoothing spline ANOVA framework. Instead of linear forms of covariate effects adopted in most parametric or semiparametric competing risk models, we consider cause-specific hazards with the true covariate effect functions assumed to lie in a tensor-product Sobolev space. This nonparametric formulation allows for flexible inclusion and decomposition of covariate effects. The estimation is through the optimization of a penalized partial likelihood consisting of the negative log partial likelihood representing the goodness-of-fit, a roughness penalty enforcing the smoothness constraint, and a smoothing parameter balancing the tradeoff. For the inference purpose, we consider the point-wise confidence intervals based on Baysian interpretation of the penalized partial likelihood. A smoothing parameter selection criterion based on the Kullback–Leibler distance is introduced. For theoretical properties, we establish the rates of convergence for the function parameters in the proposed model. The model is evaluated through simulations, and then applied to a multiple myeloma dataset studying the effect of genes on cause-specific hazards. The analysis reveals nonlinear and distinct effects across risk factors, demonstrating the advantage of the proposed model over existing methods that often neglect competing risks or rely on linear covariate effects. As one of the first nonparametric competing risk models, our method has the following advantages: (1) flexible model assumptions via the functional ANOVA decomposition without linearity restriction; (2) theoretically consistent estimation with the optimal convergence rate for nonparametric functions; (3) numerical stability with a convex objective for optimization; (4) efficient computation with a manageable dimension of parameter space; and (5) straightforward practical interpretation via the functional ANOVA structure and proper confidence interval estimates.

For the remainder of the paper, Sect. 2 presents the model formulation, function space structure, estimation procedure, and theoretical result. Section 3 provides simulation studies for the proposed model. Section 4 presents a real data application. Finally, Sect. 5 concludes the paper with a discussion of future research directions.

## Smoothing spline competing risk cox model

### Model structure

Let $\tilde{T} \in (0, \infty )$ and $C \in (0, \tau ]$ be respectively the underlying event time and non-informative right-censoring time, with $\tau \in (0,\infty )$ representing the ending time of the study. Let $X = (X_{[1]}, \ldots , X_{[d]})^{\top } \in \mathcal {X}= \prod _{j=1}^d \mathcal {X}_{[j]}$ be a vector of *d* covariates. For a categorical covariate, $\mathcal {X}_{[j]}$ is a finite set $\{1,\ldots ,Q_j\}$. For a continuous covariate, we set $\mathcal {X}_{[j]}=[0,1]$ without loss of generality. Suppose there are *K* mutually exclusive risks, denoted by $\{1, \ldots , K\}$, that can be the cause of an event. Let $\tilde{G} = \sum _{k=1}^K k 1\!\!1(\tilde{T} \text { is due to the } k\text {-th risk}) \in \{1, \ldots , K\}$ represent the underlying risk associated with the event. We assume that a single subject can experience at most one event, and that $\tilde{T}$ and *C* are conditionally independent given *X*. Let $T = \min \{\tilde{T}, C\} \in (0, \tau ]$ be the observed time and $\mathscr {I} = 1\!\!1(\tilde{T} \le C)$ be the censoring indicator. Let $G = \tilde{G} \cdot \mathscr {I} \in \{0,1,\ldots ,K\}$ be the observed risk indicator. Note that *G* is 0 when the subject is censored and is the risk type otherwise.

Let $\{W_i\}_{i=1}^n = \{\tilde{T}_i,C_i,X_i,\tilde{G}_i\}_{i=1}^n$ be the underlying data, which are *n* independent and identically distributed copies from $W = \{\tilde{T},C,X,\tilde{G}\}$. The observations $\{T_i,X_i,\mathscr {I}_i,G_i\}_{i=1}^n$ are then copies from $\{T,X,\mathscr {I},G\}$. In competing risk models, for $t \in [0, \infty )$, the hazard function of $\tilde{T}$ given * X * is

$$\begin{aligned} dH(t;X)&=h(t;X)dt=\mathbb {P}(\tilde{T} \in [t, t+dt)|\tilde{T}\ge t, X)\\&=\sum _{k=1}^K \mathbb {P}(\tilde{T} \in [t, t+dt), \tilde{G}=k|\tilde{T}\ge t, X)=\sum _{k=1}^K dH_k(t;X) = \sum _{k=1}^K h_k(t;X)dt, \end{aligned}$$

where $dH_k(t;X)$ is the cause-specific hazard function of the *k*-th risk factor. The survival function is given by $\textsf{S}(t;X)=\mathbb {P}(\tilde{T} > t | X)=\exp (-H(t;X))$ and the distribution function is then calculated as $\textsf{F}(t;X)=\mathbb {P}(\tilde{T} \le t | X)=1-\textsf{S}(t;X)$. The density function is $\textsf{f}(t;X)={\partial } \textsf{F}(t;X)/({\partial t})=h(t;X)\textsf{S}(t;X)=\sum _{k=1}^K \textsf{f}_k(t;X)$, where $\textsf{f}_k(t;X)=h_k(t;X)\textsf{S}(t;X)$. The distribution function can also be expressed as $\textsf{F}(t;X)=\sum _{k=1}^K \textsf{F}_k(t;X)$, where $\textsf{F}_k(t;X)=\mathbb {P}(\tilde{T} \le t, \tilde{G}=k | X)=\int _0^t \textsf{f}_k(s;X) ds$ is the cumulative incidence function of the *k*-th risk factor. Define $\textsf{P}(k;X)=\mathbb {P}(\tilde{G}=k|X)=\lim _{t\rightarrow \infty } \textsf{F}_k(t;X)$, where $\sum _{k=1}^K \textsf{P}(k;X)=1$.

For each *k*, let $\eta _k^*: \mathcal {X} \rightarrow \mathbb {R}$ denote the true covariate effect function for the *k*-th risk. We assume the proportional cause-specific hazards model, that is, the cause-specific hazard functions follow the Cox model

$$\begin{aligned} dH_k(t;X)=dH_{0k}(t)\exp (\eta _k^*(X)), \end{aligned}$$

where $dH_{0k}(t)=h_{0k}(t)dt$ is the baseline cause-specific hazard function, and $h_{0k}(0)=0$.

### Reproducing Kernel Hilbert spaces

The unknown covariate effect function $\eta _k^*$ is assumed to belong to a reproducing kernel Hilbert space (RKHS) $\mathcal {H}$ of multivariate functions defined on the product domain of the covariates. For each covariate, the marginal space is constructed first according to the covariate type (e.g., continuous or categorical). Then these marginal spaces for all the covariates are assembled to form $\mathcal {H}$ through a tensor product of spaces. This construction admits a decomposition of $\mathcal {H}$ into effect subspaces corresponding to main and interaction effects, analogous to the classical ANOVA decomposition. The functional ANOVA represents $\eta _k^*$ as a sum of interpretable components, enabling separate estimation and inference for each effect.

For the *j*th covariate, let $\mathcal {H}_{[j]}$ be the RKHS of functions defined on $\mathcal {X}_{[j]}$. For a discrete covariate, $\mathcal {X}_{[j]} = \{1,\ldots ,Q_j\}$ and it is easy to see that $\mathcal {H}_{[j]} = \mathbb {R}^{Q_j}$ (Gu 2013), where the covariate effect is represented by $Q_j$ level-specific parameters. For a continuous covariate, $\mathcal {X}_{[j]} = [0,1]$ and $\mathcal {H}_{[j]}$ is the Sobolev space of order $m \in \mathbb {N}$, defined as

$$\begin{aligned} & \mathcal {H}_{[j]} = \Bigg \{ f: f, f^{(1)}, \ldots , f^{(m-1)} \ \text {are absolutely continuous},\nonumber \\ & \quad \text { and } \int _{\mathcal {X}_{[j]}} |f^{(m)}(x_{[j]})|^2 \, dx_{[j]} < \infty \Bigg \}.\end{aligned}$$

The marginal space $\mathcal {H}_{[j]}$ can be decomposed into a constant subspace and a centered effect subspace through an averaging operator. The idea is similar to the zero sum constraint in the classical ANOVA where the average of all the treatment effects is assumed to be zero. Let $\mathcal {A}_{[j]}$ be the averaging operator for $\mathcal {H}_{[j]}$. When $\mathcal {X}_{[j]} = \{1,\ldots ,Q_j\}$, $\mathcal {A}_{[j]}f(x)=$ $\mathcal {A}_{[j]}f(x_{[1]},\ldots ,x_{[d]})=\sum _{x_{[j]}=1}^{Q_j}f(x_{[1]},\ldots ,x_{[d]})/Q_j.$ When $\mathcal {X}_{[j]} = [0,1]$, $\mathcal {A}_{[j]}f(x)=\mathcal {A}_{[j]}f(x_{[1]},\ldots ,x_{[d]})=\int _{\mathcal {X}_{[j]}}f(x_{[1]},\ldots ,x_{[d]})dx_{[j]}$. Then $\mathcal {H}_{[j]}$ can be decomposed as $\mathcal {H}_{[j]} = \mathcal {H}_{\emptyset [j]} \oplus \mathcal {H}_{\{j\}},$ where

$$\mathcal {H}_{\emptyset [j]} = \left\{ c_j \mathcal {A}_{[j]} f_{[j]} : c_j \in \mathbb {R}, f_{[j]} \in \mathcal {H}_{[j]} \right\} = \text {Span}\{1\},$$

and $\mathcal {H}_{\{j\}} = \{ f_{[j]} \in \mathcal {H}_{[j]}: \mathcal {A}_{[j]} f_{[j]} = 0 \}$. Thus, each marginal space $\mathcal {H}_{[j]}$ is decomposed into a constant component $\mathcal {H}_{\emptyset [j]}$ and a centered effect component $\mathcal {H}_{\{j\}}$.

The tensor product Sobolev space on $\mathcal {X}= \prod _{j=1}^d \mathcal {X}_{[j]}$ can be written as

$$\begin{aligned} \otimes _{j=1}^d \mathcal {H}_{[j]}&=\otimes _{j=1}^d \left\{ \mathcal {H}_{\emptyset [j]} \oplus \mathcal {H}_{\{j\}}\right\} \\&=\oplus _{S \in \mathcal {P}_d} \mathcal {H}_S = \mathcal {H}_\emptyset \oplus \left\{ \oplus _{j=1}^d \mathcal {H}_{\{j\}} \right\} \oplus \left\{ \oplus _{j<j'} \mathcal {H}_{\{j,j'\}} \right\} \oplus \ldots \oplus \mathcal {H}_{\{1,\ldots ,d\}}, \end{aligned}$$

where $\mathcal {P}_d$ denotes the power set of $\{1,\ldots ,d\}$ and the components in the decomposition respectively represent the constant, the main effects, the two-way interactions, and so on. For each $S \in \mathcal {P}_d$, the associated effect space is

$$ \mathcal {H}_S=\{\otimes _{j \in S}\mathcal {H}_{\{j\}}\} \otimes \{\otimes _{j \in \{1,\ldots ,d\} {\setminus } S} \mathcal {H}_{\emptyset [j]}\}=\{\otimes _{j \in S} \mathcal {H}_{\{j\}}\} \otimes \text {Span}\{1\}=\otimes _{j \in S} \mathcal {H}_{\{j\}}. $$

For example, $\mathcal {H}_\emptyset =\otimes _{j=1}^d \mathcal {H}_{\emptyset [j]}=\text {Span}\{1\}$ is the constant space, $\mathcal {H}_{\{j\}}$ corresponds to the main effect of covariate *j*, while $\mathcal {H}_{\{j,j'\}}$ corresponds to the interaction effect between covariates *j* and *j'*.

In practice, not all effect spaces need to be included in the model. Let $\mathcal {H} = \oplus _{S \in \mathbb {S}} \mathcal {H}_S$ for some nonempty $\mathbb {S} \subseteq \mathcal {P}_d$. Note that, since the Cox model does not include an intercept, $\mathcal {H}_\emptyset$ is excluded from $\mathcal {H}$. A further selection of $\mathbb {S}$ specifies the structure of $\mathcal {H}$. For instance, if $\mathbb {S}=\left\{ \{1\},\ldots ,\{d\}\right\}$, then $\mathcal {H}$ adopts an additive form that involves only main effects. On the other hand, choosing $\mathbb {S}=\left\{ \{1\},\ldots ,\{d\},\{1,2\},\ldots ,\{d-1,d\}\right\}$ allows $\mathcal {H}$ to incorporate all the main effects and two-way interactions. For each risk *k*, we assume $\eta ^*_k \in \mathcal {H}$, and let its decomposition be $\eta ^*_k = \sum _{S\in \mathbb {S}} \eta ^*_{k,S}$ where $\eta ^*_{k,S} \in \mathcal {H}_S$ for every $S \in \mathbb {S}$.

Next, we introduce the roughness penalty induced by $\mathcal {H}$. Given a semi-inner product $J(\cdot ,\cdot )$ on $\mathcal {H}$, $\mathcal {H}$ can be decomposed as $\mathcal {H} = \mathcal {N}_J \oplus \mathcal {H}_J$, where the null space $\mathcal {N}_J = \{ f \in \mathcal {H} : J(f) = J(f,f) = 0 \}$ forms a finite-dimensional subspace of $\mathcal {H}$, and $\mathcal {H}_J$ forms an infinite-dimensional subspace of $\mathcal {H}$. Furthermore, $J(\cdot ,\cdot )$ is an inner product on $\mathcal {H}_J$, whose associated reproducing kernel (RK) is $\mathcal {K}_J(\cdot ,\cdot )$. For example, when *d=1* and $\mathcal {H}$ becomes a space of univariate functions, the squared-curvature penalty $J(f)=\int [f''(x)]^2 dx$ yields cubic smoothing splines. To distinguish the spaces used for different competing risks, we use $J_k$ and $\mathcal {K}_{J_k}$ to represent the roughness penalty and its associated reproducing kernel for the estimation of $\eta ^*_k$.

#### Example 1

Consider *d=2*, $\mathcal {X}_{[1]}=\mathcal {X}_{[2]}=[0,1]$, $\mathbb {S}=\{\{1\},\{2\},\{1,2\}\}$, and *m=2*. This corresponds to the scenario of two continuous covariates, a model with all the main and interaction effects, and a marginal squared-curvature penalty.

Let $\kappa _{l}(x)$ be the *l*-th scaled Bernoulli polynomial on [0, 1]. For instance, $\kappa _{1}(x) = x - 0.5$, $\kappa _{2}(x) = (\kappa _{1}^2(x) - 1/12)/2$, and $\kappa _{4}(x) = (\kappa _{1}^4(x) - \kappa _{1}^2(x)/2 + 7/240)/24$. The null space $\mathcal {N}_J=\text {Span}\{ \kappa _{1}(x_{[1]}), \kappa _{1}(x_{[2]}), \kappa _{1}(x_{[1]})\kappa _{1}(x_{[2]}) \}$, consisting of low-order polynomial components that are not penalized by $J_k$. For $x=(x_{[1]},x_{[2]})$ and $x'=(x'_{[1]},x'_{[2]})$ in $\mathcal {X}_{[1]}\times \mathcal {X}_{[2]}$, and $f,g\in \mathcal {H}$,

$$\begin{aligned} \mathcal {K}_{J_k}(x,x')&=\theta _{k,1}\left\{ \kappa _{2}(x_{[1]})\kappa _{2}(x_{[1]}') - \kappa _{4}(|x_{[1]}-x_{[1]}'|) \right\} \\&+\theta _{k,2}\left\{ \kappa _{2}(x_{[2]})\kappa _{2}(x_{[2]}') - \kappa _{4}(|x_{[2]}-x_{[2]}'|) \right\} \\&+\theta _{k,3}\left\{ \kappa _{2}(x_{[1]})\kappa _{2}(x_{[1]}') - \kappa _{4}(|x_{[1]}-x_{[1]}'|) \right\} \kappa _{1}(x_{[2]})\kappa _{1}(x_{[2]}')\\&+\theta _{k,4}\left\{ \kappa _{2}(x_{[2]})\kappa _{2}(x_{[2]}') - \kappa _{4}(|x_{[2]}-x_{[2]}'|) \right\} \kappa _{1}(x_{[1]})\kappa _{1}(x_{[1]}')\\&+\theta _{k,5}\left\{ \kappa _{2}(x_{[1]})\kappa _{2}(x_{[1]}') - \kappa _{4}(|x_{[1]}-x_{[1]}'|) \right\} \\&\times \left\{ \kappa _{2}(x_{[2]})\kappa _{2}(x_{[2]}') - \kappa _{4}(|x_{[2]}-x_{[2]}'|) \right\} ,\\ J_k(f,g)&=\frac{1}{\theta _{k,1}}\int _{\mathcal {X}_{[1]}}\{ \int _{\mathcal {X}_{[2]}}f^{(2)}_{\langle 11\rangle }(x)dx_{[2]}\int _{\mathcal {X}_{[2]}}g^{(2)}_{\langle 11\rangle }(x)dx_{[2]}\} dx_{[1]}\\&+\frac{1}{\theta _{k,2}}\int _{\mathcal {X}_{[2]}}\{ \int _{\mathcal {X}_{[1]}}f^{(2)}_{\langle 22\rangle }(x)dx_{[1]}\int _{\mathcal {X}_{[1]}}g^{(2)}_{\langle 22\rangle }(x)dx_{[1]}\} dx_{[2]}\\&+\frac{1}{\theta _{k,3}}\int _{\mathcal {X}_{[1]}}\{ \int _{\mathcal {X}_{[2]}}f^{(3)}_{\langle 112\rangle }(x)dx_{[2]}\int _{\mathcal {X}_{[2]}}g^{(3)}_{\langle 112\rangle }(x)dx_{[2]}\} dx_{[1]}\\&+\frac{1}{\theta _{k,4}}\int _{\mathcal {X}_{[2]}}\{ \int _{\mathcal {X}_{[1]}}f^{(3)}_{\langle 221\rangle }(x)dx_{[1]}\int _{\mathcal {X}_{[1]}}g^{(3)}_{\langle 221\rangle }(x)dx_{[1]}\} dx_{[2]}\\&+\frac{1}{\theta _{k,5}}\int _{\mathcal {X}}f^{(4)}_{\langle 1122\rangle }(x)g^{(4)}_{\langle 1122\rangle }(x) dx, \end{aligned}$$

where ${f}^{(1)}_{\langle 2\rangle }(x)=\partial f(x)/(\partial x_{[2]})$, ${f}^{(2)}_{\langle 11\rangle }(x)=\partial ^2 f(x)/(\partial x_{[1]}^2)$, and so on for the higher order derivatives. Here, $\theta _{k,v} \in (0,\infty )$ are weighting parameters for subspaces which controls the relative amount of smoothing imposed on different subspace components.

#### Example 2

When $\mathcal {X}_{[1]}=\{1,\ldots ,Q_1\}$ and $\mathcal {X}_{[2]}=[0,1]$, we have one categorical covariate and one continuous covariate, and $\mathcal {N}_J=\text {Span}\{ \kappa _{1}(x_{[2]}) \}$. For $x=(x_{[1]},x_{[2]})$ and $x'=(x'_{[1]},x'_{[2]})$ in $\mathcal {X}_{[1]}\times \mathcal {X}_{[2]}$, and $f,g\in \mathcal {H}$,

$$\begin{aligned} \mathcal {K}_{J_k}(x,x')&=\theta _{k,1}\left\{ \kappa _{2}(x_{[2]})\kappa _{2}(x_{[2]}') - \kappa _{4}(|x_{[2]}-x_{[2]}'|) \right\} /Q_1\\&+\theta _{k,2}\left\{ 1\!\!1(x_{[1]}=x_{[1]}')-1/Q_1 \right\} \\&+\theta _{k,3}\left\{ 1\!\!1(x_{[1]}=x_{[1]}')-1/Q_1 \right\} \kappa _{1}(x_{[2]})\kappa _{1}(x_{[2]}')\\&+\theta _{k,4}\left\{ 1\!\!1(x_{[1]}=x_{[1]}')-1/Q_1 \right\} \left\{ \kappa _{2}(x_{[2]})\kappa _{2}(x_{[2]}') - \kappa _{4}(|x_{[2]}-x_{[2]}'|) \right\} ,\\ J_k(f,g)&=\frac{1}{\theta _{k,1}}\int _{\mathcal {X}_{[2]}}\left\{ \sum _{x_{[1]}=1}^{Q_1} f^{(2)}_{\langle 22\rangle }(x) \right\} \left\{ \sum _{x_{[1]}=1}^{Q_1} g^{(2)}_{\langle 22\rangle }(x) \right\} dx_{[2]} /Q_1\\&+\frac{1}{\theta _{k,2}} \sum _{x_{[1]}=1}^{Q_1} \left\{ \int _{\mathcal {X}_{[2]}}(id-\mathcal {A}_{[1]})f(x) dx_{[2]} \right\} \left\{ \int _{\mathcal {X}_{[2]}}(id-\mathcal {A}_{[1]})g(x) dx_{[2]} \right\} \\&+\frac{1}{\theta _{k,3}} \sum _{x_{[1]}=1}^{Q_1} \left\{ \int _{\mathcal {X}_{[2]}}(id-\mathcal {A}_{[1]})f^{(1)}_{\langle 2\rangle }(x) dx_{[2]} \right\} \left\{ \int _{\mathcal {X}_{[2]}}(id-\mathcal {A}_{[1]})g^{(1)}_{\langle 2\rangle }(x) dx_{[2]} \right\} \\&+\frac{1}{\theta _{k,4}} \int _{\mathcal {X}_{[2]}} \left\{ \sum _{x_{[1]}=1}^{Q_1} (id-\mathcal {A}_{[1]})f^{(2)}_{\langle 22\rangle }(x) (id-\mathcal {A}_{[1]})g^{(2)}_{\langle 22\rangle }(x) \right\} dx_{[2]}, \end{aligned}$$

where *id* is the identity mapping on $\mathcal {X}_{[1]}\times \mathcal {X}_{[2]}$.

### Martingale structure

Here, we derive the martingale structure for our model, based on Fleming and Harrington (1991) and Kalbfleisch and Prentice (2002). Let $t \in [0, \tau ]$. For each risk *k*, the counting process is defined as $N_{k}(t)=1\!\!1(T \le t, G =k, \mathscr {I}=1)=1\!\!1(\tilde{T} \le t, \tilde{T} \le C, \tilde{G}=k),$ and the at-risk process is defined as $Y(t)=1\!\!1(T \ge t)=1\!\!1(\tilde{T} \ge t, C \ge t).$ For each subject *i*, $N_{ik}(\cdot )$ and $Y_i(\cdot )$ are defined in the same manner.

The filtration at * t * and $t^-$ are each defined as

$$\begin{aligned}&\mathcal {F}_t=\sigma \left( \left\{ \{N_{ik}(s), Y_i(s^+), X_i\}_{i=1}^n ; k \in \{1,\ldots ,K\} ; 0 \le s \le t \right\} \right) , \\&\mathcal {F}_{t^-}=\sigma \left( \left\{ \{N_{ik}(s), Y_i(s), X_i\}_{i=1}^n ; k \in \{1,\ldots ,K\} ; 0 \le s < t \right\} \right) , \end{aligned}$$

where the *σ *-algebra generated by *· * is denoted by $\sigma (\cdot )$.

As $\{\tilde{T}_i\}_{i=1}^n$ and $\{C_i\}_{i=1}^n$ are conditionally independent given $\{X_i\}_{i=1}^n$, we have

$$\begin{aligned} \mathbb {E}(dN_{ik}(t)|\mathcal {F}_{t^-})=\mathbb {P}(dN_{ik}(t)=1|\mathcal {F}_{t^-})=Y_i(t)dH_k(t;X_i), \end{aligned}$$

where $dN_{ik}(t)=N_{ik}(t^-+dt)-N_{ik}(t^-)$. By the Doob-Meyer decomposition, we have $N_{ik}(t)=A_{ik}(t)+M_{ik}(t),$ where $M_{ik}(t)$ is a zero-mean martingale and $A_{ik}(t)=\int _0^t dA_{ik}(s)=\int _{0}^t Y_i(s)dH_k(s;X_i)$ is the compensator. The predictable variation process and covariation process are calculated respectively as $\langle M_{ik} \rangle (t)=\int _0^t d \langle M_{ik} \rangle (s)$ and $\langle M_{ik}, M_{i'k} \rangle (t)=\int _0^t d \langle M_{ik}, M_{i'k} \rangle (s)$, where

$$\begin{aligned} d\langle M_{ik} \rangle (t)&\equiv \text {Var}(dM_{ik}(t)|\mathcal {F}_{t^-})=\text {Var}(dN_{ik}(t)|\mathcal {F}_{t^-})\\ &=\mathbb {E}(dN_{ik}(t)|\mathcal {F}_{t^-})(1-\mathbb {E}(dN_{ik}(t)|\mathcal {F}_{t^-}))=Y_i(t)dH_k(t;X_i), \end{aligned}$$

where we regard *dt · dt* as 0. Furthermore,

$$\begin{aligned} d\langle M_{ik}, M_{i'k'} \rangle (t)&\equiv \text {Cov}(dM_{ik}(t)dM_{i'k'}(t)|\mathcal {F}_{t^-})=\text {Cov}(dN_{ik}(t)dN_{i'k'}(t)|\mathcal {F}_{t^-})\\&=\mathbb {E}(dN_{ik}(t)dN_{i'k'}(t)|\mathcal {F}_{t^-})-\mathbb {E}(dN_{ik}(t)|\mathcal {F}_{t^-})\mathbb {E}(dN_{i'k'}(t)|\mathcal {F}_{t^-})\\&=\mathbb {P}(dN_{ik}(t)=1,dN_{i'k'}(t)=1|\mathcal {F}_{t^-})=0 \end{aligned}$$

for $\{i, k\} \ne \{i', k'\}$ due to the continuity of the random variables (at most one jump at *t* among all *n* subjects) and the fact that a single subject cannot experience more than two risks.

### Estimation

We now introduce the estimation procedure for the proposed model. For $f \in \mathcal {H}$, the negative log-partial likelihood for the *k*-th risk is

$$\begin{aligned} \mathcal {L}_{k(n)}(f)=-\int _0^\tau \frac{1}{n}\sum _{i=1}^n\left\{ f(X_i)-\ln \left( \frac{1}{n}\sum _{i'=1}^nY_{i'}(t) \exp (f(X_{i'})) \right) \right\} dN_{ik}(t). \end{aligned}$$

To represent *f*, traditional smoothing splines require a full basis of spline functions with knots at all the observations. When *n* is large, this can generate significant computational burden. To address this issue, Kim and Gu (2004) proposed to use only $q_k$ observations as knots while preserving the same convergence rate as the full basis expansion. For each *k*, we randomly select $q_k$ subjects to serve as knots, so that the knot locations represent the overall covariate distribution without favoring particular subjects. Let their covariates be $\{X_{\ell (k)}\}_{\ell =1}^{q_k}$ $\subseteq \{X_i\}_{i=1}^n$. Then, $\mathcal {H}_J^{(k)} = \text {Span}\{\mathcal {K}_{J_k}(X_{1(k)},\cdot ),\ldots ,\mathcal {K}_{J_k}(X_{q_k(k)},\cdot )\}$ forms a finite-dimensional subspace of $\mathcal {H}_J$, which further defines $\mathcal {H}^{(k)} = \mathcal {N}_J \oplus \mathcal {H}_J^{(k)}$, a finite-dimensional subspace of $\mathcal {H}$. Note that $q_k$ and the selected $q_k$ subjects can be different for each *k*. For each risk *k*, using the efficient approximation (Kim and Gu 2004), we define the estimator of $\eta ^*_k$ to be

1 $$\begin{aligned} \hat{\eta }_k=\underset{f \in \mathcal {H}^{(k)} }{\text {argmin }} \mathcal {L}_{k(n)}(f)+ \frac{\lambda _k}{2} J_k(f), \end{aligned}$$

where $J_k(f)= J_k(f,f)$ is a roughness penalty and $\lambda _k \in (0,\infty )$ is a smoothing parameter. When $q_k=n$, we have $\hat{\eta }_k = \hat{\eta }^*_k \equiv \underset{f \in \mathcal {H}}{\text {argmin }} \mathcal {L}_{k(n)}(f)+ {\lambda _k} J_k(f)/2$ by the representer theorem.

The null space $\mathcal {N}_J$ generally consists of lower-degree polynomials and is finite dimensional. Let $\phi _1,\ldots ,\phi _p$ be the basis functions of $\mathcal {N}_J$. Then estimator $\hat{\eta }_k$ can have the expression $\hat{\eta }_k=\sum _{l=1}^p \hat{d}_{k,l} \phi _l + \sum _{\ell =1}^{q_k} \hat{c}_{k,\ell }\mathcal {K}_{J_k}(X_{\ell (k)},\cdot )$. And the optimization in (1) becomes

2 $$\begin{aligned} (\hat{\boldsymbol{d}}_k^{{\top }},\hat{\boldsymbol{c}}_k^{{\top }})^{{\top }}= \underset{\boldsymbol{d}_k\in \mathbb {R}^p, \boldsymbol{c}_k\in \mathbb {R}^{q_k}}{\text {argmin }} \mathcal {L}_{k(n)}\left( \sum _{l=1}^p d_{k,l} \phi _l + \sum _{\ell =1}^{q_k} c_{k,\ell }\mathcal {K}_{J_k}(X_{\ell (k)},\cdot ) \right) + \frac{\lambda _k}{2} \boldsymbol{c}_k^{{\top }} \boldsymbol{\mathcal {K}}_{J_k} \boldsymbol{c}_k, \end{aligned}$$

where $\boldsymbol{\mathcal {K}}_{J_k}=\{\mathcal {K}_{J_k}(X_{\ell (k)},X_{\ell '(k)})\}_{\ell ,\ell '\in \{1,\ldots ,q_k\}}$ is the $q_k\times q_k$ kernel matrix.

Here, two modeling choices are required. The first is the order *m* of $\mathcal {H}_{[j]}$ for continuous covariates, where a larger *m* corresponds to smoother spline functions. A common choice is *m=2* (Gu 2013), corresponding to cubic smoothing splines. The second is the number of knots $q_k$. As shown in Kim and Gu (2004), $q_k\asymp n^{2/9}$ is sufficient to guarantee the same convergence rate of the function estimate when tensor product cubic splines are used.

Since $q_k$ grows at a slower rate than *n*, the computational complexity of the proposed efficient approximation estimator in (2) is $\mathcal {O}(n q_k^2)$, which is substantially lower than the $\mathcal {O}(n^3)$ complexity of the full-knot estimator even for large sample sizes, supporting the computational efficiency emphasized in the introduction. In addition, the objective function in (2) is convex with a quadratic roughness penalty, yielding a well-behaved optimization problem that can be solved reliably using standard numerical procedures.

### Bayesian confidence intervals

We now introduce Bayesian pointwise confidence intervals for the functional parameters in our model. The name “Bayesian" was coined by Wahba (1983) since the roughness penalty can be treated like a Gaussian prior, making the penalized likelihood interpretable as the posterior likelihood and its optimizer as the posterior mode in Bayesian analysis. For each * k *, define $\boldsymbol{\varphi }_k(\cdot ) = [\phi _1(\cdot ), \ldots , \phi _p(\cdot ), \mathcal {K}_{J_k}(X_{1(k)}, \cdot ), \ldots ,$ $\mathcal {K}_{J_k}(X_{q_k(k)}, \cdot ) ]^{{\top }}$ and the hessian matrix for (2) as

$$\begin{aligned} & \boldsymbol{V}_{k(n)}(\hat{\eta }_k)= \int _0^\tau \left\{ \frac{S_{k(n)}^{(2)}(\hat{\eta }_k,t)}{S_{k(n)}^{(0)}(\hat{\eta }_k,t)} -\frac{S_{k(n)}^{(1)}(\hat{\eta }_k,t)}{S_{k(n)}^{(0)}(\hat{\eta }_k,t)}\frac{S_{k(n)}^{(1)}(\hat{\eta }_k,t)^{{\top }}}{S_{k(n)}^{(0)}(\hat{\eta }_k,t)} \right\} d\overline{N}_k(t)\\ & \quad + \text {Diag}\left( \boldsymbol{O}_p,\lambda _k\boldsymbol{\mathcal {K}}_{J_k}\right) , \end{aligned}$$

where $S_{k(n)}^{(0)}(\hat{\eta }_k,t)=\sum _{i=1}^nY_i(t) \exp (\hat{\eta }_k(X_i))/n$, $S_{k(n)}^{(1)}(\hat{\eta }_k,t)=\sum _{i=1}^nY_i(t) \boldsymbol{\varphi }_k(X_i)$ $\exp (\hat{\eta }_k(X_i))/n$, $S_{k(n)}^{(2)}(\hat{\eta }_k,t)=\sum _{i=1}^nY_i(t) \boldsymbol{\varphi }_k(X_i) \boldsymbol{\varphi }_k(X_i)^{{\top }} \exp (\hat{\eta }_k(X_i))/n$, $\overline{N}_k(\cdot )=\sum _{i=1}^n N_{ik}(\cdot )/n$, and $\boldsymbol{O}_p$ is a *p× p* zero matrix. Then the $100 (1 - \alpha ) \%$ confidence interval for $\eta ^*_k(x)$ at $x \in \mathcal {X}$ is given by

$$\begin{aligned} \hat{\eta }_k(x) \pm z_{1-\alpha /2} \sqrt{\frac{1}{n} \boldsymbol{\varphi }_k(x)^{\top } \boldsymbol{V}_{k(n)}^{-1}(\hat{\eta }_k) \boldsymbol{\varphi }_k(x)}, \end{aligned}$$

where $z_{1-\alpha /2}$ is the *(1 - α /2)*-quantile of the standard normal distribution.

### Smoothing parameter selection

We propose a criterion for smoothing parameter selection. For each *k*, let $n_k = \sum _{i=1}^n N_{ik}(\tau )$ represent the number of subjects who failed due to the *k*-th risk factor. Let $\{Z_{\ell (k)}\}_{\ell =1}^{n_k} \subseteq \{X_i\}_{i=1}^n$ be the subset of $n_k$ covariates out of *n* that satisfy $N_{ik}(\tau ) = 1$.

Using the relative Kullback–Leibler distance (RKL) proxy for leave-one-out cross validation, as outlined in Du et al. (2010), we select the smoothing parameter $\lambda _k$ that minimizes

3 $$\begin{aligned} \text {RKL}_k(\lambda _k)=\frac{n}{n_k} \mathcal {L}_{k(n)}(\hat{\eta }_k) + \frac{\alpha _k}{n_k(n_k - 1)} \text {tr}\left( \boldsymbol{P}_k^{{\top }} \boldsymbol{Q}_k \boldsymbol{V}_{k(n)}^{-1}(\hat{\eta }_k) \boldsymbol{Q}_k^{{\top }} \boldsymbol{P}_k \right) , \end{aligned}$$

where $\boldsymbol{P}_k = \boldsymbol{I}_{n_k} - \boldsymbol{1}_{n_k}\boldsymbol{1}_{n_k}^{{\top }} / n_k$, $\boldsymbol{I}_{n_k}$ is $n_k \times n_k$ identity matrix, $\boldsymbol{1}_{n_k}$ is length $n_k$ one vector, $\boldsymbol{Q}_k = [\boldsymbol{\varphi }_k(Z_{1(k)}), \ldots , \boldsymbol{\varphi }_k(Z_{n_k(k)})]^{{\top }}$, and *α >1* is a preselected constant to guard against under-smoothing. We set $\alpha _k = 1.4$ following the recommendation in Gu (2013).

### Rates of convergence

We now present the rate of convergence result for our estimator in (1). For notational simplicity, we assume $\mathcal {X}_{[j]} = [0,1]$ for all *j*, noting that any additional categorical domain $\mathcal {X}_{[j]}$ only adds a finite dimensional component space $\mathcal {H}_{[j]}$ which does not affect the convergence rates derived here. The notation and the assumptions are provided in Sects. A and B of the Appendix, respectively.

For each *k*, define a non-negative definite bivariate functional $\mathcal {V}_k(\cdot ,\cdot )$ on $\mathcal {H}$ by

$$\begin{aligned} \mathcal {V}_k(f,g)=\int _0^\tau \left\{ \frac{S(fg;\eta ^*_k,t)}{S(\eta ^*_k,t)} -\frac{S(f;\eta ^*_k,t)}{S(\eta ^*_k,t)}\frac{S(g;\eta ^*_k,t)}{S(\eta ^*_k,t)} \right\} S(\eta ^*_k,t)dH_{0k}(t) \end{aligned}$$

for $f, g \in \mathcal {H}$, where $S(\eta ,t)\equiv \mathbb {E}_W[Y(t)\exp (\eta (X))]$, $S(f;\eta ,t)\equiv \mathbb {E}_W[Y(t)f(X)\exp (\eta (X))]$, and $S(fg;\eta ,t)\equiv \mathbb {E}_W[Y(t)f(X)g(X)\exp (\eta (X))]$ for $f, g, \eta \in \mathcal {H}$ and $t\in [0,\tau ]$. Let $\mathcal {V}_k(f)=\mathcal {V}_k(f,f)$ for $f \in \mathcal {H}$.

#### Theorem 1

Under Assumption B.1 in Sect. B of the Appendix, for each *k*, as $n\rightarrow \infty$, we have

$$\sqrt{\left( \mathcal {V}_k+\lambda _k J_k\right) \left( \hat{\eta }_k-\eta ^*_k\right) }=\mathcal {O}_{\mathbb {P}}\left( n^{-1/2}\lambda _k^{-1/(2r)}+\lambda _k^{b_k/2}\right) =o_{\mathbb {P}}(1).$$

The proof of Theorem 1 is provided in section S1 of the Supplementary. One can show that Theorem 1 implies $\Vert \hat{\eta }_k-\eta ^*_k\Vert _{\mathcal {L}_2}=\mathcal {O}_{\mathbb {P}}\left( n^{-1/2}\lambda _k^{-1/(2r)}+\lambda _k^{b_k/2}\right)$, where $\Vert \cdot \Vert _{\mathcal {L}_2}$ represents the $\mathcal {L}_2$ norm. Here *r > 1* characterizes the model parameter space $\mathcal {H}$. For example, *r = 2m* when *d = 1* or *d > 1* with an additive model; and *r = 2m - δ * when $\mathcal {H}$ is a tensor product space for a model with interactions, where *δ >0* can be arbitrarily small. See Gu (2013) for additional details. The constant $b_k \in [1,2]$ represents the smoothness level of the true function $\eta ^*_k$, with $b_k >1$ corresponding to $\eta ^*_k$ being super-smooth. The optimal rate of convergence is $\mathcal {O}_{\mathbb {P}}(n^{-rb_k/(2rb_k+2)})$, achievable by setting $\lambda _k \asymp n^{-r/(rb_k+1)}$. Note that this rate coincides with the optimal convergence rate for nonparametric function estimation in general.

## Simulation study

### Simulation study for additive models

We perform a simulation study for the proposed method under additive covariate effects. We set *K = 2*, *m=2*, *d = 3*, and impose an additive structure on $\mathcal {H}$; that is, for each *k*, we assume $\eta _k^*= \sum _{j=1}^d \eta ^*_{k,\{j\}}$, where

$$\begin{aligned} {\eta }^*_{1,\{1\}}(x_{[1]})&= 8.752x_{[1]}^2 -6.126x_{[1]} +0.146, \\ {\eta }^*_{1,\{2\}}(x_{[2]})&= 1.163\log \!\left( 0.667x_{[2]}^2 -1.333x_{[2]} +0.767 \right) +8.721x_{[2]}^2 -8.721x_{[2]} +3.006, \\ {\eta }^*_{1,\{3\}}(x_{[3]})&= -3.464(x_{[3]}-0.5), \\ {\eta }^*_{2,\{1\}}(x_{[1]})&= 12.005\exp (-x_{[1]}) -16.807x_{[1]}^2 +23.530x_{[1]} -13.751, \\ {\eta }^*_{2,\{2\}}(x_{[2]})&= 3.464(x_{[2]}-0.5), \\ {\eta }^*_{2,\{3\}}(x_{[3]})&= 11.600x_{[3]}^2 -9.860x_{[3]} +1.063. \end{aligned}$$

Note that some effect functions are adopted from Cho and Liu (2026). In addition, some effect functions are linear, reflecting the findings observed in the real data application presented in Sect. 4.

We assume a Weibull baseline cause-specific hazard functions; that is, for each *k*, $h_{0k}(t) = {\gamma _k}t^{\gamma _k - 1}/{\beta _k^{\gamma _k}}$. We set $\gamma _1 = \gamma _2 = 1.4$ and $\beta _1 = \beta _2 = 1.7$. Similarly, the hazard function for the underlying censoring time is also assumed to be Weibull; that is, for a random variable $\tilde{C}\in (0,\infty )$, we have $C=\min \{\tilde{C},\tau \}$, where $h_c(t) = {\gamma _c}t^{\gamma _c - 1}/{\beta _c^{\gamma _c}}$ and $dH_c(t)=h_c(t)dt = \mathbb {P}(\tilde{C} \in [t, t + dt) \mid \tilde{C} \ge t)$. We set *τ = 1*.

Let $\tilde{X} = (\tilde{X}_{[1]}, \tilde{X}_{[2]}, \tilde{X}_{[3]})^{\top }$ be a multivariate normal random variable with mean vector $(0, 0, 0)^{\top }$ and covariance matrix $\{0.5^{|j - j'|}\}_{1 \le j, j' \le 3}$. We have $X = (X_{[1]}, X_{[2]}, X_{[3]})^{\top }$ $= (\varPhi (\tilde{X}_{[1]}), \varPhi (\tilde{X}_{[2]}), \varPhi (\tilde{X}_{[3]}))^{\top }$, where *\varPhi * denotes the distribution function of the standard normal distribution. Each $X_{[j]}$ follows a uniform distribution on [0, 1] and $X_{[1]},X_{[2]},X_{[3]}$ are dependent.

Considering that $\mathbb {P}(\tilde{G}=k|X,\tilde{T}\in [t,t+dt))=h_k(t;X)/h(t;X)$ for each *k*, we generate simulation data following Beyersmann et al. (2009). Specifically, for each subject *i*, (1) generate $C_i$; (2) generate $X_i$; (3) generate $\tilde{T}_i$ from $\textsf{F}(t; X_i)$; (4) generate $\tilde{G}_i$ from

$$\text {Multinomial} \big (1, {h_1(\tilde{T}_i; X_i)}/{h(\tilde{T}_i; X_i)}, \ldots , {h_K(\tilde{T}_i; X_i)}/{h(\tilde{T}_i; X_i)} \big );$$

(5) compute $$T_i$$, $$\mathscr {I}_i$$, and $$G_i$$.

For the simulation setting, we vary the censoring levels by $(\gamma _c, \beta _c) \in \{ (0.8, 0.7), (1.2, 1.1), (1.6, 1.5) \}$ and the sample size $n \in \{250, 500, 1000\}$, where smaller $\gamma _c$ or smaller $\beta _c$ induces a heavier censoring rate. We repeat the simulation over 1000 replicates, where, for each replicate, we compute $\hat{\eta }_k$ for each *k*. For each simulation setting, we evaluate the performance of the proposed method across all replicates, focusing on the rate of convergence and the Bayesian confidence interval.

Table 1 presents the summary of simulated data by settings. One can observe that events are approximately equally likely to occur for the two risks and that smaller values of $\gamma _c$ or $\beta _c$ result in a higher proportion of censored observations.

**Fig. 1:**
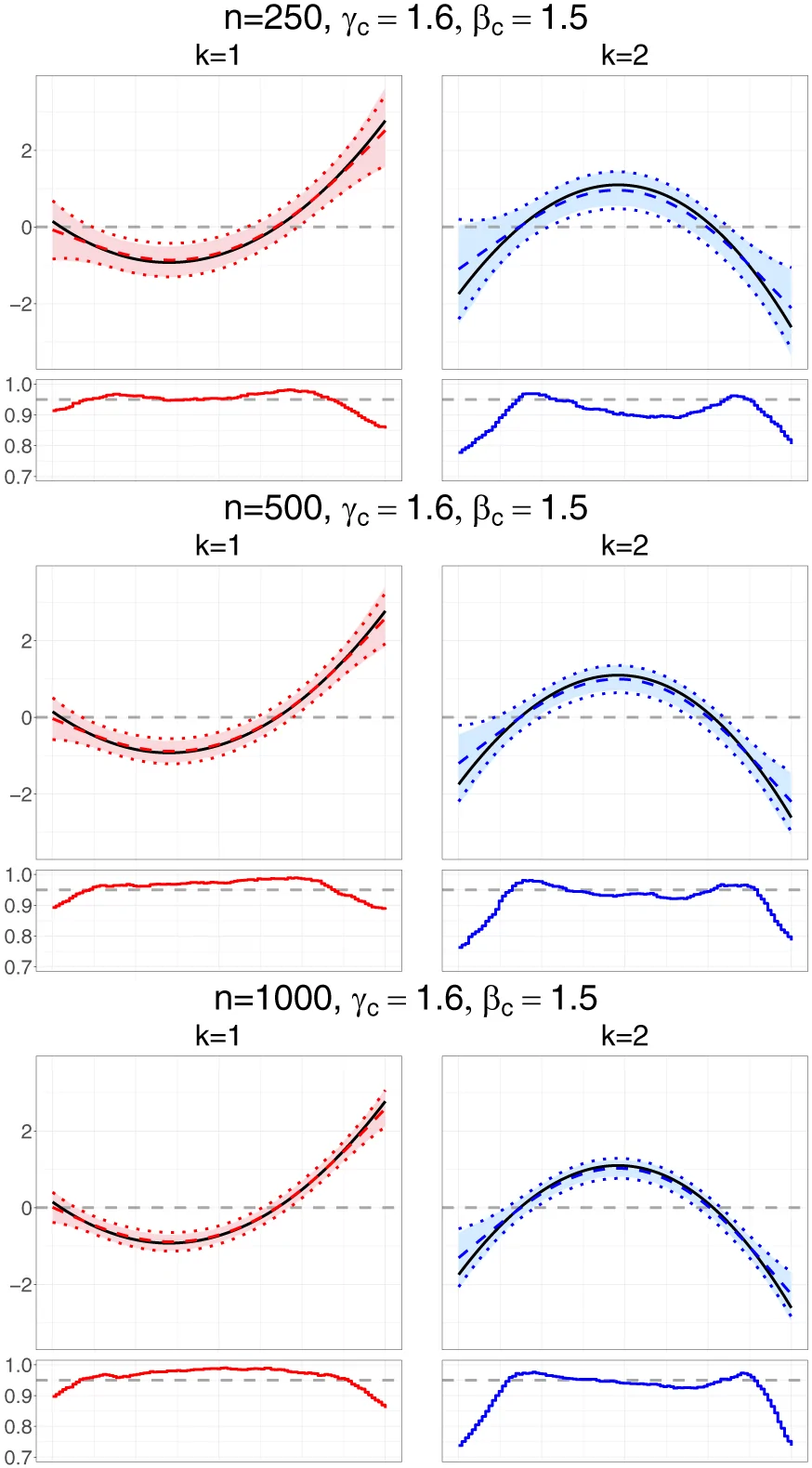
Simulation results for $\mathcal {X}_{[1]}$. For each simulation setting and each risk *k*, the top panel shows $\eta ^*_{k,\{1\}}$ (solid line), average of $\hat{\eta }_{k,\{1\}}$ (dashed line), average of the 95% Bayesian confidence interval for $\eta ^*_{k,\{1\}}$ (dotted lines), and 2.5 and 97.5% empirical quantiles of $\hat{\eta }_{k,\{1\}}$ (shaded area). The bottom panel displays the empirical coverage rate of the 95% Bayesian confidence interval for $\eta ^*_{k,\{1\}}$. Note that all averages and empirical quantiles are computed point-wise across all replicates

**Fig. 2:**
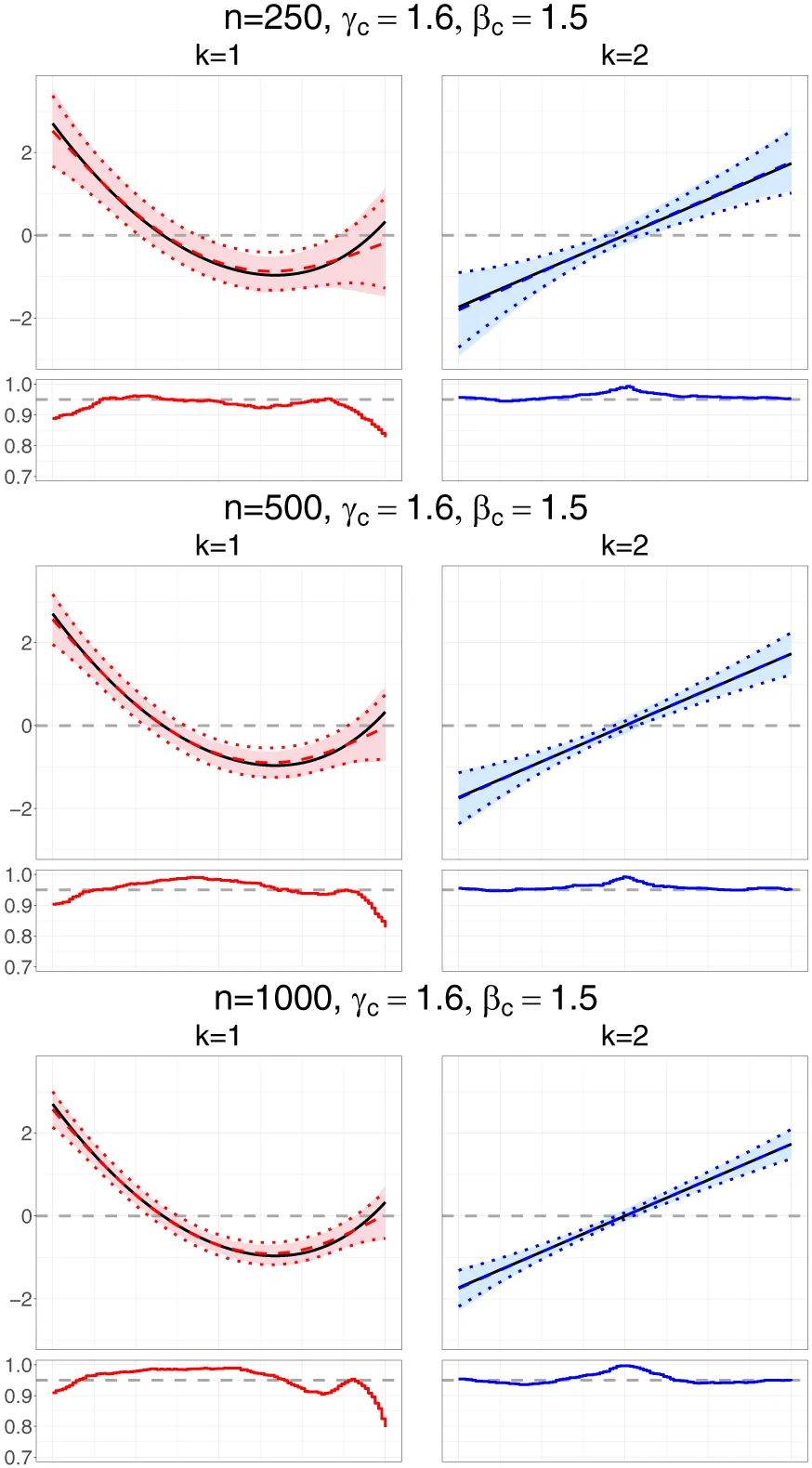
Simulation results for $\mathcal {X}_{[2]}$. For each simulation setting and each risk *k*, the top panel shows $\eta ^*_{k,\{2\}}$ (solid line), average of $\hat{\eta }_{k,\{2\}}$ (dashed line), average of the 95% Bayesian confidence interval for $\eta ^*_{k,\{2\}}$ (dotted lines), and 2.5 and 97.5% empirical quantiles of $\hat{\eta }_{k,\{2\}}$ (shaded area). The bottom panel displays the empirical coverage rate of the 95% Bayesian confidence interval for $\eta ^*_{k,\{2\}}$. Note that all averages and empirical quantiles are computed point-wise across all replicates

**Fig. 3:**
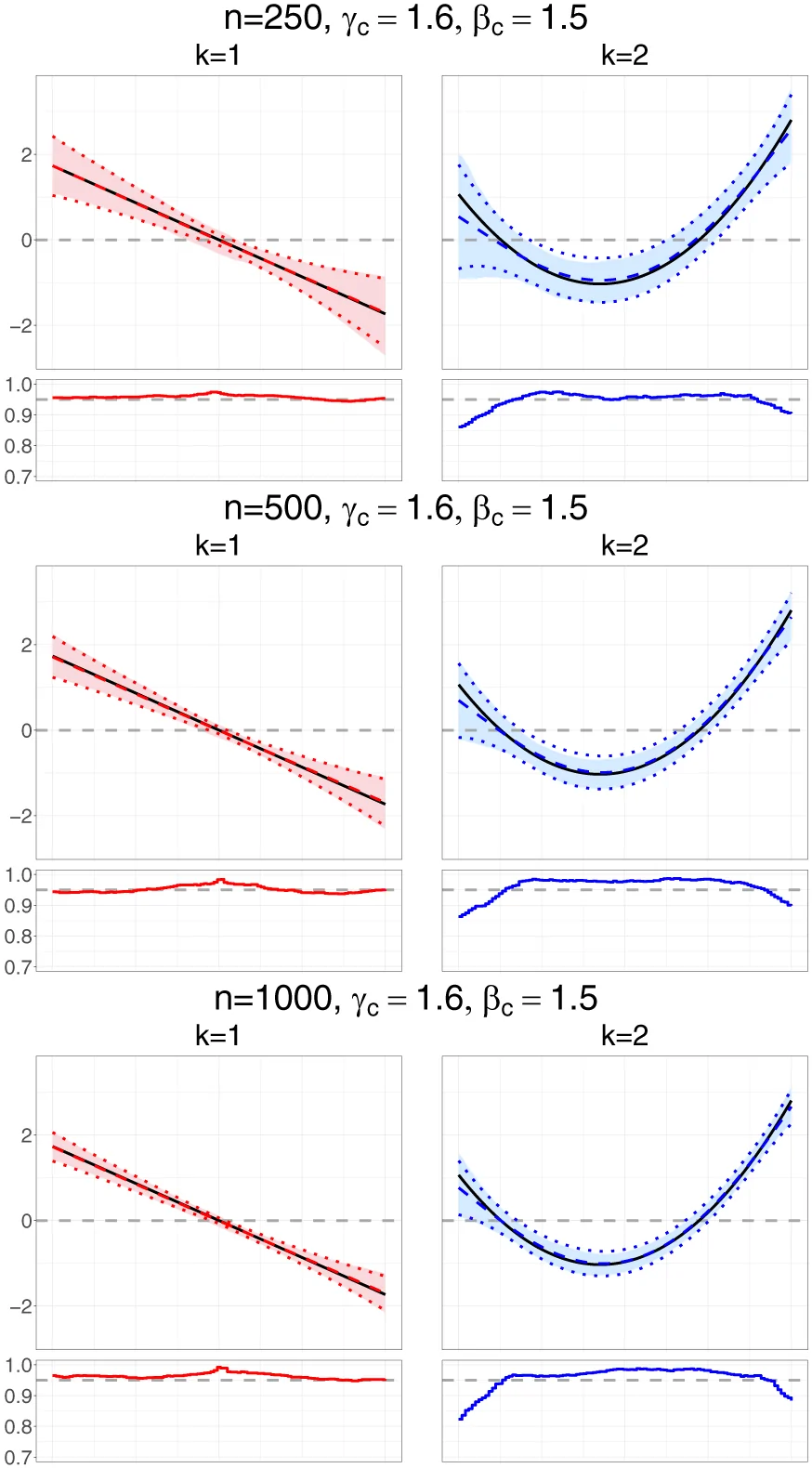
Simulation results for $\mathcal {X}_{[3]}$. For each simulation setting and each risk factor, the top panel shows $\eta ^*_{k,\{3\}}$ (solid line), average of $\hat{\eta }_{k,\{3\}}$ (dashed line), average of the 95% Bayesian confidence interval for $\eta ^*_{k,\{3\}}$ (dotted lines), and 2.5 and 97.5% empirical quantiles of $\hat{\eta }_{k,\{3\}}$ (shaded area). The bottom panel displays the empirical coverage rate of the 95% Bayesian confidence interval for $\eta ^*_{k,\{3\}}$. Note that all averages and empirical quantiles are computed point-wise across all replicates

Estimation performances are reported in Table 1, while both estimation performances and the Bayesian confidence interval performances are shown in Figs. 1, 2, and 3 and Figs. S1–S6 in section S3 of the Supplementary. As expected, larger sample sizes or lower censoring levels yield improved estimation accuracy. Larger sample sizes also lead to narrower confidence intervals and generally better coverage rates. The proposed Bayesian confidence intervals align closely with empirical quantiles across replicates, demonstrating their reliability.

**Table 1:** The first subtable reports the simulated data summaries by settings, and the second subtable reports the $\mathcal {L}_2$ losses by simulation settings

*n*	$h_c$		Data summary		
	$\gamma _c$	$\beta _c$	$\sum _{i=1}^n1\!\!1(G_i=0)$	$\sum _{i=1}^n1\!\!1(G_i=1)$	$\sum _{i=1}^n1\!\!1(G_i=2)$
250	0.8	0.7	134.89 (7.92)	58.18 (6.45)	56.93 (6.72)
	1.2	1.1	101.55 (8.05)	75.16 (7.29)	73.28 (7.16)
	1.6	1.5	80.19 (7.33)	86.20 (7.65)	83.62 (7.56)
500	0.8	0.7	269.81 (11.27)	115.78 (9.26)	114.41 (9.40)
	1.2	1.1	203.51 (10.67)	149.65 (10.46)	146.84 (10.26)
	1.6	1.5	160.77 (10.91)	171.12 (11.02)	168.11 (10.85)
1000	0.8	0.7	539.63 (16.17)	231.99 (13.36)	228.38 (13.66)
	1.2	1.1	407.71 (15.64)	299.42 (14.44)	292.87 (14.13)
	1.6	1.5	321.45 (14.90)	342.70 (15.44)	335.85 (15.12)

*n*	$h_c$		$\Vert \hat{\eta }_{k,\{j\}}-\eta ^*_{k,\{j\}}\Vert _{\mathcal {L}_2}$					
	$\gamma _c$	$\beta _c$	*j=1*		*j=2*		*j=3*	
			*k=1*	*k=2*	*k=1*	*k=2*	*k=1*	*k=2*
250	0.8	0.7	0.23 (0.11)	0.34 (0.15)	0.29 (0.15)	0.20 (0.18)	0.18 (0.15)	0.29 (0.16)
	1.2	1.1	0.20 (0.10)	0.30 (0.13)	0.25 (0.11)	0.18 (0.14)	0.16 (0.14)	0.25 (0.12)
	1.6	1.5	0.20 (0.09)	0.28 (0.11)	0.23 (0.10)	0.16 (0.12)	0.16 (0.12)	0.24 (0.11)
500	0.8	0.7	0.17 (0.08)	0.25 (0.10)	0.20 (0.09)	0.14 (0.11)	0.13 (0.09)	0.20 (0.09)
	1.2	1.1	0.15 (0.06)	0.22 (0.08)	0.18 (0.07)	0.12 (0.09)	0.11 (0.09)	0.18 (0.07)
	1.6	1.5	0.14 (0.06)	0.21 (0.08)	0.17 (0.07)	0.11 (0.08)	0.10 (0.08)	0.17 (0.07)
1000	0.8	0.7	0.12 (0.05)	0.19 (0.07)	0.15 (0.05)	0.10 (0.08)	0.09 (0.07)	0.15 (0.06)
	1.2	1.1	0.11 (0.04)	0.17 (0.06)	0.13 (0.05)	0.08 (0.07)	0.08 (0.06)	0.13 (0.05)
	1.6	1.5	0.10 (0.04)	0.16 (0.06)	0.12 (0.05)	0.08 (0.06)	0.08 (0.06)	0.12 (0.05)

Each cell shows the mean (standard deviation) over 1000 replicates

### Simulation study for models with interactions

We conduct a simulation study to evaluate the finite-sample performance of the proposed method in the presence of interaction effects. We set *K=2*, *m=2*, and *d=2*, and assume a tensor-product structure on $\mathcal {H}$. Specifically, for each *k=1,2*, we consider

$$ \eta _k^*= \eta ^*_{k,\{1\}} + \eta ^*_{k,\{2\}} + \eta ^*_{k,\{1,2\}}. $$

The simulation study encompass a variety of effect structures, including nonlinear, linear, and categorical main effects, as well as nonlinear-by-nonlinear, nonlinear-by-categorical, and linear-by-categorical interactions. Several effect functions are adopted from Cho and Liu (2026). We consider two tensor-product effect structures.

Tensor-Product Structure 1. Let $\mathcal {X}_{[1]}=\mathcal {X}_{[2]}=[0,1]$. Both covariates are continuous. The effect functions are specified as

$$\begin{aligned} \eta _{1,\{1\}}^*(x_{[1]})&= 11.600x_{[1]}^2 -9.860x_{[1]} +1.063,\\ \eta _{1,\{2\}}^*(x_{[2]})&= 12.005\exp (-x_{[2]}) -16.807x_{[2]}^2 +23.530x_{[2]} -13.751,\\ \eta _{1,\{1,2\}}^*(x_{[1]},x_{[2]})&= -32.863x_{[1]}^2x_{[2]} +32.863x_{[1]}x_{[2]}^2 +16.432x_{[1]}^2 -16.432x_{[2]}^2\\&\quad -10.955x_{[1]} +10.955x_{[2]},\\ \eta _{2,\{1\}}^*(x_{[1]})&= \sqrt{12}(x_{[1]}-0.5),\\ \eta _{2,\{2\}}^*(x_{[2]})&= 1.163\log \left( 0.667x_{[2]}^2 -1.333x_{[2]} +0.767 \right) +8.721x_{[2]}^2 -8.721x_{[2]} +3.006,\\ \eta _{2,\{1,2\}}^*(x_{[1]},x_{[2]})&= -3.867x_{[1]}^2x_{[2]}^2 +25.522x_{[1]}^2x_{[2]} -17.788x_{[1]}x_{[2]}^2 +5.156x_{[1]}x_{[2]}\\&\quad -11.472x_{[1]}^2 +10.183x_{[2]}^2 +3.351x_{[1]} -11.085x_{[2]} +2.148. \end{aligned}$$

This structure consists of two continuous covariates. All main and interaction effects are nonlinear except for $\eta _{2,\{1\}}^*$, which is linear.

Tensor-Product Structure 2. Let $\mathcal {X}_{[1]}=[0,1]$ and $\mathcal {X}_{[2]}=\{1,2\}$, where the second covariate is binary. The effect functions are specified as

$$\begin{aligned} \eta _{1,\{1\}}^*(x_{[1]})&= 11.600x_{[1]}^2-9.860x_{[1]}+1.063,\\ \eta _{1,\{2\}}^*(x_{[2]})&= -1\!\!1(x_{[2]}=1)+1\!\!1(x_{[2]}=2),\\ \eta _{1,\{1,2\}}^*(x_{[1]},x_{[2]})&= -(11.600x_{[1]}^2-9.860x_{[1]}+1.063)1\!\!1(x_{[2]}=1)\\&\quad + (11.600x_{[1]}^2-9.860x_{[1]}+1.063)1\!\!1(x_{[2]}=2),\\ \eta _{2,\{1\}}^*(x_{[1]})&= 3.464(x_{[1]}-0.5),\\ \eta _{2,\{2\}}^*(x_{[2]})&= 1\!\!1(x_{[2]}=1)-1\!\!1(x_{[2]}=2),\\ \eta _{2,\{1,2\}}^*(x_{[1]},x_{[2]})&= 3.464(x_{[1]}-0.5)1\!\!1(x_{[2]}=1)- 3.464(x_{[1]}-0.5)1\!\!1(x_{[2]}=2). \end{aligned}$$

This structure contains a nonlinear main effect, a categorical main effect, and a nonlinear-by-categorical interaction for Risk 1. For Risk 2, it contains a linear main effect, a categorical main effect, and a linear-by-categorical interaction.

Except for the tensor-product structure and the effect functions, the simulation data for structures 1 and 2 are generated in the same manner as in Sect. 3.1, with *d=2* instead of *d=3*. We consider $(\gamma _c,\beta _c)\in \{(0.8,0.7),(1.2,1.1),$ *(1.6,1.5)\}* and sample sizes $n\in \{250,500,1000\}$. For each configuration, 1,000 replicates are generated.

Computational time was evaluated under Structure 1 with *n=250* and *500*, $(\gamma _c,\beta _c)=(1.6,1.5)$, and 50 replicates. All simulations were conducted on an Apple Mac equipped with an Apple M1 processor and 16 GB RAM. The average computational times were 17 seconds for *n=250* and 97 s for *n=500*.

Tables S1–S4 in Section S3 of the Supplementary summarize the simulation results for Tensor-Product Effect Structures 1 and 2, respectively, including data summaries, estimation performances, and the coverage probabilities and lengths of the Bayesian confidence intervals. Overall, the proposed method performs well across a variety of effect structures. As the sample size *n* increases, for many cases, the estimation accuracy improves, the Bayesian confidence intervals become narrower, and the empirical coverage probabilities increases. As the censoring rate decreases, estimation accuracy improves in many cases, although the improvement is generally less pronounced than that resulting from an increase in the sample size *n*. Although the $\mathcal {L}_2$ losses may differ across effects, this is largely attributable to differences in the scales of the underlying effect functions. In addition, linear, categorical, and interactions involving categorical covariates often exhibit better coverage performance than nonlinear effects, which is expected due to their lower complexity. Nevertheless, the proposed method demonstrates satisfactory estimation and the Bayesian interval inference performance across many tensor-product effect structures.

## Real application

In this section, we analyze the Multiple Myeloma Research Foundation (MMRF) CoMMpass study, which provides clinical trial data and gene expression data, publicly available through the Genomic Data Commons (GDC) portal. Multiple myeloma is a type of blood cancer that originates in plasma cells and affects the bone marrow and immune function. The dataset includes, for each subject, time-to-event or censoring, a censoring indicator, cause of death (categorized as multiple myeloma-related or non-cancer-related), age, and gene expression profiles for various genes.

Most previous studies on time-to-event modeling in multiple myeloma have either used linear survival models (Simhal et al. 2023; Zhang et al. 2023), which focus on variable selection or interpretation but cannot accommodate nonlinear effects; or machine learning approaches (Shah et al. 2025; Ren et al. 2023), which aim for predictive performance but lack interpretability, especially with respect to how each covariate nonlinearly influences the event time. The study by Zhao et al. (2024), which focuses on survival modeling with omics covariates using The Cancer Genome Atlas (TCGA) data—also available through the GDC portal and formatted similarly to the MMRF CoMMpass data—mentions competing risks modeling as a potential approach for analyzing multiple causes of death, such as cancer-related and other-cause mortality. This suggestion motivates our use of competing risks modeling in the MMRF CoMMpass data analysis.

Despite the richness of the MMRF CoMMpass study, existing literature lacks nonparametric modeling frameworks that can flexibly capture nonlinear covariate effects, as well as jointly model multiple causes of death. Our proposed method addresses both limitations to analyze the MMRF CoMMpass data.

Under a competing risks framework, following previous studies on multiple myeloma (Shah et al. 2025; Simhal et al. 2023; Zhang et al. 2023), we focus on the effect of gene expression as covariates. Note that age is also an important factor (Zhao et al. 2024). After excluding subjects with missing age information, the numbers of observations in the censored group and two risk groups are respectively 577, 83 (multiple myeloma-related deaths), and 66 (non-multiple myeloma-related deaths).

The original gene expression data contain measurements on 60,660 genes. We preprocess the gene expression data by a logarithmic transformation and a scaling of each variable to [0, 1]. To reduce dimensionality, we first select the top 25 genes with the highest standard deviation among those with less than 10% missing values.

From this subset, with age (*j=1*) included as a covariate, gene covariates were added sequentially according to decreasing values of $\sum _{k=1}^2 \Vert \hat{\eta }_k\Vert _{\mathcal {L}_2}$, where $\hat{\eta }_k$ is the *k*th cause-specific log relative risk estimate from the proposed method with one covariate in the model at a time. Covariates were added until $\text {RKL}_1+\text {RKL}_2$ in (3), which serves as a criterion analogous to a cross-validation error (Gu 2013), began to increase. The value of $\text {RKL}_1+\text {RKL}_2$ decreases up to *d=6* and increases thereafter. Therefore, we selected *d=6* covariates. The selected genes are RPS6KA6 (*j=2*), IGKC (*j=3*), UCHL1 (*j=4*), DSG2 (*j=5*), and RPS4Y1 (*j=6*), listed in decreasing order of $\sum _{k=1}^2 \Vert \hat{\eta }_k\Vert _{\mathcal {L}_2}$.

The final dataset, after excluding subjects with missing values for any of the three selected genes, consists of 498 censorings, 67 multiple myeloma-related deaths, and 56 non-multiple myeloma-related deaths, with *d=6* covariates, consisting of age and the selected gene expression covariates described above. We fit our proposed model to this dataset, assuming an additive structure on $\mathcal {H}$ and setting *m=2*.

**Fig. 4:**
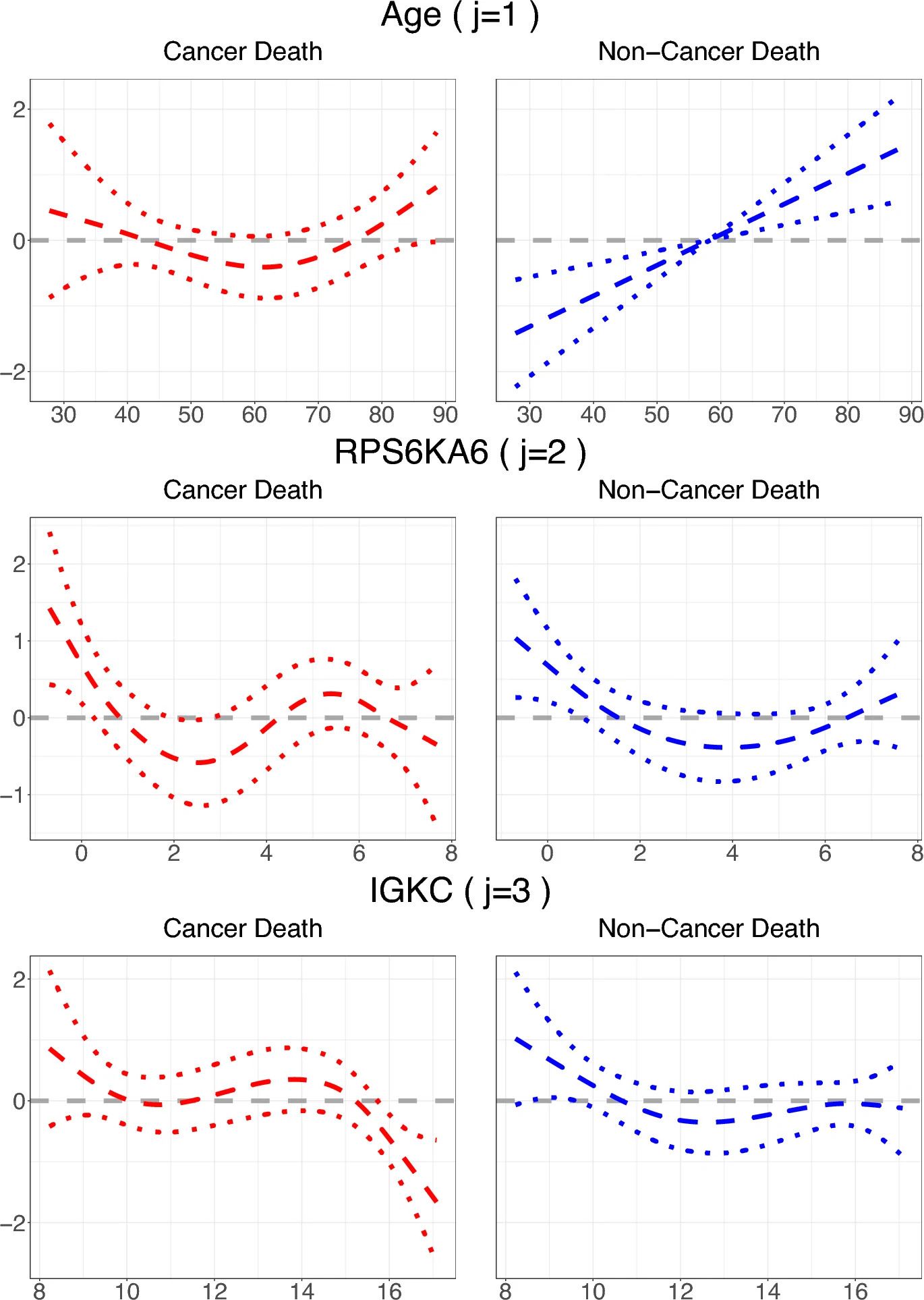
MMRF data analysis results. For each row, the title represents the covariate and the subtitles correspond to the associated risks. For each plot, the dashed line represents $\hat{\eta }_{k,\{j\}}$ and the dotted lines indicate the 95% Bayesian confidence interval for $\eta ^*_{k,\{j\}}$. Note that the domains are in the original scales of gene expressions for better interpretability

**Fig. 5:**
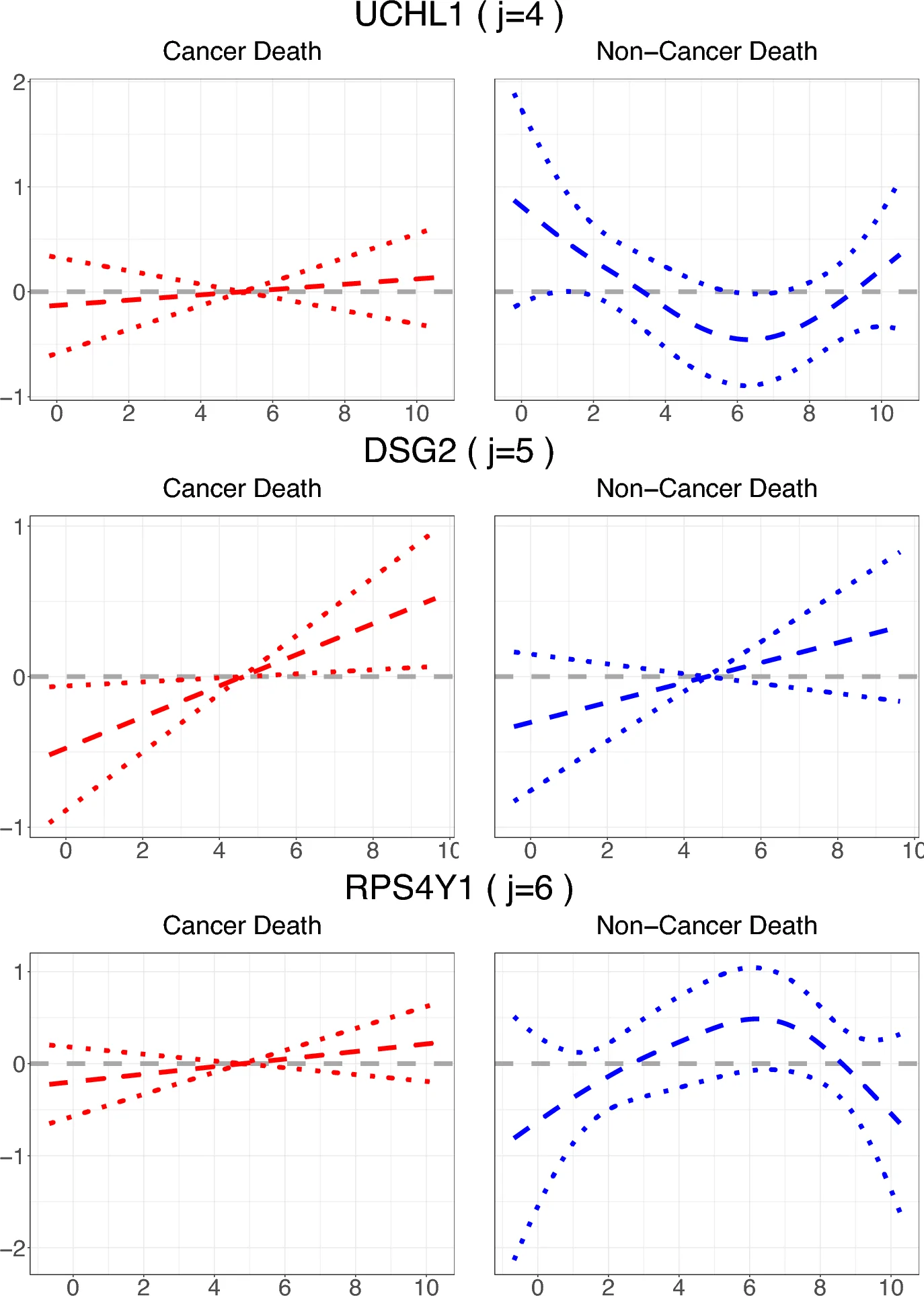
MMRF data analysis results. For each row, the title represents the covariate and the subtitles correspond to the associated risks. For each plot, the dashed line represents $\hat{\eta }_{k,\{j\}}$ and the dotted lines indicate the 95% Bayesian confidence interval for $\eta ^*_{k,\{j\}}$. Note that the domains are in the original scales of gene expressions for better interpretability

In Figs. 4 and 5, we observe that for many genes, the fitted curves $\hat{\eta }_{k,\{j\}}$ for multiple myeloma-related death and non-multiple myeloma-related death exhibit notably different shapes. This suggests the necessity of competing risk modeling for these risk factors. Interestingly, the effect of age differed between the cancer and non-cancer groups. In the non-cancer group, age exhibited a significant linear effect, whereas in the cancer group the estimated effect showed a non-significant nonlinear pattern, decreasing initially and increasing thereafter. This difference is clinically plausible, as age is a well-established risk factor for non-cancer mortality, whereas cancer-related mortality in multiple myeloma is likely driven more strongly by the underlying molecular and genetic characteristics of the disease than by age alone.

Additionally, for four genes, namely RPS6KA6, IGKC, UCHL1, and RPS4Y1, evidence of nonlinearity is observed for at least one of the two risks, namely multiple myeloma-related death or non-multiple myeloma-related death. For example, the fitted effect for multiple myeloma-related death in RPS6KA6 and IGKC, as well as the fitted effects for non-multiple myeloma-related death in RPS6KA6, IGKC, and UCHL1, exhibit nonlinear patterns. Moreover, the corresponding Bayesian confidence intervals exclude zero in certain regions of the covariate domain, suggesting significant nonlinear associations with the corresponding cause-specific hazards. Although the Bayesian confidence intervals for non-cancer death group in RPS4Y1 are relatively wide, its fitted curve still displays nonlinearity. These findings are not reported in the existing literature.

Overall, the analysis demonstrates that for several covariates, at least one cause-specific effect is nonlinear and varies across different causes of death. This highlights that the proposed model fills a gap in the existing literature, where competing risks are often overlooked or covariate effects are assumed to be linear. Furthermore, the estimated effect functions and their Bayesian confidence intervals provide a straightforward interpretation of covariate effects on the corresponding cause-specific hazards, including their direction, significance, and potential nonlinearity.

## Conclusion

In this study, we introduced the smoothing spline competing risk Cox model, which combines the cause-specific Cox model with smoothing spline ANOVA to capture nonparametric covariate effects in competing risks. The proposed model bridges a gap in the literature by estimating nonlinear and distinct cause-specific covariate effects under the proportional cause-specific hazards assumption. For each risk, the estimation procedures are proposed, and the rate of convergence results are established. Our simulations and application to a multiple myeloma dataset demonstrate that the proposed model can estimate distinct effect functions across risk factors, in the presence of nonlinearity.

For future work, we plan to apply the smoothing spline ANOVA framework to various competing risk survival models. The proposed method in this paper combines the classical and fundamental proportional cause-specific hazards model with smoothing spline ANOVA. However, we aim to extend this framework by integrating alternative approaches to proportional cause-specific hazards modeling with smoothing spline ANOVA. For instance, we could combine the proportional subdistribution hazards model (Fine and Gray 1999) with smoothing spline ANOVA or integrate correlated latent cause-specific event times model by copula structure (Lo and Wilke 2010) with smoothing spline ANOVA.

There are a few other interesting directions that merit future research. One is the estimation and asymptotic analysis of the baseline hazard function. Under the proportional hazards framework, the baseline hazard does not enter the estimation of the covariate effects and can subsequently be estimated using a Breslow-type estimator (Breslow 1972). Although we do not investigate its asymptotic properties in the present work, such results are of practical interest and may be established using techniques in Kosorok (2008). The other is to determine whether a covariate effect is linear or nonlinear. As demonstrated in our application, visual inspect assisted by confidence intervals provides a simple way to identify nonlinear effects from linear ones. A more rigorous approach for this purpose would be an extension of the LAND method (Zhang et al. 2011) to the competing risk model setting.

## Supplementary Information

Below is the link to the electronic supplementary material.

Supplementary file 1 (pdf 656 KB)

## Notations

We introduce the notations used throughout our theory. We write $\text {P}_W = \mathbb {P}(W^{-1}(\cdot ))$ for the distribution of *W*, and $\text {P}_{W^n} = \mathbb {P}(\{W_1, \ldots , W_n\}^{-1}(\cdot ))$ for the distribution of $\{W_i\}_{i=1}^n$. We use the operator $\mathbb {E}_W$ to indicate the Lebesgue integral taken with respect to the distribution of either *W* or $\{W_i\}_{i=1}^n$. That is,

$$ \mathbb {E}_W[g(W)] = \int _{\mathcal {W}} g \, d\text {P}_W\text { and }\mathbb {E}_W[g(\{W_i\}_{i=1}^n)] = \int _{\mathcal {W}^n} g \, d\text {P}_{W^n} $$

for any function *g* defined on $\mathcal {W}$ or $\mathcal {W}^n$, where $\mathcal {W} = (0,\infty )\times (0,\tau ]\times \mathcal {X}\times \{1,\ldots ,K\}$. The function *g* may be deterministic or a stochastic process. If *g* is deterministic,

$$ \mathbb {E}_W[g(W)] = \mathbb {E}[g(W)]\text { and } \mathbb {E}_W[g(\{W_i\}_{i=1}^n)] = \mathbb {E}[g(\{W_i\}_{i=1}^n)]. $$

For deterministic sequences $a_n, b_n \in \mathbb {R}$ for $n \in \mathbb {N}$, we set $a_n = \mathcal {O}(b_n)$ ($|a_n| \lesssim |b_n|$) if there exists a constant $\mathcal {C} \in (0,\infty )$ and $N \in \mathbb {N}$ such that $|a_n| \le \mathcal {C} |b_n|$ for all *n ≥ N*. Likewise, $a_n = o(b_n)$ ($|a_n| \ll |b_n|$) is satisfied if for every constant $\epsilon \in (0,\infty )$, there exists $N_\epsilon \in \mathbb {N}$ such that $|a_n| \le \epsilon |b_n|$ for all $n \ge N_\epsilon$. We adopt $a_n \asymp b_n$ to mean that $a_n = \mathcal {O}(b_n)$ and $b_n = \mathcal {O}(a_n)$.

For sequences $X_n, Y_n \in \mathbb {R}$ for $n\in \mathbb {N}$, with at least one being random, we refer to $X_n = \mathcal {O}_{\mathbb {P}}(Y_n)$ ($|X_n| \lesssim |Y_n|$) if there is a constant $\mathcal {C} \in (0,\infty )$ such that $\mathbb {P}(|X_n| \le \mathcal {C} |Y_n|) \rightarrow 1$ as $n \rightarrow \infty$. In addition, $X_n = o_{\mathbb {P}}(Y_n)$ ($|X_n| \ll |Y_n|$) if $\mathbb {P}(|X_n| \le \epsilon |Y_n|) \rightarrow 1$ as $n \rightarrow \infty$ for any constant $\epsilon \in (0,\infty )$.

Here, if a constant does not have a subscript *k*, it is independent of *k*.

Let $\mathcal {U}_1$ and $\mathcal {U}_2$ be vector spaces that are subspaces of a vector space $\mathcal {U}$. We refer to $\mathcal {U} = \mathcal {U}_1 \oplus \mathcal {U}_2$, which can also be expressed as $\mathcal {U}_1 = \mathcal {U} \ominus \mathcal {U}_2$ or $\mathcal {U}_2 = \mathcal {U} \ominus \mathcal {U}_1$, if the followings are satisfied:

1. $$\mathcal {U}_1 \cap \mathcal {U}_2 = \{0\}$$,
2. For every $f \in \mathcal {U}$, there exist unique $f_1 \in \mathcal {U}_1$ and $f_2 \in \mathcal {U}_2$ such that $f = f_1 + f_2$.

For each *j=1,… ,d*, suppose that $\{ \varphi _{j,v} \}_{v \in \mathcal {I}_j}$ forms a basis of Hilbert spaces $\mathcal {V}_j$, where index set $\mathcal {I}_j$ is either finite (when $\mathcal {V}_j$ is finite-dimensional, $|\mathcal {I}_j|\in \mathbb {N}$) or countably infinite (when $\mathcal {V}_j$ is infinite-dimensional, $\mathcal {I}_j=\mathbb {N}$). We denote tensor product space by $\otimes _{j=1}^d \mathcal {V}_j$, which is the completion of $\operatorname {Span}\left\{ \prod _{j=1}^d\varphi _{j,v_j} \right\} _{v_j \in \mathcal {I}_j, j=1,\ldots ,d}$ with respect to some inner product $\langle \cdot ,\cdot \rangle _{\otimes _{j=1}^d \mathcal {V}_j}$ meeting $\langle \prod _{j=1}^d f_j, \prod _{j=1}^d g_j \rangle _{\otimes _{j=1}^d \mathcal {V}_j}=\prod _{j=1}^d\langle f_j, g_j \rangle _{\mathcal {V}_j}$ for all $f_j,g_j \in \mathcal {V}_j$, *j=1,… ,d*, where for each *j=1,… ,d*, $\langle \cdot ,\cdot \rangle _{\mathcal {V}_j}$ is some inner product on $\mathcal {V}_j$.

## Assumptions

In this section, we provide assumptions needed in the rate of convergence for the proposed model. As in Sect. 2.7, we here assume $\mathcal {X}_{[j]} = [0,1]$ for all *j*. Let $\textsf{f}_\mathcal {X}(\cdot )$ be the density function of * X *. For $t \in [0, \tau ]$ and $x \in \mathcal {X}$, let $\textsf{f}_\mathcal {Y|X}(t;x)= \mathbb {E}[Y(t)|X=x] = \mathbb {P}(Y(t)=1|X=x)$ and $\textsf{f}_{\mathcal {Y,X}}(t,x) =\textsf{f}_\mathcal {Y|X}(t;x)\textsf{f}_\mathcal {X}(x)$.

### Assumption B.1

Assume the followings:

1. There exist some constants $\mathcal {C}_\tau \in (0,1)$ and $\mathcal {C}_S \in (0,\infty )$ such that $\mathbb {P}(T\ge \tau )\ge \mathcal {C}_\tau$ and $\inf _{t\in [0,\tau ]}S(\eta ^*_k,t)=S(\eta ^*_k,\tau )>\mathcal {C}_S$ for each *k*.
2. There exists some constant $\mathcal {C}_{R} \in (0,1)$ such that for all *k*, $\mathbb {P}(\tilde{G}=k)\ge \mathcal {C}_{R}$.
3. There exist some constants $0<\mathcal {C}_{h,1}<\mathcal {C}_{h,2}<\infty$ such that for all *k*, $\mathcal {C}_{h,1} \le h_{0k}(t) \le \mathcal {C}_{h,2}$ for all $t\in [0,\tau ]$.
4. There exist some constants $0<\mathcal {C}_{\textsf{f},1}<\mathcal {C}_{\textsf{f},2}<\infty$ such that for all *k*, $\mathcal {C}_{\textsf{f},1}< \textsf{f}_{\mathcal {Y,X}}(t,x)<\mathcal {C}_{\textsf{f},2}$ for all $t \in [0,\tau ]$ and all $x \in \mathcal {X}$.
5. There exists some constant $\mathcal {C}_*\in (0,\infty )$ such that for all *k*, $-\mathcal {C}_*\le \eta ^*_k(x)\le \mathcal {C}_*$ for all $x\in \mathcal {X}$. Furthermore, for each *k*, there exists deterministic big convex neighborhood of $\eta ^*_k$, denoted by $\mathcal {B}_k=\{f\in \mathcal {H}:\mathcal {V}_k(f-\eta ^*_k) \le \rho _k\}$ with some constant $\rho _k \in (0,\infty )$, which does not depend on *n*, such that there exist some constants $0< \mathcal {C}_{k,1}< 1< \mathcal {C}_{k,2} < \infty$ such that for all $\eta \in \mathcal {B}_k$, $\mathcal {C}_{k,1} \exp (\eta ^*_k(x)) \le \exp (\eta (x)) \le \mathcal {C}_{k,2}\exp (\eta ^*_k(x))$ for all $x \in \mathcal {X}$.
6. For each * k *, $\mathcal {V}_k$ is completely continuous with respect to $J_k$ on $\mathcal {H}$, implying that there exists an eigensystem $\{\rho _{k,v}, \psi _{k,v}\}_{v\in \mathbb {N}}$ such that for all $v \in \mathbb {N}$, the eigenfunction $\psi _{k,v} \in \mathcal {H}$ and the eigenvalue $\rho _{k,v} \ge 0$ satisfy $\rho _{k,v} \rightarrow \infty$ as $v \rightarrow \infty$, and $\mathcal {V}_k(\psi _{k,v}, \psi _{k,v'}) = 1\!\!1_{v,v'} \equiv 1\!\!1(v = v')$, while $J_k(\psi _{k,v}, \psi _{k,v'}) = \rho _{k,v} 1\!\!1_{v,v'}$ for all $v, v' \in \mathbb {N}$. Furthermore, for any $f \in \mathcal {H}$, we have $f = \sum _{v\in \mathbb {N}} \mathcal {V}_k(f, \psi _{k,v}) \psi _{k,v}$, since for all $f \in \mathcal {H}$, * J(f) < ∞ *, which is equivalent to $J_k(f)<\infty$.
  There exist some constants $\mathcal {C}_\psi , \mathcal {C}_\rho \in (0,\infty )$ such that for each * k *, $\sup _{v \in \mathbb {N}} \Vert \psi _{k,v}\Vert _{\sup } \le \mathcal {C}_\psi$ and $\rho _{k,v} > \mathcal {C}_\rho v^r$ as $v \rightarrow \infty$, where *r>1*. Note that *r = 2m* when *d = 1*, or when *d > 1* and $\mathcal {H}$ has an additive structure; and *r = 2m - δ * for any $\delta \in (0, 2\,m - 1)$ when *d > 1* and $\mathcal {H}$ contains at least one interaction.
7. For each *k*, there exists some constant $b_k\in [1,2]$ such that $\sum _{v\in \mathbb {N}} \rho _{k,v}^{b_k} \mathcal {V}_k^2(\eta ^*_k,\psi _{k,v}) < \infty$.
8. For each *k*, there exists some constant $\zeta _k\in (0,\infty )$ such that as $n\rightarrow \infty$, $q_k \asymp n^{\zeta _k+2/(rb_k+1)}$, $\lambda _k \rightarrow 0$, and $q_k\lambda _k^{2/r} \rightarrow \infty$.

Let $n(\cdot ) = \sum _{i=1}^n Y_i(\cdot )$. By the strong law of large numbers and (1), which is common in the survival analysis literature, we have $n(\tau )/n \overset{\text {a.s.}}{\longrightarrow }\mathbb {P}(T \ge \tau ) > \mathcal {C}_\tau$ as $n \rightarrow \infty$. Given that * n(· ) * is non-increasing on *[0, τ ]*, there are sufficient number of units at risk for any $t \in [0,\tau ]$. Therefore, for simplicity, we assume $n(\tau ) \ge 1$, which is reasonable based on the assumption above.

Note that (2) indicates that the probability of each risk factor is above a certain threshold. This implies that, when * n * is sufficiently large, for each risk factor, we have a sufficiently large number of subjects with observed risk corresponding to that risk factor.

The uniformly boundedness assumptions in (3) and (4) are borrowed from Du et al. (2010). Specifically, (3) leads to $\mathcal {C}_{h,1} \tau \le \inf _{t \in [0, \tau ]} h_{0k}(t) \cdot \tau \le \int _0^\tau dH_{0k}(t) = H_{0k}(\tau ) \le \sup _{t \in [0, \tau ]} h_{0k}(t) \cdot \tau \le \mathcal {C}_{h,2} \tau$. Observe that (4) is a uniformly boundedness condition used to show that Theorem 1 implies rate of convergence with respect to the $\mathcal {L}_2$ norm.

The set $\mathcal {B}_k$ in (5) is large enough to ensure that all estimators of $\eta ^*_k$ in the technical proofs, including $\hat{\eta }_k$ and $\hat{\eta }^*_k$, are elements of $\mathcal {B}_k$. This is a common assumption in the smoothing spline literature (Du et al. 2010; Gu 2013).

Constructing the eigensystem on $\mathcal {H}$ for each * k *, as described in (6), is a standard approach in the smoothing spline literature (Du et al. 2010; Gu 2013).

The smoothness level of the true function $\eta ^*_k$ is described by the parameter $b_k$. When $b_k=1$, we have $\sum _{v\in \mathbb {N}} \rho _{k,v}^{b_k} \mathcal {V}_k^2(\eta ^*_k,\psi _{k,v}) = J_k(\eta ^*_k)$, ensuring that condition (7) is automatically satisfied by $\eta ^*_k \in \mathcal {H}$.

The order of the number of knots required for efficient approximation, as well as the order of the smoothing parameter in relation to the number of knots, are discussed in (8). Both are common assumptions in the smoothing spline literature (Du et al. 2010; Gu 2013). When $\lambda _k \asymp n^{-r/(rb_k+1)} \rightarrow 0$ as $n\rightarrow \infty$, which guarantees the optimal convergence rate, $q_k \lambda _k^{2/r} \asymp n^{\zeta _k} \rightarrow \infty$ as $n\rightarrow \infty$ is also satisfied, thereby ensuring that (8) holds.
