# Supplementary material for: Competing risk model with a nonparametric form of relative risks
Source: Lifetime Data Anal. 2026 Aug 19;32(3):49. doi: 10.1007/s10985-026-09728-8 (PMC13490231; doi:10.1007/s10985-026-09728-8)
Supplement: Supplementary file 1 [file 10985_2026_9728_MOESM1_ESM.pdf]

Noname manuscript No.
(will be inserted by the editor)

Supplementary Material

for "Competing Risk Model with A Nonparametric Form of Relative Risks"

Received: date / Accepted: date

Abstract In this supplementary material, we provide technical details supporting the theoretical results in Section 2.7 and additional numerical results complementing the studies in Section 3 of the main paper. Section S1 presents the main proof of Theorem 1 and Section S2 provides the supporting technical details for Theorem 1. As in Section 2.7, we here assume $\mathcal{X}_{[j]} = [0, 1]$ for all j. Section S3 provides additional simulation results.

S1 Proof of Theorem 1

Here, we employ quadratic approximation techniques as outlined by Gu (2013) to prove Theorem 1, with proofs closely following those in Du et al. (2010). Without loss of generality, any arguments here are for any k. First, we introduce some notations that are used in the proofs. For $\eta, f, g \in \mathcal{H}$, let $\mathcal{U}(f; \eta, t) = S(f; \eta, t)/S(\eta, t)$, $\mathcal{U}_{(n)}(f; \eta, t) = S_{(n)}(f; \eta, t)/S_{(n)}(\eta, t)$, $\mathcal{V}(f, g; \eta, t) = S(fg; \eta, t)/S(\eta, t) - \mathcal{U}(f; \eta, t)\mathcal{U}(g; \eta, t)$, and

$$\mathcal{V}_{(n)}(f, g; \eta, t) = S_{(n)}(fg; \eta, t)/S_{(n)}(\eta, t) - \mathcal{U}_{(n)}(f; \eta, t)\mathcal{U}_{(n)}(g; \eta, t),$$

where $S_{(n)}(\eta, t) \equiv \sum_{i=1}^{n} Y_i(t) \exp(\eta(X_i))/n$, $S_{(n)}(f; \eta, t) \equiv \sum_{i=1}^{n} Y_i(t) f(X_i) \exp(\eta(X_i))/n$, and $S_{(n)}(fg; \eta, t) \equiv \sum_{i=1}^{n} Y_i(t) f(X_i) g(X_i) \exp(\eta(X_i))/n$ for $f, g, \eta \in \mathcal{H}$ and $t \in [0, \tau]$.

Then, we can express $V_k(\cdot, \cdot)$ as

$$V_k(f, g) = \int_0^\tau \mathcal{V}(f, g; \eta_k^*, t) S(\eta_k^*, t) dH_{0k}(t)$$

for $f, g \in \mathcal{H}$. For $\eta \in \mathcal{H}$, we define a non-negative definite bivariate functional $V_{\eta,k}^{(n)}(\cdot, \cdot)$ on $\mathcal{H}$ as follows. For $f, g \in \mathcal{H}$, let

$$V_{\eta,k}^{(n)}(f, g) = \int_0^\tau \mathcal{V}_{(n)}(f, g; \eta, t) d\overline{N}_k(t).$$

Address(es) of author(s) should be given

Let $\mathcal{V}_{\eta,k}^{(n)}(f) = \mathcal{V}_{\eta,k}^{(n)}(f,f)$ for $\eta, f \in \mathcal{H}$. Define $\mathcal{V}_k^{(n)}(\cdot,\cdot) = \mathcal{V}_{\eta_k^*,k}^{(n)}(\cdot,\cdot)$. For $\eta, f \in \mathcal{H}$, let

$$\mathcal{U}_{\eta,k}^{(n)}(f) = \int_0^\tau \mathfrak{U}_{(n)}(f;\eta,t)d\overline{N}_k(t), \text{ where } \mathcal{U}_k^{(n)}(\cdot) \equiv \mathcal{U}_{\eta_k^*,k}^{(n)}(\cdot).$$

We begin by introducing the remarks and lemmas that are instrumental in our main proof. Initially, we require an order bound for the eigenvalues and λ_k, as described in Remark S1.

Remark S1 Under Assumption B.1, for each k,

$$\sum_{v\in\mathbb{N}} \frac{\lambda_k \rho_{k,v}}{(1+\lambda_k\rho_{k,v})^2}, \sum_{v\in\mathbb{N}} \frac{1}{(1+\lambda_k\rho_{k,v})^2}, \text{ and } \sum_{v\in\mathbb{N}} \frac{1}{1+\lambda_k\rho_{k,v}}$$

are $\mathcal{O}_{\lambda_k}(\lambda_k^{-1/r})$, where $\mathcal{O}_{\lambda_k}(\cdot)$ is $\mathcal{O}(\cdot)$ with respect to $\lambda_k \to 0$.

The proof of Remark S1 is provided in Gu (2013), where the condition $r > 1$ is required. Since $\lambda_k \to 0$ as $n \to \infty$, $\mathcal{O}_{\lambda_k}(\cdot)$ in Remark S1 is replaced with $\mathcal{O}(\cdot)$ as $n \to \infty$.

We demonstrate that $\mathcal{V}_k$ and $\|\cdot\|_{\mathcal{L}_2}^2$ are equivalent in $\mathcal{H}$, as established by Lemma S1.

Lemma S1 *Under Assumption B.1, there exist some constants* $0 < \mathcal{C}_{\mathcal{L}_2,1} < \mathcal{C}_{\mathcal{L}_2,2} < \infty$ *such that for each* k*, for all* $f \in \mathcal{H}$*,* $\mathcal{C}_{\mathcal{L}_2,1}\|f\|_{\mathcal{L}_2}^2 \le \mathcal{V}_k(f) \le \mathcal{C}_{\mathcal{L}_2,2}\|f\|_{\mathcal{L}_2}^2$.

The proof of Lemma S1 is detailed in Section S2. According to Lemma S1, the convergence rate between $\hat{\eta}_k$ and η_k^* with respect to $\|\cdot\|_{\mathcal{L}_2}$ is equivalent to that with respect to $(\mathcal{V}_k + \lambda_k J_k)^{1/2}$ as stated in Theorem 1.

We present a series of important results related to convergence and asymptotics in Lemmas S2 − S4, with detailed proofs provided in Section S2. Additionally, we establish results concerning boundedness and orthogonality in Lemmas S5 and S6, also included in Section S2.

Lemma S2 *Under Assumption B.1, for each* k*, for all* $f, g \in \mathcal{H}$*, as* $n \to \infty$*,*

$$\begin{aligned}\mathcal{V}_k^{(n)}(f,g) &= \mathcal{V}_k(f,g) + \sqrt{(\mathcal{V}_k+\lambda_kJ_k)(f)(\mathcal{V}_k+\lambda_kJ_k)(g)}\,\mathcal{O}_{\mathbb{P}}\left(n^{-1/2}\lambda_k^{-1/r}\right)\\ &= \mathcal{V}_k(f,g) + o_{\mathbb{P}}\left(\sqrt{(\mathcal{V}_k+\lambda_kJ_k)(f)(\mathcal{V}_k+\lambda_kJ_k)(g)}\right).\end{aligned}$$

Lemma S3 *Under Assumption B.1, for each* k*, for all* $f, g \in \mathcal{H}$*, there exists some random variable* $\alpha \in (0,1)$*, such that*

$$\mathcal{U}_{f,k}^{(n)}(f-g) - \mathcal{U}_{g,k}^{(n)}(f-g) = \mathcal{V}_{g+\alpha(f-g),k}^{(n)}(f-g).$$

Lemma S4 *Under Assumption B.1, for each k, let a stochastic process $f_k \in \mathcal{H}$ satisfies $\mathcal{V}_k^{(n)}(f_k - \eta_k^*) = o_{\mathbb{P}}(1)$ as $n \to \infty$. Then for all $h \in \mathcal{H}$, as $n \to \infty$,*

$$\mathcal{U}_{f_k,k}^{(n)}(h) - \mathcal{U}_k^{(n)}(h) = \mathcal{V}_k^{(n)}(f_k - \eta_k^*, h)\,(1 + o_{\mathbb{P}}(1)).$$

Furthermore, if another stochastic process $g_k \in \mathcal{H}$ satisfies $\mathcal{V}_k^{(n)}(g_k - \eta_k^*) = o_{\mathbb{P}}(1)$ as $n \to \infty$. Then for all $h \in \mathcal{H}$, as $n \to \infty$,

$$\mathcal{U}_{f_k,k}^{(n)}(h) - \mathcal{U}_{g_k,k}^{(n)}(h) = \mathcal{V}_k^{(n)}(f_k - g_k, h)\,(1 + o_{\mathbb{P}}(1)).$$

The proof for the main result in Theorem 1 is presented in the following three steps.

Step 1: Rate for the Quadratic Approximation Optimizer

For $\eta, f \in \mathcal{H}$, define

$$\mathcal{S}_{\eta,k}^{(n)}(f) = \int_0^\tau \frac{1}{n}\sum_{i=1}^n \left\{f(X_i) - \mathfrak{U}_{(n)}(f;\eta,t)\right\} dN_{ik}(t),$$

which represents the Fréchet derivative of $-\mathcal{L}_{k(n)}(\cdot)$ at η in the direction of f.

The quadratic approximation optimizer $\tilde{\eta}_k^*$ is defined by

$$\tilde{\eta}_k^* = \operatorname*{argmin}_{f\in\mathcal{H}} -\mathcal{S}_{\eta_k^*,k}^{(n)}(f) + \frac{1}{2}\mathcal{V}_k(f - \eta_k^*) + \frac{\lambda_k}{2}J_k(f), \tag{S1.1}$$

where assume $\tilde{\eta}_k^* \in \mathcal{B}_k$. The loss function in (S1.1) is a quadratic approximation of the loss function in (1) at η_k^*, which can be represented as

$$\sum_{v\in\mathbb{N}}\left\{-\mathcal{V}_k(f,\psi_{k,v})\mathcal{S}_{\eta_k^*,k}^{(n)}(\psi_{k,v}) + \frac{1}{2}\left(\mathcal{V}_k(f,\psi_{k,v}) - \mathcal{V}_k(\eta_k^*,\psi_{k,v})\right)^2 + \frac{\lambda_k}{2}\rho_{k,v}\mathcal{V}_k^2(f,\psi_{k,v})\right\}. \tag{S1.2}$$

Setting the derivative of (S1.2) at $\mathcal{V}_k(f,\psi_{k,v})$ to be 0, one gets $\tilde{\eta}_k^* = \sum_{v\in\mathbb{N}}\mathcal{V}_k(\tilde{\eta}_k^*,\psi_{k,v})\psi_{k,v}$, where

$$\mathcal{V}_k(\tilde{\eta}_k^*,\psi_{k,v}) = \frac{1}{1+\lambda_k\rho_{k,v}}\left\{\mathcal{V}_k(\eta_k^*,\psi_{k,v}) + \mathcal{S}_{\eta_k^*,k}^{(n)}(\psi_{k,v})\right\}.$$

One can represent $\mathcal{S}_{\eta_k^*,k}^{(n)}(\psi_{k,v})$ by

$$\mathcal{S}_{\eta_k^*,k}^{(n)}(\psi_{k,v}) = \frac{1}{n}\sum_{i=1}^n\int_0^\tau \left\{\psi_{k,v}(X_i) - \mathfrak{U}_{(n)}(\psi_{k,v};\eta_k^*,t)\right\} dM_{ik}(t) = \frac{1}{n}\sum_{i=1}^n\int_0^\tau B_{i,k}^{(v)}(t)dM_{ik}(t),$$

where

$$\mathbb{E}\left[\mathcal{S}_{\eta_k^*,k}^{(n)}(\psi_{k,v})\right] = \frac{1}{n}\sum_{i=1}^n\int_0^\tau \mathbb{E}\left[B_{i,k}^{(v)}(t)dM_{ik}(t)\right] = \frac{1}{n}\sum_{i=1}^n\int_0^\tau \mathbb{E}\left[\mathbb{E}\left[B_{i,k}^{(v)}(t)dM_{ik}(t)\middle|\mathcal{F}_{t^-}\right]\right]$$

$$= \frac{1}{n}\sum_{i=1}^{n}\int_0^{\tau}\mathbb{E}\left[B_{i,k}^{(v)}(t)\mathbb{E}\left[dM_{ik}(t)|\mathcal{F}_{t^-}\right]\right] = 0$$

and

$$\mathbb{E}\left[\left\{\mathcal{S}_{\eta_k^*,k}^{(n)}(\psi_{k,v})\right\}^2\right] = \frac{1}{n}\mathbb{E}\left[\frac{1}{n}\sum_{i=1}^{n}\int_0^{\tau}\left\{B_{i,k}^{(v)}(t)\right\}^2 d\langle M_{ik}\rangle(t)\right] \le \frac{2\exp(\mathcal{C}_*)\mathcal{C}_{\psi}^2\mathcal{C}_{h,2}\tau}{n}. \tag{S1.3}$$

In (S1.3), the first equality is derived by

$$\begin{aligned}\mathbb{E}\left[\int_0^{\tau}B_{i,k}^{(v)}(t)dM_{ik}(t)\int_0^{\tau}B_{i',k}^{(v)}(t)dM_{i'k}(t)\right] &= \mathbb{E}\left[\int_0^{\tau}B_{i,k}^{(v)}(t)B_{i',k}^{(v)}(t)d\langle M_{ik}, M_{i'k}\rangle(t)\right]\\ &= \mathbb{E}\left[\int_0^{\tau}\left\{B_{i,k}^{(v)}(t)\right\}^2 d\langle M_{ik}\rangle(t)\right]\mathbb{1}_{i,i'},\end{aligned}$$

which follows from Theorem 2.4.4. in Fleming and Harrington (1991), and the second inequality is derived by

$$\begin{aligned}\frac{1}{n}\sum_{i=1}^{n}\int_0^{\tau}\left\{B_{i,k}^{(v)}(t)\right\}^2 d\langle M_{ik}\rangle(t) &= \int_0^{\tau}\mathcal{V}_{(n)}(\psi_{k,v},\psi_{k,v};\eta_k^*,t)S_{(n)}(\eta_k^*,t)dH_{0k}(t)\\ &\le \left\{\sup_{v,v'\in\mathbb{N},t\in[0,\tau]}\left|\mathcal{V}_{(n)}(\psi_{k,v},\psi_{k,v'};\eta_k^*,t)\right|\right\}\left\{\sup_{t\in[0,\tau]}S_{(n)}(\eta_k^*,t)\right\}H_{0k}(\tau)\\ &\le 2\exp(\mathcal{C}_*)\mathcal{C}_{\psi}^2\mathcal{C}_{h,2}\tau,\end{aligned}$$

where the last inequality follows from Lemma S5.

Simple calculation yields

$$(\mathcal{V}_k + \lambda_k J_k)(\tilde{\eta}_k^* - \eta_k^*) = \sum_{v\in\mathbb{N}}\frac{\left\{\mathcal{S}_{\eta_k^*,k}^{(n)}(\psi_{k,v})\right\}^2 - 2\lambda_k\rho_{k,v}\mathcal{V}_k(\eta_k^*,\psi_{k,v})\mathcal{S}_{\eta_k^*,k}^{(n)}(\psi_{k,v}) + \lambda_k^2\rho_{k,v}^2\mathcal{V}_k^2(\eta_k^*,\psi_{k,v})}{1+\lambda_k\rho_{k,v}}$$

and

$$\begin{aligned}\mathbb{E}\left[(\mathcal{V}_k + \lambda_k J_k)(\tilde{\eta}_k^* - \eta_k^*)\right] &\le \frac{2\exp(\mathcal{C}_*)\mathcal{C}_{\psi}^2\mathcal{C}_{h,2}\tau}{n}\sum_{v\in\mathbb{N}}\frac{1}{1+\lambda_k\rho_{k,v}} + \lambda_k^{b_k}\sum_{v\in\mathbb{N}}\frac{\lambda_k^{2-b_k}\rho_{k,v}^{2-b_k}}{1+\lambda_k\rho_{k,v}}\rho_{k,v}^{b_k}\mathcal{V}_k^2(\eta_k^*,\psi_{k,v})\\ &\le \frac{2\exp(\mathcal{C}_*)\mathcal{C}_{\psi}^2\mathcal{C}_{h,2}\tau}{n}\sum_{v\in\mathbb{N}}\frac{1}{1+\lambda_k\rho_{k,v}} + \lambda_k^{b_k}\sum_{v\in\mathbb{N}}\rho_{k,v}^{b_k}\mathcal{V}_k^2(\eta_k^*,\psi_{k,v})\end{aligned}$$

$$= \mathcal{O}\left(n^{-1}\lambda_k^{-1/r} + \lambda_k^{b_k}\right),$$

directly implying $\sqrt{(\mathcal{V}_k + \lambda_k J_k)\,(\tilde{\eta}_k^* - \eta_k^*)} = \mathcal{O}_{\mathbb{P}}\left(n^{-1/2}\lambda_k^{-1/(2r)} + \lambda_k^{b_k/2}\right)$.

Step 2: Rate for the Global Optimizer

For $\eta, f \in \mathcal{H}$, let $A(\eta, f)$ and $B(\eta, f)$ denote the Fréchet derivatives of the loss functions in (1) and (S1.1), respectively, at η in the direction of f. These derivatives are expressed as

$$A(\eta, f) = -\mathcal{S}_{\eta,k}^{(n)}(f) + \lambda_k J_k(\eta, f), \tag{S1.4}$$

$$B(\eta, f) = -\mathcal{S}_{\eta_k^*,k}^{(n)}(f) + \mathcal{V}_k(\eta - \eta_k^*, f) + \lambda_k J_k(\eta, f), \tag{S1.5}$$

where, by definition, $A(\hat{\eta}_k^*, f) = 0$ and $B(\tilde{\eta}_k^*, f) = 0$ for any $f \in \mathcal{H}$. Set $\eta = \hat{\eta}_k^*$ and $f = \hat{\eta}_k^* - \tilde{\eta}_k^*$ in (S1.4) and set $\eta = \tilde{\eta}_k^*$ and $f = \hat{\eta}_k^* - \tilde{\eta}_k^*$ in (S1.5). Subtracting the resulting equations gives

$$\begin{aligned}\mathcal{U}_{\hat{\eta}_k^*,k}^{(n)}(\hat{\eta}_k^* - \tilde{\eta}_k^*) - \mathcal{U}_{\tilde{\eta}_k^*,k}^{(n)}(\hat{\eta}_k^* - \tilde{\eta}_k^*) + \lambda_k J_k(\hat{\eta}_k^* - \tilde{\eta}_k^*) \\ = \mathcal{V}_k(\tilde{\eta}_k^* - \eta_k^*, \hat{\eta}_k^* - \tilde{\eta}_k^*) + \mathcal{U}_k^{(n)}(\hat{\eta}_k^* - \tilde{\eta}_k^*) - \mathcal{U}_{\eta_k^*,k}^{(n)}(\hat{\eta}_k^* - \tilde{\eta}_k^*).\end{aligned} \tag{S1.6}$$

By Lemma S3, for some random variable $\alpha_1 \in (0,1)$, we have

$$\begin{aligned}0 \leq\ & \mathcal{U}_{\hat{\eta}_k^*,k}^{(n)}(\hat{\eta}_k^* - \tilde{\eta}_k^*) - \mathcal{U}_{\tilde{\eta}_k^*,k}^{(n)}(\hat{\eta}_k^* - \tilde{\eta}_k^*) + \lambda_k J_k(\hat{\eta}_k^* - \tilde{\eta}_k^*) \\ &= \mathcal{V}_{\alpha_1\hat{\eta}_k^* + (1-\alpha_1)\tilde{\eta}_k^*,k}^{(n)}(\hat{\eta}_k^* - \tilde{\eta}_k^*) + \lambda_k J_k(\hat{\eta}_k^* - \tilde{\eta}_k^*) \\ &\geq \mathcal{C}_{\mathcal{B}_k}(\mathcal{V}_k^{(n)} + \lambda_k J_k)(\hat{\eta}_k^* - \tilde{\eta}_k^*) \\ &= \mathcal{C}_{\mathcal{B}_k}(\mathcal{V}_k + \lambda_k J_k)(\hat{\eta}_k^* - \tilde{\eta}_k^*) + o_{\mathbb{P}}\left((\mathcal{V}_k + \lambda_k J_k)(\hat{\eta}_k^* - \tilde{\eta}_k^*)\right),\end{aligned} \tag{S1.7}$$

where $\alpha_1\hat{\eta}_k^* + (1-\alpha_1)\tilde{\eta}_k^* \in \mathcal{B}_k$, and considering the boundedness of η_k^* and the closeness of $\exp(\eta)$ to $\exp(\eta_k^*)$ for $\eta \in \mathcal{B}_k$, as described in (6) of Assumption B.1, we assume, without loss of generality, that for some constant $\mathcal{C}_{\mathcal{B}_k} \in (0,1)$, the inequality $\mathcal{V}_{\eta,k}^{(n)}(f) \geq \mathcal{C}_{\mathcal{B}_k}\mathcal{V}_k^{(n)}(f)$ holds for all $\eta \in \mathcal{B}_k$ and $f \in \mathcal{H}$.

By Lemma S2, we have $\mathcal{V}_k^{(n)}(\tilde{\eta}_k^* - \eta_k^*) = \mathcal{V}_k(\tilde{\eta}_k^* - \eta_k^*) + o_{\mathbb{P}}\left((\mathcal{V}_k + \lambda_k J_k)(\tilde{\eta}_k^* - \eta_k^*)\right) = o_{\mathbb{P}}(1)$. So, by Lemmas S4 and S2, we get

$$\begin{aligned}0 \leq\ & \mathcal{V}_k(\tilde{\eta}_k^* - \eta_k^*, \hat{\eta}_k^* - \tilde{\eta}_k^*) + \mathcal{U}_k^{(n)}(\hat{\eta}_k^* - \tilde{\eta}_k^*) - \mathcal{U}_{\tilde{\eta}_k^*,k}^{(n)}(\hat{\eta}_k^* - \tilde{\eta}_k^*) \\ &= \mathcal{V}_k(\tilde{\eta}_k^* - \eta_k^*, \hat{\eta}_k^* - \tilde{\eta}_k^*) - \mathcal{V}_k^{(n)}(\tilde{\eta}_k^* - \eta_k^*, \hat{\eta}_k^* - \tilde{\eta}_k^*)\,(1 + o_{\mathbb{P}}(1)) \\ &= o_{\mathbb{P}}\Big(\mathcal{V}_k(\tilde{\eta}_k^* - \eta_k^*, \hat{\eta}_k^* - \tilde{\eta}_k^*)\Big) + o_{\mathbb{P}}\left(\sqrt{(\mathcal{V}_k + \lambda_k J_k)(\tilde{\eta}_k^* - \eta_k^*)(\mathcal{V}_k + \lambda_k J_k)(\hat{\eta}_k^* - \tilde{\eta}_k^*)}\right) \\ &= o_{\mathbb{P}}\left(\sqrt{(\mathcal{V}_k + \lambda_k J_k)(\tilde{\eta}_k^* - \eta_k^*)(\mathcal{V}_k + \lambda_k J_k)(\hat{\eta}_k^* - \tilde{\eta}_k^*)}\right),\end{aligned} \tag{S1.8}$$

where the last equality is by $|\mathcal{V}_k(\tilde{\eta}_k^* - \eta_k^*, \hat{\eta}_k^* - \tilde{\eta}_k^*)| \leq \sqrt{\mathcal{V}_k(\tilde{\eta}_k^* - \eta_k^*)\mathcal{V}_k(\hat{\eta}_k^* - \tilde{\eta}_k^*)}$.

Combining (S1.6), (S1.7), and (S1.8), we have

$$(\mathcal{V}_k + \lambda_k J_k)(\hat{\eta}_k^* - \tilde{\eta}_k^*) = o_{\mathbb{P}}\left(\sqrt{(\mathcal{V}_k + \lambda_k J_k)(\tilde{\eta}_k^* - \eta_k^*)(\mathcal{V}_k + \lambda_k J_k)(\hat{\eta}_k^* - \tilde{\eta}_k^*)}\right),$$

$$\sqrt{(\mathcal{V}_k + \lambda_k J_k)(\hat{\eta}_k^* - \tilde{\eta}_k^*)} = o_{\mathbb{P}}\left(\sqrt{(\mathcal{V}_k + \lambda_k J_k)(\tilde{\eta}_k^* - \eta_k^*)}\right) = o_{\mathbb{P}}\left(n^{-1/2}\lambda_k^{-1/(2r)} + \lambda_k^{b_k/2}\right),$$

where $\sqrt{(\mathcal{V}_k + \lambda_k J_k)(\hat{\eta}_k^* - \eta_k^*)} \leq \sqrt{(\mathcal{V}_k + \lambda_k J_k)(\hat{\eta}_k^* - \tilde{\eta}_k^*)} + \sqrt{(\mathcal{V}_k + \lambda_k J_k)(\tilde{\eta}_k^* - \eta_k^*)}$ leads to $\sqrt{(\mathcal{V}_k + \lambda_k J_k)(\hat{\eta}_k^* - \eta_k^*)} = \mathcal{O}_{\mathbb{P}}\left(n^{-1/2}\lambda_k^{-1/(2r)} + \lambda_k^{b_k/2}\right)$.

Step 3: Rate for the Efficient Approximation Optimizer

Define $\mathcal{V}_k^{(q_k)}$ analogously to $\mathcal{V}_k^{(n)}$, with the distinction that $\mathcal{V}_k^{(q_k)}$ incorporates only q_k subjects corresponding to $\{X_{\ell(k)}\}_{\ell=1}^{q_k}$ in its calculation, specifically for both counting process and predictable processes. Denote $\mathcal{H}^{(k)\perp} = \{f \in \mathcal{H} : \langle f, g\rangle_{\mathcal{H},k} = 0 \text{ for all } g \in \mathcal{H}^{(k)}\}$ by the orthogonal complement of $\mathcal{H}^{(k)}$ in $\mathcal{H}$ with respect to $\langle\cdot,\cdot\rangle_{\mathcal{H},k} = \langle\cdot,\cdot\rangle_{\mathcal{N}_J} + J_k(\cdot,\cdot)$, which is an inner product on $\mathcal{H}$. Similarly, denote $\mathcal{H}_J^{(k)\perp} = \{f \in \mathcal{H}_J : J_k(f,g) = 0 \text{ for all } g \in \mathcal{H}_J^{(k)}\}$ by the orthogonal complement of $\mathcal{H}_J^{(k)}$ in $\mathcal{H}_J$ with respect to $J_k(\cdot,\cdot)$.

One can see that

$$\begin{aligned}\mathcal{H}^{(k)\perp} &= \mathcal{H} \ominus \mathcal{H}^{(k)} \\ &= \mathcal{H}_J^{(k)\perp} = \mathcal{H}_J \ominus \mathcal{H}_J^{(k)}\end{aligned}$$

and any $f \in \mathcal{H} \ominus \mathcal{H}^{(k)}$ satisfies $f(X_{\ell(k)}) = J_k\left(f, \mathcal{K}_{J_k}(X_{\ell(k)},\cdot)\right) = 0$ for $\ell = 1,\ldots,q_k$. Considering this, for any $f \in \mathcal{H} \ominus \mathcal{H}^{(k)}$, a modification of Lemma S2 with respect to $\mathcal{V}_k^{(q_k)}$ leads to

$$\begin{aligned}\mathcal{V}_k(f) &= \left|\mathcal{V}_k^{(q_k)}(f) - \mathcal{V}_k(f)\right| = (\mathcal{V}_k + \lambda_k J_k)(f)\mathcal{O}_{\mathbb{P}}\left(q_k^{-1/2}\lambda_k^{-1/r}\right) \\ &= o_{\mathbb{P}}\left((\mathcal{V}_k + \lambda_k J_k)(f)\right) = o_{\mathbb{P}}\left(\lambda_k J_k(f)\right).\end{aligned} \tag{S1.9}$$

Let $\eta_k = \mathrm{Proj}(\hat{\eta}_k^* \mid \mathcal{H}^{(k)}) = \mathrm{arginf}_{\eta \in \mathcal{H}^{(k)}} \langle \hat{\eta}_k^* - \eta, \hat{\eta}_k^* - \eta\rangle_{\mathcal{H},k}$ denote the projection of $\hat{\eta}_k^*$ onto $\mathcal{H}^{(k)}$ with respect to $\langle\cdot,\cdot\rangle_{\mathcal{H},k}$, with the assumption that $\eta_k \in \mathcal{B}_k$. One can see that $\hat{\eta}_k^* - \eta_k \in \mathcal{H} \ominus \mathcal{H}^{(k)}$. Set $\eta = \hat{\eta}_k^*$ and $f = \hat{\eta}_k^* - \eta_k$ in (S1.4). Considering $J_k(\eta_k, \hat{\eta}_k^* - \eta_k) = 0$ by Lemma S6, we have

$$\begin{aligned}\lambda_k J_k(\hat{\eta}_k^* - \eta_k) &= \mathcal{S}_{\eta_k^*,k}^{(n)}(\hat{\eta}_k^* - \eta_k) \\ &\quad + \mathcal{U}_k^{(n)}(\hat{\eta}_k^* - \eta_k) - \mathcal{U}_{\hat{\eta}_k^*,k}^{(n)}(\hat{\eta}_k^* - \eta_k).\end{aligned} \tag{S1.10}$$

The order of the first term on the right-hand side of (S1.10) can be calculated as

$$\begin{aligned}\left|\mathcal{S}^{(n)}_{\eta_k^*,k}(\hat{\eta}_k^*-\eta_k)\right| &= \left|\sum_{v\in\mathbb{N}}\left(\mathcal{V}_k(\hat{\eta}_k^*,\psi_{k,v})-\mathcal{V}_k(\eta_k,\psi_{k,v})\right)\mathcal{S}^{(n)}_{\eta_k^*,k}(\psi_{k,v})\right| \qquad \text{(S1.11)}\\ &\le \left\{\sum_{v\in\mathbb{N}}(1+\lambda_k\rho_{k,v})\left(\mathcal{V}_k(\hat{\eta}_k^*,\psi_{k,v})-\mathcal{V}_k(\eta_k,\psi_{k,v})\right)^2\right\}^{1/2}\\ &\times\left\{\sum_{v\in\mathbb{N}}\frac{1}{1+\lambda_k\rho_{k,v}}\left\{\mathcal{S}^{(n)}_{\eta_k^*,k}(\psi_{k,v})\right\}^2\right\}^{1/2}\\ &= \sqrt{(\mathcal{V}_k+\lambda_kJ_k)(\hat{\eta}_k^*-\eta_k)}\,\mathcal{O}_{\mathbb{P}}\left(n^{-1/2}\lambda_k^{-1/(2r)}\right)\\ &= \sqrt{\lambda_kJ_k(\hat{\eta}_k^*-\eta_k)}\,\mathcal{O}_{\mathbb{P}}\left(n^{-1/2}\lambda_k^{-1/(2r)}+\lambda_k^{b_k/2}\right)(1+o_{\mathbb{P}}(1)),\end{aligned}$$

where (S1.9) is used, and the following is utilized:

$$\mathbb{E}\left[\sum_{v\in\mathbb{N}}\frac{1}{1+\lambda_k\rho_{k,v}}\left\{\mathcal{S}^{(n)}_{\eta_k^*,k}(\psi_{k,v})\right\}^2\right]\le\frac{2\exp(\mathcal{C}_*)\mathcal{C}_\psi^2\mathcal{C}_{h,2}\tau}{n}\sum_{v\in\mathbb{N}}\frac{1}{1+\lambda_k\rho_{k,v}}=\mathcal{O}\left(n^{-1}\lambda_k^{-1/r}\right).$$

By Lemma S2, we have $\mathcal{V}_k^{(n)}(\hat{\eta}_k^*-\eta_k^*)=\mathcal{V}_k(\hat{\eta}_k^*-\eta_k^*)+o_{\mathbb{P}}\left((\mathcal{V}_k+\lambda_kJ_k)(\hat{\eta}_k^*-\eta_k^*)\right)=o_{\mathbb{P}}(1)$. So, by (S1.9) and Lemmas S4 and S2, the order of the remaining terms on the right-hand side of (S1.10) can be calculated as

$$\begin{aligned}&\left|\mathcal{U}_k^{(n)}(\hat{\eta}_k^*-\eta_k)-\mathcal{U}^{(n)}_{\hat{\eta}_k^*,k}(\hat{\eta}_k^*-\eta_k)\right|=\left|\mathcal{V}_k^{(n)}(\hat{\eta}_k^*-\eta_k^*,\hat{\eta}_k^*-\eta_k)\left(1+o_{\mathbb{P}}(1)\right)\right| \qquad \text{(S1.12)}\\ &=\left|\left\{\mathcal{V}_k(\hat{\eta}_k^*-\eta_k^*,\hat{\eta}_k^*-\eta_k)+o_{\mathbb{P}}\left(\sqrt{(\mathcal{V}_k+\lambda_kJ_k)(\hat{\eta}_k^*-\eta_k^*)\lambda_kJ_k(\hat{\eta}_k^*-\eta_k)}\right)\right\}(1+o_{\mathbb{P}}(1))\right|\\ &=o_{\mathbb{P}}\left(\sqrt{(\mathcal{V}_k+\lambda_kJ_k)(\hat{\eta}_k^*-\eta_k^*)\lambda_kJ_k(\hat{\eta}_k^*-\eta_k)}\right)=\sqrt{\lambda_kJ_k(\hat{\eta}_k^*-\eta_k)}\,o_{\mathbb{P}}\left(n^{-1/2}\lambda_k^{-1/(2r)}+\lambda_k^{b_k/2}\right),\end{aligned}$$

where

$$\begin{aligned}|\mathcal{V}_k(\hat{\eta}_k^*-\eta_k^*,\hat{\eta}_k^*-\eta_k)|&\le\sqrt{(\mathcal{V}_k+\lambda_kJ_k)(\hat{\eta}_k^*-\eta_k^*)\mathcal{V}_k(\hat{\eta}_k^*-\eta_k)}\\ &=o_{\mathbb{P}}\left(\sqrt{(\mathcal{V}_k+\lambda_kJ_k)(\hat{\eta}_k^*-\eta_k^*)\lambda_kJ_k(\hat{\eta}_k^*-\eta_k)}\right).\end{aligned}$$

Combining (S1.10), (S1.11), and (S1.12) and considering (S1.9), we get

$$\begin{aligned}\lambda_kJ_k(\hat{\eta}_k^*-\eta_k)&=\sqrt{\lambda_kJ_k(\hat{\eta}_k^*-\eta_k)}\,\mathcal{O}_{\mathbb{P}}\left(n^{-1/2}\lambda_k^{-1/(2r)}+\lambda_k^{b_k/2}\right),\\ (\mathcal{V}_k+\lambda_kJ_k)(\hat{\eta}_k^*-\eta_k)&=\mathcal{O}_{\mathbb{P}}\left(\left\{n^{-1/2}\lambda_k^{-1/(2r)}+\lambda_k^{b_k/2}\right\}^2\right),\end{aligned}$$

where $\sqrt{(\mathcal{V}_k+\lambda_k J_k)(\eta_k-\eta_k^*)} \le \sqrt{(\mathcal{V}_k+\lambda_k J_k)(\eta_k-\hat{\eta}_k^*)}+\sqrt{(\mathcal{V}_k+\lambda_k J_k)(\hat{\eta}_k^*-\eta_k^*)}$ leads to $\sqrt{(\mathcal{V}_k+\lambda_k J_k)(\eta_k-\eta_k^*)} = \mathcal{O}_{\mathbb{P}}\left(n^{-1/2}\lambda_k^{-1/(2r)}+\lambda_k^{b_k/2}\right)$.

As $J_k(\eta_k,\hat{\eta}_k^*-\eta_k)=J_k(\hat{\eta}_k,\hat{\eta}_k^*-\eta_k)=0$ by Lemma S6, we get

$$\begin{aligned} J_k(\hat{\eta}_k^*,\hat{\eta}_k^*-\hat{\eta}_k) &= J_k(\hat{\eta}_k^*-\eta_k+\eta_k,\hat{\eta}_k^*-\eta_k+\eta_k-\hat{\eta}_k) \\ &= J_k(\hat{\eta}_k^*-\eta_k)+J_k(\eta_k,\eta_k-\hat{\eta}_k). \end{aligned} \tag{S1.13}$$

By definition, $A(\hat{\eta}_k,f)=0$ for any $f\in\mathcal{H}^{(k)}$. Set $\eta=\hat{\eta}_k$ and $f=\hat{\eta}_k-\eta_k$ in (S1.4) and set $\eta=\hat{\eta}_k^*$ and $f=\hat{\eta}_k^*-\hat{\eta}_k$ in (S1.4). Using (S1.13) and (S1.10), summing the resulting equations gives

$$\begin{aligned} &\mathcal{U}^{(n)}_{\hat{\eta}_k,k}(\hat{\eta}_k-\eta_k)-\mathcal{U}^{(n)}_{\hat{\eta}_k^*,k}(\hat{\eta}_k-\eta_k)+\lambda_k J_k(\hat{\eta}_k-\eta_k) \\ &\quad=\mathcal{S}^{(n)}_{\eta_k^*,k}(\hat{\eta}_k^*-\eta_k)-\lambda_k J_k(\hat{\eta}_k^*-\eta_k)+\mathcal{U}^{(n)}_k(\hat{\eta}_k^*-\eta_k)-\mathcal{U}^{(n)}_{\hat{\eta}_k^*,k}(\hat{\eta}_k^*-\eta_k)=0, \end{aligned}$$

which can be represented as

$$\mathcal{U}^{(n)}_{\hat{\eta}_k,k}(\hat{\eta}_k-\eta_k)-\mathcal{U}^{(n)}_{\eta_k,k}(\hat{\eta}_k-\eta_k)+\lambda_k J_k(\hat{\eta}_k-\eta_k)=\mathcal{U}^{(n)}_{\hat{\eta}_k^*,k}(\hat{\eta}_k-\eta_k)-\mathcal{U}^{(n)}_{\eta_k,k}(\hat{\eta}_k-\eta_k). \tag{S1.14}$$

By Lemma S3, for some random variable $\alpha_2\in(0,1)$, we have

$$\begin{aligned} 0\le\ &\mathcal{U}^{(n)}_{\hat{\eta}_k,k}(\hat{\eta}_k-\eta_k)-\mathcal{U}^{(n)}_{\eta_k,k}(\hat{\eta}_k-\eta_k)+\lambda_k J_k(\hat{\eta}_k-\eta_k) \\ &=\mathcal{V}^{(n)}_{\alpha_2\hat{\eta}_k+(1-\alpha_2)\eta_k,k}(\hat{\eta}_k-\eta_k)+\lambda_k J_k(\hat{\eta}_k-\eta_k) \\ &\ge\mathcal{C}_{\mathcal{B}_k}(\mathcal{V}^{(n)}_k+\lambda_k J_k)(\hat{\eta}_k-\eta_k) \\ &=\mathcal{C}_{\mathcal{B}_k}(\mathcal{V}_k+\lambda_k J_k)(\hat{\eta}_k-\eta_k)+o_{\mathbb{P}}\left((\mathcal{V}_k+\lambda_k J_k)(\hat{\eta}_k-\eta_k)\right), \end{aligned} \tag{S1.15}$$

where $\alpha_2\hat{\eta}_k+(1-\alpha_2)\eta_k\in\mathcal{B}_k$.

We have $\mathcal{V}^{(n)}_k(\hat{\eta}_k^*-\eta_k^*)=o_{\mathbb{P}}(1)$ and by Lemma S2, we have $\mathcal{V}^{(n)}_k(\eta_k-\eta_k^*)=\mathcal{V}_k(\eta_k-\eta_k^*)+o_{\mathbb{P}}\left((\mathcal{V}_k+\lambda_k J_k)(\eta_k-\eta_k^*)\right)=o_{\mathbb{P}}(1)$. So, by Lemmas S4 and S2, we get

$$\begin{aligned} 0\le\ &\mathcal{U}^{(n)}_{\hat{\eta}_k^*,k}(\hat{\eta}_k-\eta_k)-\mathcal{U}^{(n)}_{\eta_k,k}(\hat{\eta}_k-\eta_k) \\ &=\mathcal{V}^{(n)}_k(\hat{\eta}_k^*-\eta_k,\hat{\eta}_k-\eta_k)\left(1+o_{\mathbb{P}}(1)\right) \\ &=\mathcal{V}_k(\hat{\eta}_k^*-\eta_k,\hat{\eta}_k-\eta_k)+o_{\mathbb{P}}\left(\mathcal{V}_k(\hat{\eta}_k^*-\eta_k,\hat{\eta}_k-\eta_k)\right) \\ &+o_{\mathbb{P}}\left(\sqrt{(\mathcal{V}_k+\lambda_k J_k)(\hat{\eta}_k^*-\eta_k)(\mathcal{V}_k+\lambda_k J_k)(\hat{\eta}_k-\eta_k)}\right) \\ &=\mathcal{O}_{\mathbb{P}}\left(\sqrt{(\mathcal{V}_k+\lambda_k J_k)(\hat{\eta}_k^*-\eta_k)(\mathcal{V}_k+\lambda_k J_k)(\hat{\eta}_k-\eta_k)}\right), \end{aligned} \tag{S1.16}$$

where $|\mathcal{V}_k(\hat{\eta}_k^*-\eta_k,\hat{\eta}_k-\eta_k)|\le\sqrt{(\mathcal{V}_k+\lambda_k J_k)(\hat{\eta}_k^*-\eta_k)(\mathcal{V}_k+\lambda_k J_k)(\hat{\eta}_k-\eta_k)}$.

Combining (S1.14), (S1.15), and (S1.16), we have

$$(\mathcal{V}_k+\lambda_k J_k)(\hat{\eta}_k-\eta_k)=\mathcal{O}_{\mathbb{P}}\left(\sqrt{(\mathcal{V}_k+\lambda_k J_k)(\hat{\eta}_k^*-\eta_k)(\mathcal{V}_k+\lambda_k J_k)(\hat{\eta}_k-\eta_k)}\right),$$

$$\sqrt{(\mathcal{V}_k+\lambda_k J_k)(\hat{\eta}_k-\eta_k)} = \mathcal{O}_{\mathbb{P}}\left(\sqrt{(\mathcal{V}_k+\lambda_k J_k)(\hat{\eta}^*_k-\eta_k)}\right) = \mathcal{O}_{\mathbb{P}}\left(n^{-1/2}\lambda_k^{-1/(2r)}+\lambda_k^{b_k/2}\right),$$

where $\sqrt{(\mathcal{V}_k+\lambda_k J_k)(\hat{\eta}_k-\eta^*_k)} \le \sqrt{(\mathcal{V}_k+\lambda_k J_k)(\hat{\eta}_k-\eta_k)}+\sqrt{(\mathcal{V}_k+\lambda_k J_k)(\eta_k-\eta^*_k)}$ leads to $\sqrt{(\mathcal{V}_k+\lambda_k J_k)(\hat{\eta}_k-\eta^*_k)} = \mathcal{O}_{\mathbb{P}}\left(n^{-1/2}\lambda_k^{-1/(2r)}+\lambda_k^{b_k/2}\right)$.

Note that $n^{-1/2}\lambda_k^{-1/(2r)} \ll n^{-1/4}\lambda_k^{-1/(2r)} \ll q_k^{-1/4}\lambda_k^{-1/(2r)} = o(1)$ implies

$$\mathcal{O}_{\mathbb{P}}\left(n^{-1/2}\lambda_k^{-1/(2r)}+\lambda_k^{b_k/2}\right) = o_{\mathbb{P}}(1),$$

which completes the proof. ■

S2 Technical Details for Theorem 1

Proof of Lemma S1. Here, we consider $t \in [0,\tau]$ and $f \in \mathcal{H}$, where f may represent either a deterministic function or a stochastic process. Let $\mathsf{f}_k(t,x) \equiv \exp(\eta^*_k(x))\mathsf{f}_{\mathcal{Y},\mathcal{X}}(t,x)/S(\eta^*_k,t)$. Then, we have

$$\begin{aligned}\int_{\mathcal{X}} \mathsf{f}_k(t,x)dx &= \frac{\int_{\mathcal{X}} \mathbb{E}[Y(t)|X=x]\exp(\eta^*_k(x))\mathsf{f}_{\mathcal{X}}(x)dx}{S(\eta^*_k,t)} \\ &= \frac{\mathbb{E}\left[\mathbb{E}\left[Y(t)\exp(\eta^*_k(X))|X\right]\right]}{S(\eta^*_k,t)} = \frac{S(\eta^*_k,t)}{S(\eta^*_k,t)} = 1\end{aligned}$$

and

$$\begin{aligned}\mu_k(f)(t) \equiv \frac{S(f;\eta^*_k,t)}{S(\eta^*_k,t)} &= \frac{\mathbb{E}_W\left[\mathbb{E}_W\left[Y(t)|X\right]f(X)\exp(\eta^*_k(X))\right]}{S(\eta^*_k,t)} \\ &= \frac{\mathbb{E}_W\left[\mathbb{E}\left[Y(t)|X\right]f(X)\exp(\eta^*_k(X))\right]}{S(\eta^*_k,t)} \\ &= \frac{\int_{\mathcal{X}} \mathsf{f}_{\mathcal{Y}|\mathcal{X}}(t;x)f(x)\exp(\eta^*_k(x))\mathsf{f}_{\mathcal{X}}(x)dx}{S(\eta^*_k,t)} = \int_{\mathcal{X}} f(x)\mathsf{f}_k(t,x)dx.\end{aligned}$$

Using above, we have

$$\begin{aligned}\mathcal{V}_k(f) &= \int_0^\tau \left\{\int_{\mathcal{X}} \Big(f(x)-\mu_k(f)(t)\Big)^2 \mathsf{f}_k(t,x)dx\right\} S(\eta^*_k,t)dH_{0k}(t) \qquad \text{(S2.17)} \\ &= \int_0^\tau \left\{\int_{\mathcal{X}} \Big(f(x)-\mu_k(f)(t)\Big)^2 \exp(\eta^*_k(x))\mathsf{f}_{\mathcal{Y},\mathcal{X}}(t,x)dx\right\} dH_{0k}(t) \\ &\in \Bigg[\exp(-\mathcal{C}_*)\mathcal{C}_{\mathsf{f},1}\int_0^\tau \left\{\int_{\mathcal{X}} \Big(f(x)-\mu_k(f)(t)\Big)^2 dx\right\} dH_{0k}(t), \\ &\qquad \exp(\mathcal{C}_*)\mathcal{C}_{\mathsf{f},2}\int_0^\tau \left\{\int_{\mathcal{X}} \Big(f(x)-\mu_k(f)(t)\Big)^2 dx\right\} dH_{0k}(t)\Bigg].\end{aligned}$$

Let $\mathfrak{m}(\cdot)$ be the Lebesgue measure on $\mathcal{X}$, Then,

$$\int_0^\tau \left\{\int_{\mathcal{X}} \Big(f(x) - \mu_k(f)(t)\Big)^2 dx\right\} dH_{0k}(t) = H_{0k}(\tau)\|f\|_{\mathcal{L}_2}^2 + \int_0^\tau \Big(\mu_k(f)(t)\Big)^2 dH_{0k}(t),$$

where we used the fact that $\prod_{j=1}^d \mathcal{A}_{[j]}f = \int_{\mathcal{X}} f(x)dx = 0$ due to $f \in \mathcal{H}$ and $\mathfrak{m}(\mathcal{X}) = \mathfrak{m}([0,1]^d) = 1$. By the Cauchy-Schwarz inequality, we have

$$0 \le \Big(\mu_k(f)(t)\Big)^2 \le \int_{\mathcal{X}} (f(x))^2\, dx \int_{\mathcal{X}} (\mathsf{f}_k(t,x))^2\, dx = \|f\|_{\mathcal{L}_2}^2 \int_{\mathcal{X}} (\mathsf{f}_k(t,x))^2\, dx,$$

which leads to

$$\begin{aligned}\int_0^\tau \Big(\mu_k(f)(t)\Big)^2 dH_{0k}(t) &\le \|f\|_{\mathcal{L}_2}^2 \int_0^\tau \left\{\int_{\mathcal{X}} (\mathsf{f}_k(t,x))^2\, dx\right\} dH_{0k}(t)\\ &\le \frac{\exp(2\mathcal{C}_*)\mathcal{C}_{\mathsf{f},2}^2\mathcal{C}_{h,2}}{\mathcal{C}_S^2}\tau\|f\|_{\mathcal{L}_2}^2 = \tilde{\mathcal{C}}\tau\|f\|_{\mathcal{L}_2}^2\end{aligned}$$

and

$$\mathcal{C}_{h,1}\tau\|f\|_{\mathcal{L}_2}^2 \le H_{0k}(\tau)\|f\|_{\mathcal{L}_2}^2 \le \int_0^\tau \left\{\int_{\mathcal{X}} \Big(f(x) - \mu_k(f)(t)\Big)^2 dx\right\} dH_{0k}(t) \le (\mathcal{C}_{h,2} + \tilde{\mathcal{C}})\tau\|f\|_{\mathcal{L}_2}^2. \tag{S2.18}$$

Using (S2.17) and (S2.18), we have

$$\mathcal{C}_{\mathcal{L}_2,1}\|f\|_{\mathcal{L}_2}^2 \le \mathcal{V}_k(f) \le \mathcal{C}_{\mathcal{L}_2,2}\|f\|_{\mathcal{L}_2}^2,$$

where $\mathcal{C}_{\mathcal{L}_2,1} = \exp(-\mathcal{C}_*)\mathcal{C}_{\mathsf{f},1}\mathcal{C}_{h,1}\tau$ and $\mathcal{C}_{\mathcal{L}_2,2} = \exp(\mathcal{C}_*)\mathcal{C}_{\mathsf{f},2}(\mathcal{C}_{h,2} + \tilde{\mathcal{C}})\tau$. ■

Lemma S5 *Under Assumption B.1, for each k, the following results on boundedness hold:*

$$\sup_{t\in[0,\tau]} S(\eta_k^*, t) \le \exp(\mathcal{C}_*), \quad \sup_{t\in[0,\tau]} S_{(n)}(\eta_k^*, t) \le \exp(\mathcal{C}_*),$$

$$\sup_{v\in\mathbb{N},t\in[0,\tau]} |S(\psi_{k,v};\eta_k^*,t)| \le \exp(\mathcal{C}_*)\mathcal{C}_\psi, \quad \sup_{v\in\mathbb{N},t\in[0,\tau]} \left|S_{(n)}(\psi_{k,v};\eta_k^*,t)\right| \le \exp(\mathcal{C}_*)\mathcal{C}_\psi,$$

$$\sup_{v,v'\in\mathbb{N},t\in[0,\tau]} |S(\psi_{k,v}\psi_{k,v'};\eta_k^*,t)| \le \exp(\mathcal{C}_*)\mathcal{C}_\psi^2, \quad \sup_{v,v'\in\mathbb{N},t\in[0,\tau]} \left|S_{(n)}(\psi_{k,v}\psi_{k,v'};\eta_k^*,t)\right| \le \exp(\mathcal{C}_*)\mathcal{C}_\psi^2,$$

$$\sup_{v\in\mathbb{N},t\in[0,\tau]} |\mathcal{U}(\psi_{k,v};\eta_k^*,t)| \le \mathcal{C}_\psi, \quad \sup_{v\in\mathbb{N},t\in[0,\tau]} \left|\mathcal{U}_{(n)}(\psi_{k,v};\eta_k^*,t)\right| \le \mathcal{C}_\psi,$$

$$\sup_{v,v'\in\mathbb{N},t\in[0,\tau]} |\mathcal{V}(\psi_{k,v},\psi_{k,v'};\eta_k^*,t)| \le 2\mathcal{C}_\psi^2, \quad \sup_{v,v'\in\mathbb{N},t\in[0,\tau]} \left|\mathcal{V}_{(n)}(\psi_{k,v},\psi_{k,v'};\eta_k^*,t)\right| \le 2\mathcal{C}_\psi^2.$$

Furthermore, for any $v, v' \in \mathbb{N}$, the following results hold:

$$\mathbb{E}\left[\int_0^\tau \frac{1}{S_{(n)}(\eta_k^*, t)} d\overline{N}_k(t)\right] \leq \mathcal{C}_{h,2}\tau,$$

$$\mathbb{E}\left[\int_0^\tau \frac{\left(S_{(n)}(\eta_k^*, t) - S(\eta_k^*, t)\right)^2}{S_{(n)}(\eta_k^*, t)} d\overline{N}_k(t)\right] \leq \frac{\exp(2\mathcal{C}_*)\mathcal{C}_{h,2}\tau}{n},$$

$$\mathbb{E}\left[\int_0^\tau \frac{\left(S_{(n)}(\psi_{k,v}; \eta_k^*, t) - S(\psi_{k,v}; \eta_k^*, t)\right)^2}{S_{(n)}(\eta_k^*, t)} d\overline{N}_k(t)\right] \leq \frac{\exp(2\mathcal{C}_*)\mathcal{C}_\psi^2\mathcal{C}_{h,2}\tau}{n},$$

$$\mathbb{E}\left[\int_0^\tau \frac{\left(S_{(n)}(\psi_{k,v}\psi_{k,v'}; \eta_k^*, t) - S(\psi_{k,v}\psi_{k,v'}; \eta_k^*, t)\right)^2}{S_{(n)}(\eta_k^*, t)} d\overline{N}_k(t)\right] \leq \frac{\exp(2\mathcal{C}_*)\mathcal{C}_\psi^4\mathcal{C}_{h,2}\tau}{n}.$$

Proof of Lemma S5. Considering $Y(\cdot), Y_i(\cdot) \leq 1$ and boundedness of η_k^*, we have

$$\sup_{t\in[0,\tau]} S(\eta_k^*, t) = \sup_{t\in[0,\tau]} \mathbb{E}\left[Y(t)\exp(\eta_k^*(X))\right] \leq \left\{\sup_{t\in[0,\tau]} \mathbb{E}\left[Y(t)\right]\right\} \exp(\mathcal{C}_*) \leq \exp(\mathcal{C}_*),$$

$$\sup_{t\in[0,\tau]} S_{(n)}(\eta_k^*, t) = \sup_{t\in[0,\tau]} \frac{1}{n}\sum_{i=1}^n Y_i(t)\exp(\eta_k^*(X_i)) \leq \left\{\sup_{t\in[0,\tau]} \frac{1}{n}\sum_{i=1}^n Y_i(t)\right\} \exp(\mathcal{C}_*) \leq \exp(\mathcal{C}_*).$$

Regarding boundedness of eigenfunctions, we have

$$\begin{aligned}
\sup_{v,v'\in\mathbb{N}, t\in[0,\tau]} \left|S(\psi_{k,v}\psi_{k,v'}; \eta_k^*, t)\right| &= \sup_{v,v'\in\mathbb{N}, t\in[0,\tau]} \left|\mathbb{E}\big[Y(t)\psi_{k,v}(X)\psi_{k,v'}(X)\exp(\eta_k^*(X))\big]\right| \\
&\leq \sup_{v,v'\in\mathbb{N}, t\in[0,\tau]} \mathbb{E}\Big[Y(t)\big|\psi_{k,v}(X)\big|\big|\psi_{k,v'}(X)\big|\exp(\eta_k^*(X))\Big] \\
&\leq \exp(\mathcal{C}_*)\mathcal{C}_\psi^2, \\
\sup_{v,v'\in\mathbb{N}, t\in[0,\tau]} \left|S_{(n)}(\psi_{k,v}\psi_{k,v'}; \eta_k^*, t)\right| &= \sup_{v,v'\in\mathbb{N}, t\in[0,\tau]} \left|\frac{1}{n}\sum_{i=1}^n Y_i(t)\psi_{k,v}(X_i)\psi_{k,v'}(X_i)\exp(\eta_k^*(X_i))\right| \\
&\leq \sup_{v,v'\in\mathbb{N}, t\in[0,\tau]} \frac{1}{n}\sum_{i=1}^n Y_i(t)\big|\psi_{k,v}(X_i)\big|\big|\psi_{k,v'}(X_i)\big|\exp(\eta_k^*(X_i)) \\
&\leq \exp(\mathcal{C}_*)\mathcal{C}_\psi^2,
\end{aligned}$$

where boundedness for $\sup_{v\in\mathbb{N}, t\in[0,\tau]} \left|S(\psi_{k,v}; \eta_k^*, t)\right|$ and $\sup_{v\in\mathbb{N}, t\in[0,\tau]} \left|S_{(n)}(\psi_{k,v}; \eta_k^*, t)\right|$ can be similarly proved.

$$\sup_{v,v'\in\mathbb{N},t\in[0,\tau]}\left|\frac{S(\psi_{k,v}\psi_{k,v'};\eta_k^*,t)}{S(\eta_k^*,t)}\right| \leq \sup_{v,v'\in\mathbb{N},t\in[0,\tau]}\frac{\mathbb{E}\big[Y(t)\big|\psi_{k,v}(X)\big|\big|\psi_{k,v'}(X)\big|\exp(\eta_k^*(X))\big]}{\mathbb{E}[Y(t)\exp(\eta_k^*(X))]}$$

$$\leq \left\{\sup_{v,v'\in\mathbb{N},t\in[0,\tau]}\frac{\mathbb{E}[Y(t)\exp(\eta_k^*(X))]}{\mathbb{E}[Y(t)\exp(\eta_k^*(X))]}\right\}\mathcal{C}_\psi^2 = \mathcal{C}_\psi^2,$$

$$\sup_{v,v'\in\mathbb{N},t\in[0,\tau]}\left|\frac{S_{(n)}(\psi_{k,v}\psi_{k,v'};\eta_k^*,t)}{S_{(n)}(\eta_k^*,t)}\right| \leq \sup_{v,v'\in\mathbb{N},t\in[0,\tau]}\frac{\frac{1}{n}\sum_{i=1}^n Y_i(t)\big|\psi_{k,v}(X_i)\big|\big|\psi_{k,v'}(X_i)\big|\exp(\eta_k^*(X_i))}{\frac{1}{n}\sum_{i=1}^n Y_i(t)\exp(\eta_k^*(X_i))}$$

$$\leq \left\{\sup_{v,v'\in\mathbb{N},t\in[0,\tau]}\frac{\frac{1}{n}\sum_{i=1}^n Y_i(t)\exp(\eta_k^*(X_i))}{\frac{1}{n}\sum_{i=1}^n Y_i(t)\exp(\eta_k^*(X_i))}\right\}\mathcal{C}_\psi^2 = \mathcal{C}_\psi^2,$$

where boundedness for $\sup_{v\in\mathbb{N},t\in[0,\tau]}|\mathcal{U}(\psi_{k,v};\eta_k^*,t)|$ and $\sup_{v\in\mathbb{N},t\in[0,\tau]}\left|\mathcal{U}_{(n)}(\psi_{k,v};\eta_k^*,t)\right|$ can be similarly proved. Combining above results,

$$\sup_{v,v'\in\mathbb{N},t\in[0,\tau]}|\mathcal{V}(\psi_{k,v},\psi_{k,v'};\eta_k^*,t)| \leq \sup_{v,v'\in\mathbb{N},t\in[0,\tau]}\left|\frac{S(\psi_{k,v}\psi_{k,v'};\eta_k^*,t)}{S(\eta_k^*,t)}\right| + \left\{\sup_{v\in\mathbb{N},t\in[0,\tau]}|\mathcal{U}(\psi_{k,v};\eta_k^*,t)|\right\}^2 \leq 2\mathcal{C}_\psi^2,$$

$$\sup_{v,v'\in\mathbb{N},t\in[0,\tau]}\left|\mathcal{V}_{(n)}(\psi_{k,v},\psi_{k,v'};\eta_k^*,t)\right| \leq \sup_{v,v'\in\mathbb{N},t\in[0,\tau]}\left|\frac{S_{(n)}(\psi_{k,v}\psi_{k,v'};\eta_k^*,t)}{S_{(n)}(\eta_k^*,t)}\right| + \left\{\sup_{v\in\mathbb{N},t\in[0,\tau]}\left|\mathcal{U}_{(n)}(\psi_{k,v};\eta_k^*,t)\right|\right\}^2 \leq 2\mathcal{C}_\psi^2,$$

We show boundedness of moment calculations, which are

$$\begin{aligned}\mathbb{E}\left[\int_0^\tau \frac{1}{S_{(n)}(\eta_k^*,t)}d\overline{N}_k(t)\right] &= \int_0^\tau \mathbb{E}\left[\frac{1}{S_{(n)}(\eta_k^*,t)}d\overline{N}_k(t)\right]\\ &= \int_0^\tau \mathbb{E}\left[\mathbb{E}\left[\frac{1}{S_{(n)}(\eta_k^*,t)}d\overline{N}_k(t)\middle|\mathcal{F}_{t-}\right]\right]\\ &= \int_0^\tau \mathbb{E}\left[\frac{1}{S_{(n)}(\eta_k^*,t)}\mathbb{E}\left[d\overline{N}_k(t)|\mathcal{F}_{t-}\right]\right]\\ &= \int_0^\tau \mathbb{E}\left[\frac{S_{(n)}(\eta_k^*,t)}{S_{(n)}(\eta_k^*,t)}dH_{0k}(t)\right] = H_{0k}(\tau) \leq \mathcal{C}_{h,2}\tau,\end{aligned}$$

$$
\begin{aligned}
&\mathbb{E}\left[\int_0^\tau \frac{\left(S_{(n)}(\psi_{k,v}\psi_{k,v'};\eta_k^*,t)-S(\psi_{k,v}\psi_{k,v'};\eta_k^*,t)\right)^2}{S_{(n)}(\eta_k^*,t)}d\overline{N}_k(t)\right]\\
&\quad=\int_0^\tau \mathbb{E}\left[\mathbb{E}\left[\frac{\left(S_{(n)}(\psi_{k,v}\psi_{k,v'};\eta_k^*,t)-S(\psi_{k,v}\psi_{k,v'};\eta_k^*,t)\right)^2}{S_{(n)}(\eta_k^*,t)}d\overline{N}_k(t)\middle|\mathcal{F}_{t^-}\right]\right]\\
&\quad=\int_0^\tau \mathbb{E}\left[\frac{\left(S_{(n)}(\psi_{k,v}\psi_{k,v'};\eta_k^*,t)-S(\psi_{k,v}\psi_{k,v'};\eta_k^*,t)\right)^2}{S_{(n)}(\eta_k^*,t)}\mathbb{E}\left[d\overline{N}_k(t)|\mathcal{F}_{t^-}\right]\right]\\
&\quad=\int_0^\tau \mathbb{E}\left[\left(S_{(n)}(\psi_{k,v}\psi_{k,v'};\eta_k^*,t)-S(\psi_{k,v}\psi_{k,v'};\eta_k^*,t)\right)^2\right]dH_{0k}(t)\\
&\quad=\frac{1}{n}\int_0^\tau \mathrm{Var}\Big[Y(t)\psi_{k,v}(X)\psi_{k,v'}(X)\exp(\eta_k^*(X))\Big]dH_{0k}(t)\\
&\quad\le\frac{1}{n}\int_0^\tau \mathbb{E}\Big[\big(Y(t)\psi_{k,v}(X)\psi_{k,v'}(X)\exp(\eta_k^*(X))\big)^2\Big]dH_{0k}(t)\\
&\quad\le\frac{1}{n}\mathcal{C}_\psi^2\mathcal{C}_\psi^2\exp(2\mathcal{C}_*)H_{0k}(\tau)\le\frac{\exp(2\mathcal{C}_*)\mathcal{C}_\psi^4\mathcal{C}_{h,2}\tau}{n},
\end{aligned}
$$

where $\mathbb{E}_W\left[Y(t)\psi_{k,v}(X)\psi_{k,v'}(X)\exp(\eta_k^*(X))\right]=\mathbb{E}\left[Y(t)\psi_{k,v}(X)\psi_{k,v'}(X)\exp(\eta_k^*(X))\right]$. Similar derivation yields

$$
\begin{aligned}
\mathbb{E}\left[\int_0^\tau \frac{\left(S_{(n)}(\psi_{k,v};\eta_k^*,t)-S(\psi_{k,v};\eta_k^*,t)\right)^2}{S_{(n)}(\eta_k^*,t)}d\overline{N}_k(t)\right]&=\frac{1}{n}\int_0^\tau \mathrm{Var}\Big[Y(t)\psi_{k,v}(X)\exp(\eta_k^*(X))\Big]dH_{0k}(t)\\
&\le\frac{1}{n}\int_0^\tau \mathbb{E}\Big[\big(Y(t)\psi_{k,v}(X)\exp(\eta_k^*(X))\big)^2\Big]dH_{0k}(t)\\
&\le\frac{\exp(2\mathcal{C}_*)\mathcal{C}_\psi^2\mathcal{C}_{h,2}\tau}{n},
\end{aligned}
$$

$$
\begin{aligned}
\mathbb{E}\left[\int_0^\tau \frac{\left(S_{(n)}(\eta_k^*,t)-S(\eta_k^*,t)\right)^2}{S_{(n)}(\eta_k^*,t)}d\overline{N}_k(t)\right]&=\frac{1}{n}\int_0^\tau \mathrm{Var}\Big[Y(t)\exp(\eta_k^*(X))\Big]dH_{0k}(t)\\
&\le\frac{1}{n}\int_0^\tau \mathbb{E}\Big[\big(Y(t)\exp(\eta_k^*(X))\big)^2\Big]dH_{0k}(t)\le\frac{\exp(2\mathcal{C}_*)\mathcal{C}_{h,2}\tau}{n}.
\end{aligned}
$$

■

Proof of Lemma S2. Here, each $f\in\mathcal{H}$ or $g\in\mathcal{H}$ can represent either a deterministic function or a stochastic process. We use the fact that $\mathbb{E}(|X_n|^b)=\mathcal{O}(a_n^b)$ implies $X_n=\mathcal{O}_{\mathbb{P}}(a_n)$, and $\mathbb{E}(|X_n|^b)=o(a_n^b)$ implies $X_n=o_{\mathbb{P}}(a_n)$, where $b\in(0,\infty)$ is a constant and for $n\in\mathbb{N}$, $X_n\in\mathbb{R}$ is a random sequence

and $a_n \in (0, \infty)$ is a deterministic sequence. Note that Lemma S2 and its proof are similar to the result in O'Sullivan (1993).

The upper bound of the difference between $\mathcal{V}_k^{(n)}(f,g)$ and $\mathcal{V}_k(f,g)$ is given by

$$\left|\mathcal{V}_k^{(n)}(f,g) - \mathcal{V}_k(f,g)\right| \leq \left|\int_0^\tau \left\{\mathcal{V}_{(n)}(f,g;\eta_k^*,t) - \mathcal{V}(f,g;\eta_k^*,t)\right\} d\overline{N}_k(t)\right| + \left|\int_0^\tau \mathcal{V}(f,g;\eta_k^*,t)d\overline{N}_k(t) - \mathcal{V}_k(f,g)\right|, \tag{S2.19}$$

where we demonstrate the asymptotic order bounds for the first and second components on the right-hand side as follows.

For the second component in (S2.19), we have

$$\begin{aligned}
&\left|\int_0^\tau \mathcal{V}(f,g;\eta_k^*,t)d\overline{N}_k(t) - \mathcal{V}_k(f,g)\right| \\
&= \left|\sum_{v\in\mathbb{N}}\sum_{v'\in\mathbb{N}} \mathcal{V}_k(f,\psi_{k,v})\mathcal{V}_k(g,\psi_{k,v'})\left\{\int_0^\tau \mathcal{V}(\psi_{k,v},\psi_{k,v'};\eta_k^*,t)d\overline{N}_k(t) - \mathcal{V}_k(\psi_{k,v},\psi_{k,v'})\right\}\right| \\
&\leq \left\{\sum_{v\in\mathbb{N}}\sum_{v'\in\mathbb{N}} (1+\lambda_k\rho_{k,v})(1+\lambda_k\rho_{k,v'})\mathcal{V}_k^2(f,\psi_{k,v})\mathcal{V}_k^2(g,\psi_{k,v'})\right\}^{1/2} \\
&\times \left\{\sum_{v\in\mathbb{N}}\sum_{v'\in\mathbb{N}} \frac{1}{1+\lambda_k\rho_{k,v}}\frac{1}{1+\lambda_k\rho_{k,v'}}\left\{\int_0^\tau \mathcal{V}(\psi_{k,v},\psi_{k,v'};\eta_k^*,t)d\overline{N}_k(t) - \mathcal{V}_k(\psi_{k,v},\psi_{k,v'})\right\}^2\right\}^{1/2} \\
&= \sqrt{(\mathcal{V}_k+\lambda_k J_k)(f)(\mathcal{V}_k+\lambda_k J_k)(g)}\,\mathcal{O}_{\mathbb{P}}\left(n^{-1/2}\lambda_k^{-1/r}\right),
\end{aligned} \tag{S2.20}$$

where the last equality is obtained by

$$\begin{aligned}
&\mathbb{E}\left|\sum_{v\in\mathbb{N}}\sum_{v'\in\mathbb{N}} \frac{1}{1+\lambda_k\rho_{k,v}}\frac{1}{1+\lambda_k\rho_{k,v'}}\left\{\int_0^\tau \mathcal{V}(\psi_{k,v},\psi_{k,v'};\eta_k^*,t)d\overline{N}_k(t) - \mathcal{V}_k(\psi_{k,v},\psi_{k,v'})\right\}^2\right| \\
&= \mathbb{E}\left[\sum_{v\in\mathbb{N}}\sum_{v'\in\mathbb{N}} \frac{1}{1+\lambda_k\rho_{k,v}}\frac{1}{1+\lambda_k\rho_{k,v'}}\left\{\int_0^\tau \mathcal{V}(\psi_{k,v},\psi_{k,v'};\eta_k^*,t)d\overline{N}_k(t) - \mathcal{V}_k(\psi_{k,v},\psi_{k,v'})\right\}^2\right] \\
&\leq \left\{\sum_{v\in\mathbb{N}}\sum_{v'\in\mathbb{N}} \frac{1}{1+\lambda_k\rho_{k,v}}\frac{1}{1+\lambda_k\rho_{k,v'}}\right\}\frac{4\mathcal{C}_\psi^4}{n} = \mathcal{O}\left(\lambda_k^{-1/r}\right)\mathcal{O}\left(\lambda_k^{-1/r}\right)\frac{4\mathcal{C}_\psi^4}{n} = \mathcal{O}\left(n^{-1}\lambda_k^{-2/r}\right).
\end{aligned}$$

The last inequality above is obtained by

$$\mathbb{E}\left[\left\{\frac{1}{n}\sum_{i=1}^n\left\{\int_0^\tau \mathcal{V}(\psi_{k,v},\psi_{k,v'};\eta_k^*,t)dN_{ik}(t) - \mathcal{V}_k(\psi_{k,v},\psi_{k,v'})\right\}\right\}^2\right]$$

$$= \frac{1}{n}\mathrm{Var}\left[\int_0^\tau \mathcal{V}(\psi_{k,v},\psi_{k,v'};\eta_k^*,t)dN_k(t)\right] \le \frac{1}{n}\mathbb{E}\left[\left\{\int_0^\tau \mathcal{V}(\psi_{k,v},\psi_{k,v'};\eta_k^*,t)dN_k(t)\right\}^2\right] \le \frac{4\mathcal{C}_\psi^4}{n},$$

where

$$\begin{aligned}
\mathbb{E}\left[\int_0^\tau \mathcal{V}(\psi_{k,v},\psi_{k,v'};\eta_k^*,t)dN_{ik}(t)\right] &= \int_0^\tau \mathcal{V}(\psi_{k,v},\psi_{k,v'};\eta_k^*,t)\mathbb{E}\big[\mathbb{E}\left[dN_{ik}(t)|\mathcal{F}_{t-}\right]\big] \\
&= \int_0^\tau \mathcal{V}(\psi_{k,v},\psi_{k,v'};\eta_k^*,t)\mathbb{E}\left[Y_i(t)\exp(\eta_k^*(X_i))\right]dH_{0k}(t) \\
&= \int_0^\tau \mathcal{V}(\psi_{k,v},\psi_{k,v'};\eta_k^*,t)\mathbb{E}_W\left[Y_i(t)\exp(\eta_k^*(X_i))\right]dH_{0k}(t) \\
&= \int_0^\tau \mathcal{V}(\psi_{k,v},\psi_{k,v'};\eta_k^*,t)S(\eta_k^*,t)dH_{0k}(t) = \mathcal{V}_k(\psi_{k,v},\psi_{k,v'}),
\end{aligned}$$

$$\begin{aligned}
\left|\int_0^\tau \mathcal{V}(\psi_{k,v},\psi_{k,v'};\eta_k^*,t)dN_k(t)\right| &\le \int_0^\tau \left|\mathcal{V}(\psi_{k,v},\psi_{k,v'};\eta_k^*,t)\right| dN_k(t) \\
&\le \left\{\sup_{t\in[0,\tau]} \left|\mathcal{V}(\psi_{k,v},\psi_{k,v'};\eta_k^*,t)\right|\right\} N_k(\tau) \le 2\mathcal{C}_\psi^2.
\end{aligned}$$

For the first component in (S2.19), simple calculation yields

$$\begin{aligned}
&\left|\int_0^\tau \left\{\mathcal{V}_{(n)}(f,g;\eta_k^*,t) - \mathcal{V}(f,g;\eta_k^*,t)\right\} d\overline{N}_k(t)\right| \qquad \text{(S2.21)} \\
&\le \left|\int_0^\tau \frac{S_{(n)}(fg;\eta_k^*,t) - S(fg;\eta_k^*,t)}{S_{(n)}(\eta_k^*,t)} d\overline{N}_k(t)\right| \\
&+ \left|\int_0^\tau \frac{S(fg;\eta_k^*,t)}{S(\eta_k^*,t)} \frac{S(\eta_k^*,t) - S_{(n)}(\eta_k^*,t)}{S_{(n)}(\eta_k^*,t)} d\overline{N}_k(t)\right| \\
&+ \left|\int_0^\tau \frac{S_{(n)}(f;\eta_k^*,t)}{S_{(n)}(\eta_k^*,t)} \frac{S_{(n)}(g;\eta_k^*,t) - S(g;\eta_k^*,t)}{S_{(n)}(\eta_k^*,t)} d\overline{N}_k(t)\right| \\
&+ \left|\int_0^\tau \frac{S_{(n)}(f;\eta_k^*,t)}{S_{(n)}(\eta_k^*,t)} \frac{S(g;\eta_k^*,t)}{S(\eta_k^*,t)} \frac{S(\eta_k^*,t) - S_{(n)}(\eta_k^*,t)}{S_{(n)}(\eta_k^*,t)} d\overline{N}_k(t)\right| \\
&+ \left|\int_0^\tau \frac{S(g;\eta_k^*,t)}{S(\eta_k^*,t)} \frac{S_{(n)}(f;\eta_k^*,t) - S(f;\eta_k^*,t)}{S_{(n)}(\eta_k^*,t)} d\overline{N}_k(t)\right| \\
&+ \left|\int_0^\tau \frac{S(g;\eta_k^*,t)}{S(\eta_k^*,t)} \frac{S(f;\eta_k^*,t)}{S(\eta_k^*,t)} \frac{S(\eta_k^*,t) - S_{(n)}(\eta_k^*,t)}{S_{(n)}(\eta_k^*,t)} d\overline{N}_k(t)\right|.
\end{aligned}$$

For the first component of (S2.21), we have

$$
\begin{aligned}
&\left|\int_0^\tau \frac{S_{(n)}(fg;\eta_k^*,t)-S(fg;\eta_k^*,t)}{S_{(n)}(\eta_k^*,t)}d\overline{N}_k(t)\right| \\
&=\left|\sum_{v\in\mathbb{N}}\sum_{v'\in\mathbb{N}}\mathcal{V}_k(f,\psi_{k,v})\mathcal{V}_k(g,\psi_{k,v'})\int_0^\tau \frac{S_{(n)}(\psi_{k,v}\psi_{k,v'};\eta_k^*,t)-S(\psi_{k,v}\psi_{k,v'};\eta_k^*,t)}{S_{(n)}(\eta_k^*,t)}d\overline{N}_k(t)\right| \\
&\le\left\{\sum_{v\in\mathbb{N}}\sum_{v'\in\mathbb{N}}(1+\lambda_k\rho_{k,v})(1+\lambda_k\rho_{k,v'})\mathcal{V}_k^2(f,\psi_{k,v})\mathcal{V}_k^2(g,\psi_{k,v'})\right\}^{1/2} \\
&\times\left\{\sum_{v\in\mathbb{N}}\sum_{v'\in\mathbb{N}}\frac{1}{1+\lambda_k\rho_{k,v}}\frac{1}{1+\lambda_k\rho_{k,v'}}\left\{\int_0^\tau \frac{S_{(n)}(\psi_{k,v}\psi_{k,v'};\eta_k^*,t)-S(\psi_{k,v}\psi_{k,v'};\eta_k^*,t)}{S_{(n)}(\eta_k^*,t)}d\overline{N}_k(t)\right\}^2\right\}^{1/2} \\
&=\sqrt{(\mathcal{V}_k+\lambda_kJ_k)(f)(\mathcal{V}_k+\lambda_kJ_k)(g)}\,\mathcal{O}_{\mathbb{P}}\left(n^{-1/2}\lambda_k^{-1/r}\right). \qquad \text{(S2.22)}
\end{aligned}
$$

The last equality in (S2.22) is obtained by

$$
\begin{aligned}
0&\le\sum_{v\in\mathbb{N}}\sum_{v'\in\mathbb{N}}\frac{1}{1+\lambda_k\rho_{k,v}}\frac{1}{1+\lambda_k\rho_{k,v'}}\left\{\int_0^\tau \frac{S_{(n)}(\psi_{k,v}\psi_{k,v'};\eta_k^*,t)-S(\psi_{k,v}\psi_{k,v'};\eta_k^*,t)}{S_{(n)}^{1/2}(\eta_k^*,t)S_{(n)}^{1/2}(\eta_k^*,t)}d\overline{N}_k(t)\right\}^2 \\
&\le\left\{\sum_{v\in\mathbb{N}}\sum_{v'\in\mathbb{N}}\frac{1}{1+\lambda_k\rho_{k,v}}\frac{1}{1+\lambda_k\rho_{k,v'}}\int_0^\tau \frac{\left(S_{(n)}(\psi_{k,v}\psi_{k,v'};\eta_k^*,t)-S(\psi_{k,v}\psi_{k,v'};\eta_k^*,t)\right)^2}{S_{(n)}(\eta_k^*,t)}d\overline{N}_k(t)\right\} \\
&\times\int_0^\tau \frac{1}{S_{(n)}(\eta_k^*,t)}d\overline{N}_k(t)=Z_{1,1}\times Z_{1,2}=\mathcal{O}_{\mathbb{P}}\left(n^{-1}\lambda_k^{-2/r}\right)\mathcal{O}_{\mathbb{P}}(1)=\mathcal{O}_{\mathbb{P}}\left(n^{-1}\lambda_k^{-2/r}\right),
\end{aligned}
$$

where

$$
\mathbb{E}|Z_{1,1}|=\mathbb{E}[Z_{1,1}]\le\left\{\sum_{v\in\mathbb{N}}\sum_{v'\in\mathbb{N}}\frac{1}{1+\lambda_k\rho_{k,v}}\frac{1}{1+\lambda_k\rho_{k,v'}}\right\}\frac{\exp(2\mathcal{C}_*)\mathcal{C}_\psi^4\mathcal{C}_{h,2}\tau}{n}=\mathcal{O}\left(n^{-1}\lambda_k^{-2/r}\right)
$$

and $\mathbb{E}|Z_{1,2}|=\mathbb{E}[Z_{1,2}]\le\mathcal{C}_{h,2}\tau=\mathcal{O}(1)$.

For the second component of (S2.21), we have

$$
\begin{aligned}
&\left|\int_0^\tau \frac{S(fg;\eta_k^*,t)}{S(\eta_k^*,t)}\frac{S(\eta_k^*,t)-S_{(n)}(\eta_k^*,t)}{S_{(n)}(\eta_k^*,t)}d\overline{N}_k(t)\right| \\
&=\left|\sum_{v\in\mathbb{N}}\sum_{v'\in\mathbb{N}}\mathcal{V}_k(f,\psi_{k,v})\mathcal{V}_k(g,\psi_{k,v'})\int_0^\tau \frac{S(\psi_{k,v}\psi_{k,v'};\eta_k^*,t)}{S(\eta_k^*,t)}\frac{S(\eta_k^*,t)-S_{(n)}(\eta_k^*,t)}{S_{(n)}(\eta_k^*,t)}d\overline{N}_k(t)\right|
\end{aligned}
$$

$$\leq \left\{ \sum_{v \in \mathbb{N}} \sum_{v' \in \mathbb{N}} (1+\lambda_k \rho_{k,v})(1+\lambda_k \rho_{k,v'}) \mathcal{V}_k^2(f, \psi_{k,v}) \mathcal{V}_k^2(g, \psi_{k,v'}) \right\}^{1/2}$$

$$\times \left\{ \sum_{v \in \mathbb{N}} \sum_{v' \in \mathbb{N}} \frac{1}{1+\lambda_k \rho_{k,v}} \frac{1}{1+\lambda_k \rho_{k,v'}} \left\{ \int_0^\tau \frac{S(\psi_{k,v}\psi_{k,v'}; \eta_k^*, t)}{S(\eta_k^*, t)} \frac{S(\eta_k^*, t) - S_{(n)}(\eta_k^*, t)}{S_{(n)}(\eta_k^*, t)} d\overline{N}_k(t) \right\}^2 \right\}^{1/2}$$

$$= \sqrt{(\mathcal{V}_k + \lambda_k J_k)(f)(\mathcal{V}_k + \lambda_k J_k)(g)} \mathcal{O}_{\mathbb{P}} \left(n^{-1/2} \lambda_k^{-1/r} \right). \tag{S2.23}$$

The last equality in (S2.23) is obtained by

$$0 \leq \sum_{v \in \mathbb{N}} \sum_{v' \in \mathbb{N}} \frac{1}{1+\lambda_k \rho_{k,v}} \frac{1}{1+\lambda_k \rho_{k,v'}} \left\{ \int_0^\tau \frac{S(\psi_{k,v}\psi_{k,v'}; \eta_k^*, t)}{S(\eta_k^*, t)} \frac{S(\eta_k^*, t) - S_{(n)}(\eta_k^*, t)}{S_{(n)}(\eta_k^*, t)} d\overline{N}_k(t) \right\}^2$$

$$\leq \left\{ \sum_{v \in \mathbb{N}} \sum_{v' \in \mathbb{N}} \frac{1}{1+\lambda_k \rho_{k,v}} \frac{1}{1+\lambda_k \rho_{k,v'}} \int_0^\tau \frac{\left(S_{(n)}(\eta_k^*, t) - S(\eta_k^*, t)\right)^2}{S_{(n)}(\eta_k^*, t)} d\overline{N}_k(t) \right\}$$
$$\times \mathcal{C}_\psi^4 \int_0^\tau \frac{1}{S_{(n)}(\eta_k^*, t)} d\overline{N}_k(t) = Z_{2,1} \times Z_{2,2} = \mathcal{O}_{\mathbb{P}} \left(n^{-1} \lambda_k^{-2/r} \right) \mathcal{O}_{\mathbb{P}}(1) = \mathcal{O}_{\mathbb{P}} \left(n^{-1} \lambda_k^{-2/r} \right),$$

where

$$\left| \int_0^\tau \frac{S(\psi_{k,v}\psi_{k,v'}; \eta_k^*, t)}{S(\eta_k^*, t)} \frac{S(\eta_k^*, t) - S_{(n)}(\eta_k^*, t)}{S_{(n)}(\eta_k^*, t)} d\overline{N}_k(t) \right|$$
$$\leq \int_0^\tau \left| \frac{S(\psi_{k,v}\psi_{k,v'}; \eta_k^*, t)}{S(\eta_k^*, t)} \frac{S(\eta_k^*, t) - S_{(n)}(\eta_k^*, t)}{S_{(n)}(\eta_k^*, t)} \right| d\overline{N}_k(t)$$
$$\leq \left\{ \sup_{t \in [0,\tau]} \left| \frac{S(\psi_{k,v}\psi_{k,v'}; \eta_k^*, t)}{S(\eta_k^*, t)} \right| \right\} \times \left| \int_0^\tau \frac{\left| S_{(n)}(\eta_k^*, t) - S(\eta_k^*, t) \right|}{S_{(n)}^{1/2}(\eta_k^*, t) S_{(n)}^{1/2}(\eta_k^*, t)} d\overline{N}_k(t) \right|$$
$$\leq \mathcal{C}_\psi^2 \left\{ \int_0^\tau \frac{\left| S_{(n)}(\eta_k^*, t) - S(\eta_k^*, t) \right|^2}{S_{(n)}(\eta_k^*, t)} d\overline{N}_k(t) \right\}^{1/2} \left\{ \int_0^\tau \frac{1}{S_{(n)}(\eta_k^*, t)} d\overline{N}_k(t) \right\}^{1/2},$$

$$\mathbb{E}|Z_{2,1}| = \mathbb{E}[Z_{2,1}] \leq \left\{ \sum_{v \in \mathbb{N}} \sum_{v' \in \mathbb{N}} \frac{1}{1+\lambda_k \rho_{k,v}} \frac{1}{1+\lambda_k \rho_{k,v'}} \right\} \frac{\exp(2\mathcal{C}_*)\mathcal{C}_{h,2}\tau}{n} = \mathcal{O}\left(n^{-1} \lambda_k^{-2/r} \right),$$

and $\mathbb{E}|Z_{2,2}| = \mathbb{E}[Z_{2,2}] \leq \mathcal{C}_\psi^4 \mathcal{C}_{h,2}\tau = \mathcal{O}(1)$.

For the remaining components of (S2.21), an analogous derivation yields the same order. Considering (S2.21), (S2.22), and (S2.23), we get

$$\left| \int_0^\tau \left\{ \mathcal{V}_{(n)}(f, g; \eta_k^*, t) - \mathcal{V}(f, g; \eta_k^*, t) \right\} d\overline{N}_k(t) \right| \tag{S2.24}$$

$$= \sqrt{(\mathcal{V}_k + \lambda_k J_k)(f)(\mathcal{V}_k + \lambda_k J_k)(g)}\mathcal{O}_{\mathbb{P}}\left(n^{-1/2}\lambda_k^{-1/r}\right).$$

Combining (S2.19), (S2.20), and (S2.24), we obtain

$$\left|\mathcal{V}_k^{(n)}(f,g) - \mathcal{V}_k(f,g)\right| = \sqrt{(\mathcal{V}_k + \lambda_k J_k)(f)(\mathcal{V}_k + \lambda_k J_k)(g)}\mathcal{O}_{\mathbb{P}}\left(n^{-1/2}\lambda_k^{-1/r}\right),$$

where $n^{-1/2}\lambda_k^{-1/r} \ll q_k^{-1/2}\lambda_k^{-1/r} = o(1)$ completes the proof. ■

Proof of Lemma S3. Here, each $f \in \mathcal{H}$ or $g \in \mathcal{H}$ can represent either a deterministic function or a stochastic process. For $\tilde{\alpha} \in [0,1]$, let $D_k(\tilde{\alpha}) = \mathcal{U}_{g+\tilde{\alpha}(f-g),k}^{(n)}(f-g)$. By the mean value theorem, for some random variable $\alpha \in (0,1)$, we have

$$\mathcal{U}_{f,k}^{(n)}(f-g) - \mathcal{U}_{g,k}^{(n)}(f-g) = \frac{D_k(1) - D_k(0)}{1-0} = \frac{\partial}{\partial\alpha}D_k(\alpha) = \mathcal{V}_{g+\alpha(f-g),k}^{(n)}(f-g).$$

■

Proof of Lemma S4. Here, $h \in \mathcal{H}$ can represent either a deterministic function or a stochastic process. Let $\sigma_1 = \{\mathcal{V}_k^{(n)}(f_k - \eta_k^*)\}^{1/2}$ and $C_1(\alpha_1) = \mathcal{U}_{\eta_k^*+\alpha_1(f_k-\eta_k^*)/\sigma_1,k}^{(n)}(h) - \mathcal{U}_k^{(n)}(h)$. For $\alpha_1 \approx 0$, by the Taylor series expansion of $C_1(\alpha_1)$ at 0, we get

$$\begin{aligned} C_1(\alpha_1) &= C_1(0) + C_1'(0)\alpha_1 + o_{\alpha_1}\left(C_1'(0)\alpha_1\right) = \alpha_1 C_1'(0)\left(1 + o_{\alpha_1}(1)\right) \\ &= \frac{\alpha_1}{\sigma_1}\mathcal{V}_k^{(n)}(f_k - \eta_k^*, h)\left(1 + o_{\alpha_1}(1)\right), \end{aligned}$$

where $C_1'(\alpha_1) = \partial C_1(\alpha_1)/(\partial\alpha_1)$ and $o_{\alpha_1}(\cdot)$ is $o(\cdot)$ with respect to $\alpha_1 \to 0$. As $\sigma_1 \approx 0$, we have

$$\mathcal{U}_{f_k,k}^{(n)}(h) - \mathcal{U}_k^{(n)}(h) = C_1(\alpha_1 = \sigma_1) = \mathcal{V}_k^{(n)}(f_k - \eta_k^*, h)\left(1 + o_{\mathbb{P}}(1)\right),$$

where the fact that $\sigma_1 = o_{\mathbb{P}}(1)$ as $n \to \infty$ leads to the substitution of $o_{\alpha_1=\sigma_1}(\cdot)$ with $o_{\mathbb{P}}(\cdot)$ as $n \to \infty$.

Let

$$\sigma_2 = \max\left\{\{\mathcal{V}_k^{(n)}(f_k - \eta_k^*)\}^{1/2}, \{\mathcal{V}_k^{(n)}(g_k - \eta_k^*)\}^{1/2}\right\}$$

and $C_2(\alpha_2) = \mathcal{U}_{\eta_k^*+\alpha_2(f_k-\eta_k^*)/\sigma_2,k}^{(n)}(h) - \mathcal{U}_{\eta_k^*+\alpha_2(g_k-\eta_k^*)/\sigma_2,k}^{(n)}(h)$. For $\alpha_2 \approx 0$, by the Taylor series expansion of $C_2(\alpha_2)$ at 0, we get

$$\begin{aligned} C_2(\alpha_2) &= C_2(0) + C_2'(0)\alpha_2 + o_{\alpha_2}\left(C_2'(0)\alpha_2\right) = \alpha_2 C_2'(0)\left(1 + o_{\alpha_2}(1)\right) \\ &= \frac{\alpha_2}{\sigma_2}\left(\mathcal{V}_k^{(n)}(f_k - \eta_k^*, h) - \mathcal{V}_k^{(n)}(g_k - \eta_k^*, h)\right)\left(1 + o_{\alpha_2}(1)\right) = \frac{\alpha_2}{\sigma_2}\mathcal{V}_k^{(n)}(f_k - g_k, h)\left(1 + o_{\alpha_2}(1)\right), \end{aligned}$$

where $C_2'(\alpha_2) = \partial C_2(\alpha_2)/(\partial\alpha_2)$ and $o_{\alpha_2}(\cdot)$ is $o(\cdot)$ with respect to $\alpha_2 \to 0$. As $\sigma_2 \approx 0$, we have

$$\mathcal{U}_{f_k,k}^{(n)}(h) - \mathcal{U}_{g_k,k}^{(n)}(h) = C_2(\alpha_2 = \sigma_2) = \mathcal{V}_k^{(n)}(f_k - g_k, h)\left(1 + o_{\mathbb{P}}(1)\right),$$

where the fact that $\sigma_2 = o_{\mathbb{P}}(1)$ as $n \to \infty$ leads to the substitution of $o_{\alpha_2=\sigma_2}(\cdot)$ with $o_{\mathbb{P}}(\cdot)$ as $n \to \infty$. ■

Lemma S6 *Under Assumption B.1, for each* k, $J_k(\eta_k, \hat{\eta}_k^* - \eta_k) = 0$ *and* $J_k(\hat{\eta}_k, \hat{\eta}_k^* - \eta_k) = 0$.

Proof of Lemma S6. Considering $\eta_k = \operatorname{arginf}_{\eta \in \mathcal{H}^{(k)}} \langle \hat{\eta}_k^* - \eta, \hat{\eta}_k^* - \eta \rangle_{\mathcal{H},k} \in \mathcal{H}^{(k)}$, we have $\langle f, \hat{\eta}_k^* - \eta_k \rangle_{\mathcal{H},k} = 0$ for all $f \in \mathcal{H}^{(k)}$. By the representer theorem, $\hat{\eta}_k^* \in \mathcal{H}_{(n)}^{(k)}$, where $\mathcal{H}^{(k)} \subseteq \mathcal{H}_{(n)}^{(k)} \equiv \mathcal{N}_J \oplus \mathcal{H}_{J(n)}^{(k)} \subseteq \mathcal{H}$ with $\mathcal{H}_J^{(k)} \subseteq \mathcal{H}_{J(n)}^{(k)} \equiv \mathrm{Span}\{\mathcal{K}_{J_k}(X_1, \cdot), \ldots, \mathcal{K}_{J_k}(X_n, \cdot)\} \subseteq \mathcal{H}_J$. By projecting with respect to $\langle \cdot, \cdot \rangle_{\mathcal{H},k}$ onto subspaces, one has $\hat{\eta}_k^* = \hat{\eta}_{k,N}^* + \hat{\eta}_{k,J}^*$, $\eta_k = \eta_{k,N} + \eta_{k,J}$, and $\hat{\eta}_k = \hat{\eta}_{k,N} + \hat{\eta}_{k,J}$, where

$$\hat{\eta}_{k,N}^*, \eta_{k,N}, \hat{\eta}_{k,N} \in \mathcal{N}_J,\ \hat{\eta}_{k,J}^* \in \mathcal{H}_{J(n)}^{(k)}, \text{ and } \eta_{k,J}, \hat{\eta}_{k,J} \in \mathcal{H}_J^{(k)}.$$

Note that

$$\langle \hat{\eta}_k^* - \eta_k, \hat{\eta}_k^* - \eta_k \rangle_{\mathcal{H},k} = \langle \hat{\eta}_{k,N}^* - \eta_{k,N}, \hat{\eta}_{k,N}^* - \eta_{k,N} \rangle_{\mathcal{N}_J} + J_k(\hat{\eta}_{k,J}^* - \eta_{k,J}, \hat{\eta}_{k,J}^* - \eta_{k,J}),$$

where, by the definition of η_k, we have $\eta_{k,N} = \hat{\eta}_{k,N}^* \in \mathcal{N}_J$, which minimizes $\langle \cdot, \cdot \rangle_{\mathcal{N}_J}$ term in above to zero. As a result, $\hat{\eta}_k^* - \eta_k = \hat{\eta}_{k,J}^* - \eta_{k,J}$. Considering $\eta_{k,J}, \hat{\eta}_{k,J} \in \mathcal{H}_J^{(k)} \subseteq \mathcal{H}^{(k)}$, we have $0 = \langle \eta_{k,J}, \hat{\eta}_k^* - \eta_k \rangle_{\mathcal{H},k} = \langle \eta_{k,J}, \hat{\eta}_{k,J}^* - \eta_{k,J} \rangle_{\mathcal{H},k} = J_k(\eta_{k,J}, \hat{\eta}_{k,J}^* - \eta_{k,J})$ and $0 = \langle \hat{\eta}_{k,J}, \hat{\eta}_k^* - \eta_k \rangle_{\mathcal{H},k} = \langle \hat{\eta}_{k,J}, \hat{\eta}_{k,J}^* - \eta_{k,J} \rangle_{\mathcal{H},k} = J_k(\hat{\eta}_{k,J}, \hat{\eta}_{k,J}^* - \eta_{k,J})$.

Finally, we get $J_k(\eta_k, \hat{\eta}_k^* - \eta_k) = J_k(\eta_{k,N}, \hat{\eta}_{k,J}^* - \eta_{k,J}) + J_k(\eta_{k,J}, \hat{\eta}_{k,J}^* - \eta_{k,J}) = 0$ and $J_k(\hat{\eta}_k, \hat{\eta}_k^* - \eta_k) = J_k(\hat{\eta}_{k,N}, \hat{\eta}_{k,J}^* - \eta_{k,J}) + J_k(\hat{\eta}_{k,J}, \hat{\eta}_{k,J}^* - \eta_{k,J}) = 0$. ■

S3 Additional Simulation Results

In this section, we provide additional simulation results corresponding to the studies in Section 3. For the simulation results for the additive models in Section 3.1, the estimation performance and Bayesian confidence interval performance are displayed in Figures S1 – S6. For the models with interactions considered in Section 3.2. Tables S1 – S4 report the data summary, estimation performance, and Bayesian confidence interval performance.

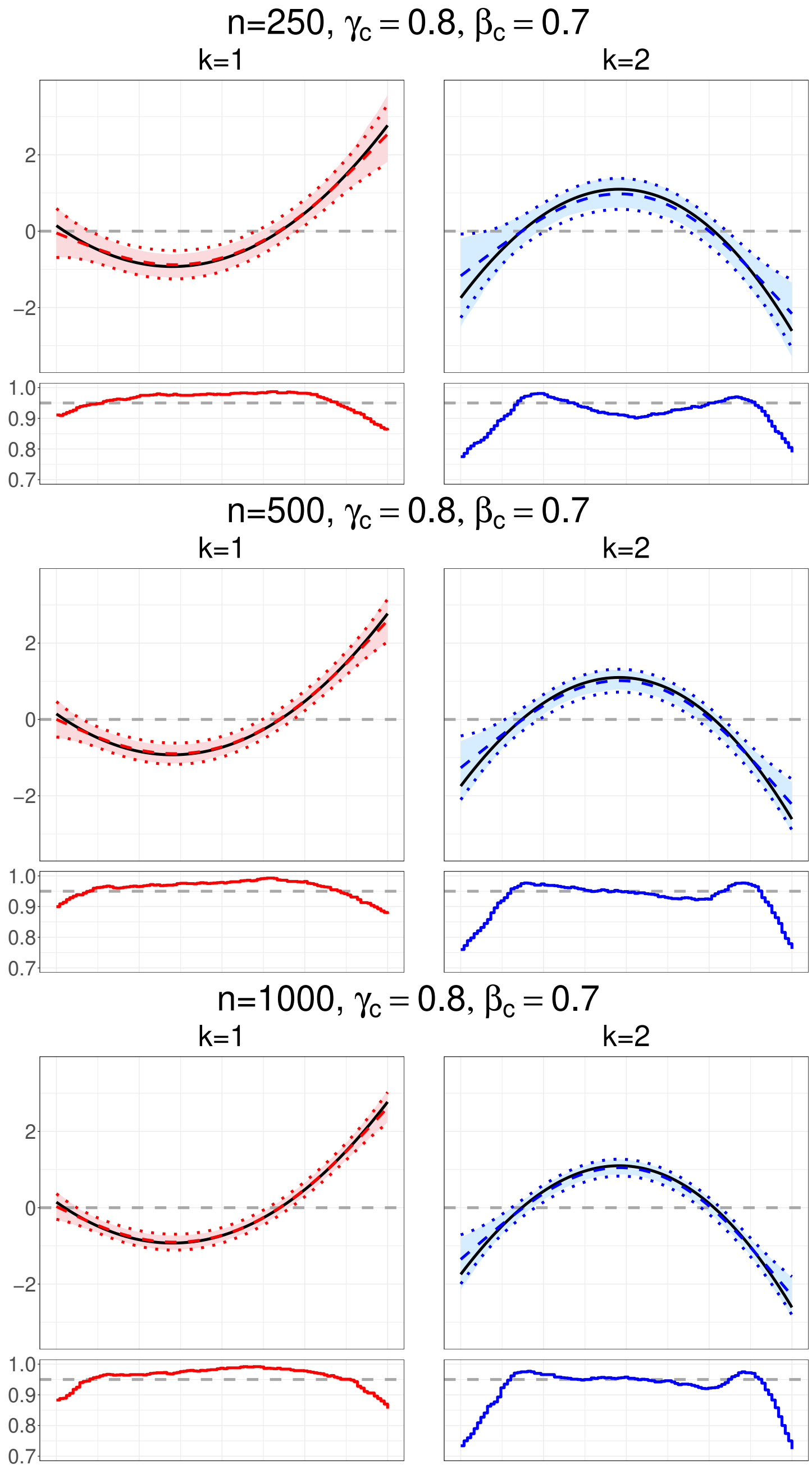

Figure S1 Simulation results for $\mathcal{X}_{[1]}$. For each simulation setting and each risk k, the top panel shows $\eta^*_{k,\{1\}}$ (solid line), average of $\hat{\eta}_{k,\{1\}}$ (dashed line), average of the 95% Bayesian confidence interval for $\eta^*_{k,\{1\}}$ (dotted lines), and 2.5% and 97.5% empirical quantiles of $\hat{\eta}_{k,\{1\}}$ (shaded area). The bottom panel displays the empirical coverage rate of the 95% Bayesian confidence interval for $\eta^*_{k,\{1\}}$. Note that all averages and empirical quantiles are computed point-wise across all replicates.

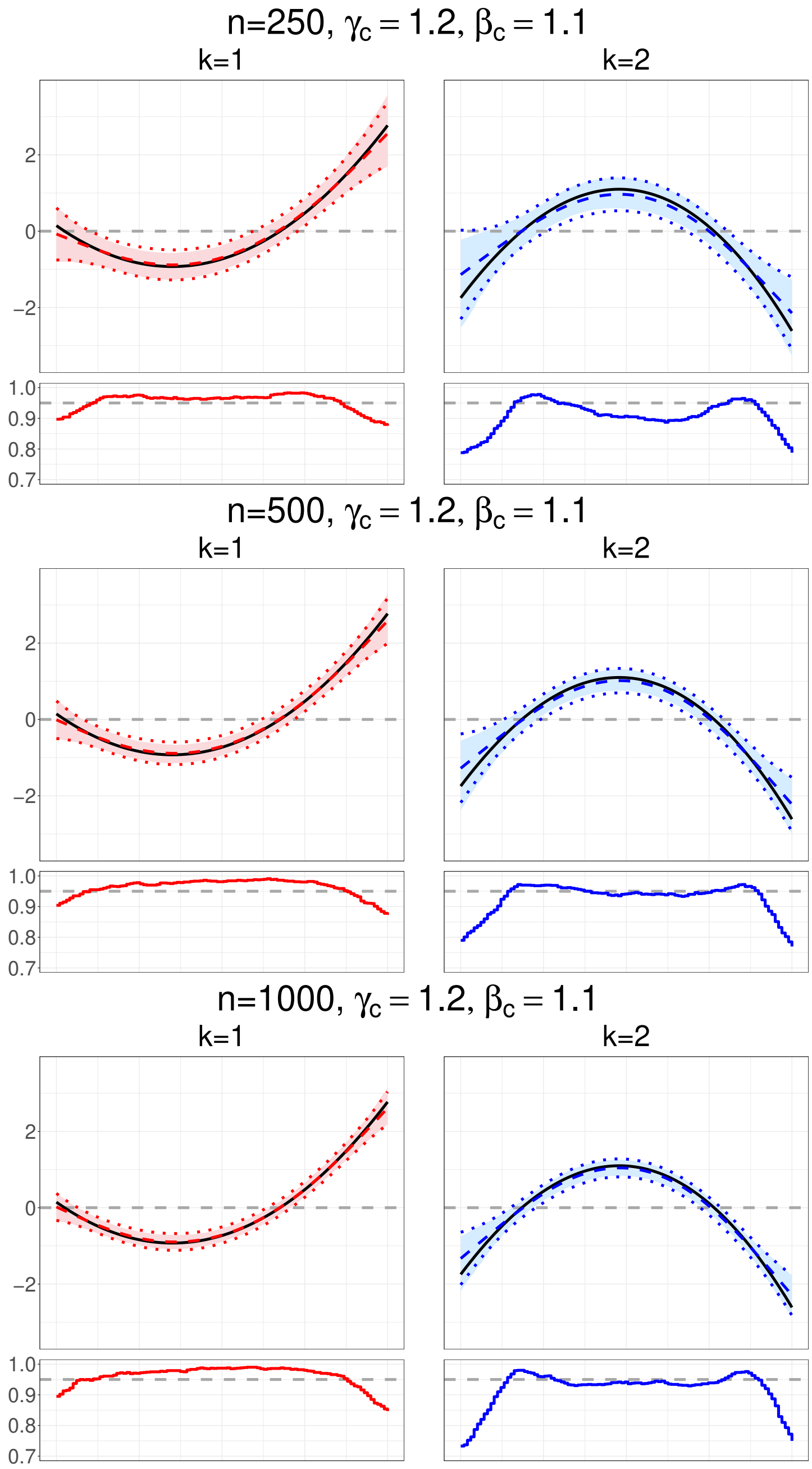

Figure S2 Simulation results for $\mathcal{X}_{[1]}$. For each simulation setting and each risk k, the top panel shows $\eta^*_{k,\{1\}}$ (solid line), average of $\hat{\eta}_{k,\{1\}}$ (dashed line), average of the 95% Bayesian confidence interval for $\eta^*_{k,\{1\}}$ (dotted lines), and 2.5% and 97.5% empirical quantiles of $\hat{\eta}_{k,\{1\}}$ (shaded area). The bottom panel displays the empirical coverage rate of the 95% Bayesian confidence interval for $\eta^*_{k,\{1\}}$. Note that all averages and empirical quantiles are computed point-wise across all replicates.

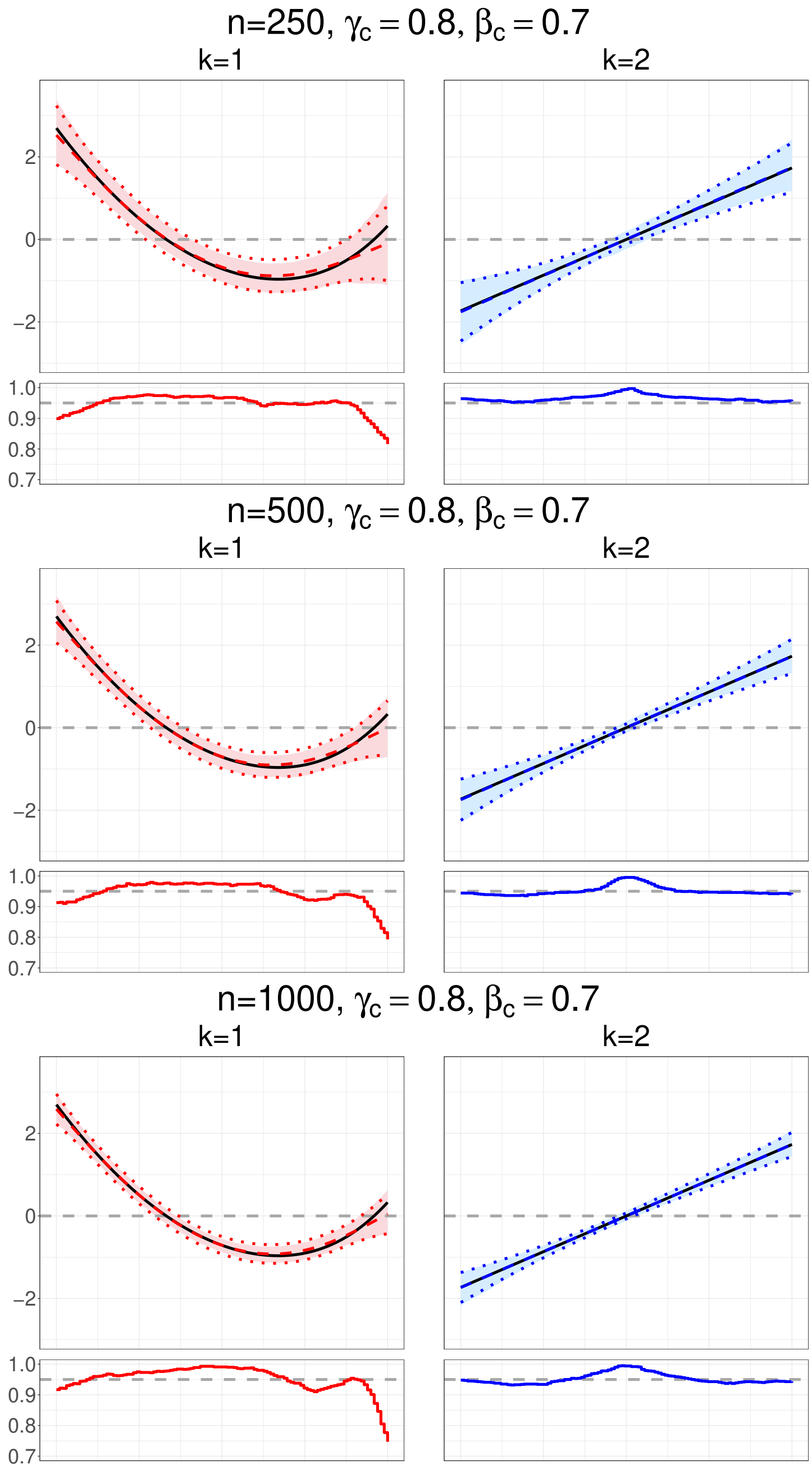

Figure S3 Simulation results for $\mathcal{X}_{[2]}$. For each simulation setting and each risk k, the top panel shows $\eta^*_{k,\{2\}}$ (solid line), average of $\hat{\eta}_{k,\{2\}}$ (dashed line), average of the 95% Bayesian confidence interval for $\eta^*_{k,\{2\}}$ (dotted lines), and 2.5% and 97.5% empirical quantiles of $\hat{\eta}_{k,\{2\}}$ (shaded area). The bottom panel displays the empirical coverage rate of the 95% Bayesian confidence interval for $\eta^*_{k,\{2\}}$. Note that all averages and empirical quantiles are computed point-wise across all replicates.

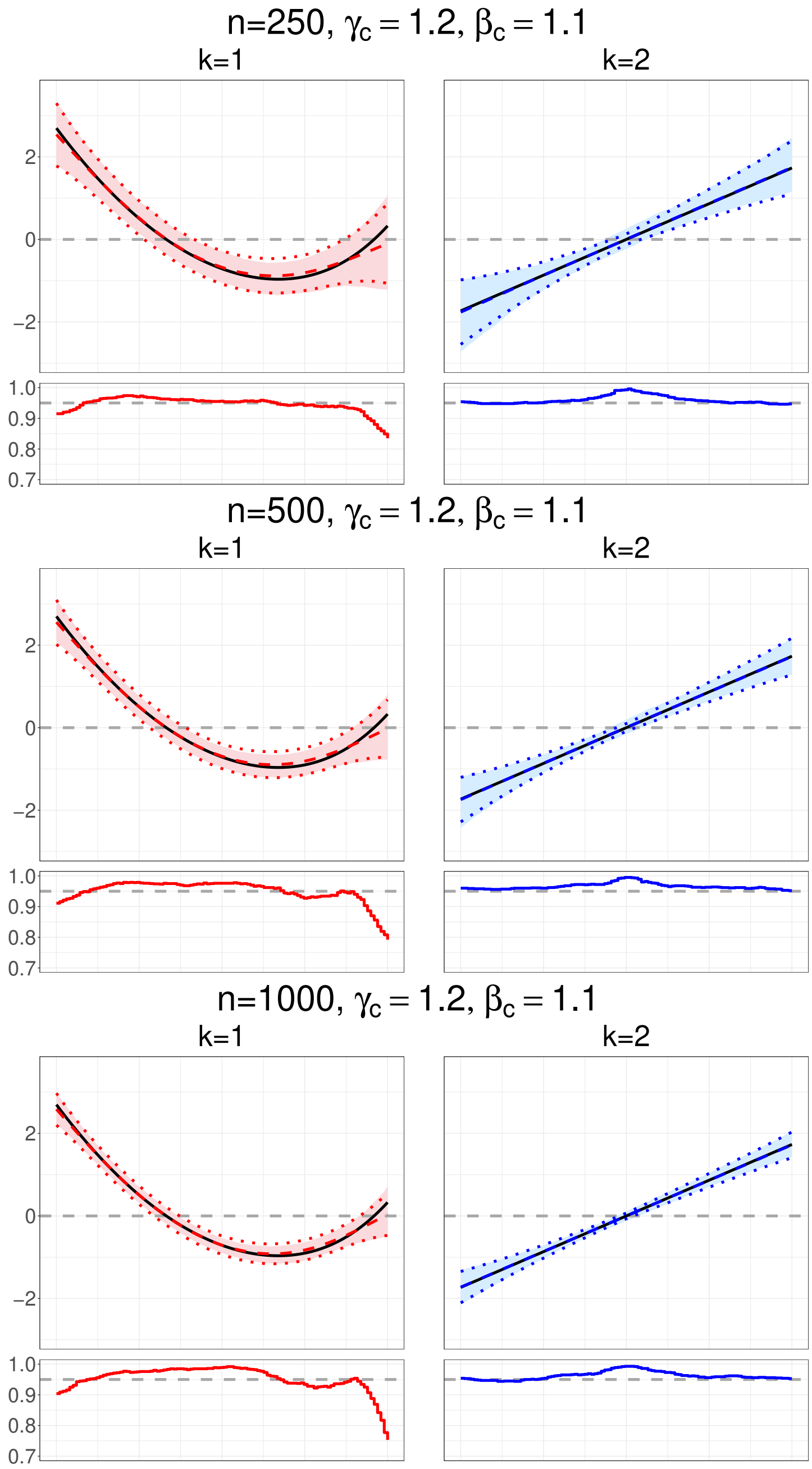

Figure S4 Simulation results for $\mathcal{X}_{[2]}$. For each simulation setting and each risk k, the top panel shows $\eta^*_{k,\{2\}}$ (solid line), average of $\hat{\eta}_{k,\{2\}}$ (dashed line), average of the 95% Bayesian confidence interval for $\eta^*_{k,\{2\}}$ (dotted lines), and 2.5% and 97.5% empirical quantiles of $\hat{\eta}_{k,\{2\}}$ (shaded area). The bottom panel displays the empirical coverage rate of the 95% Bayesian confidence interval for $\eta^*_{k,\{2\}}$. Note that all averages and empirical quantiles are computed point-wise across all replicates.

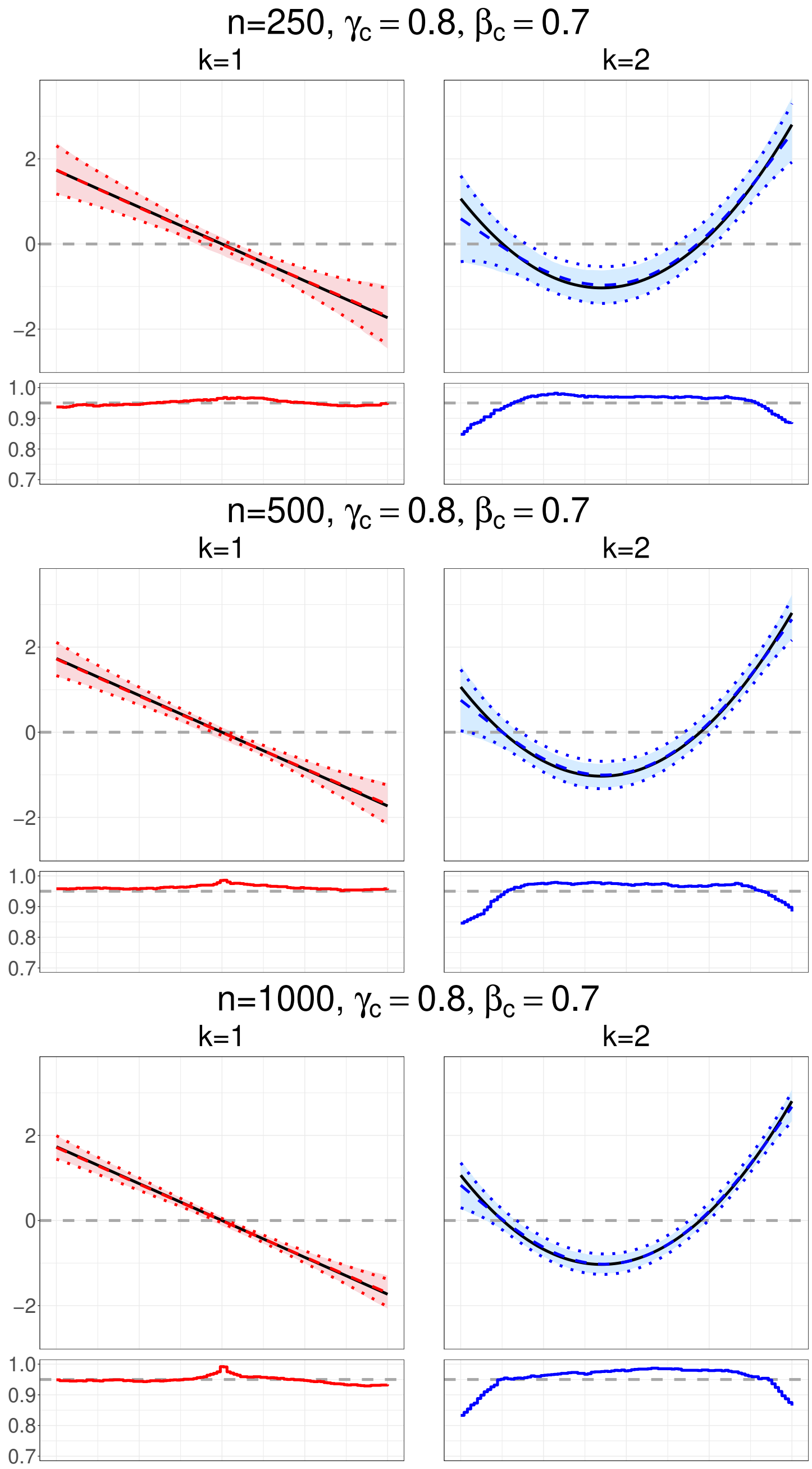

Figure S5 Simulation results for $\mathcal{X}_{[3]}$. For each simulation setting and each risk factor, the top panel shows $\eta^*_{k,\{3\}}$ (solid line), average of $\hat{\eta}_{k,\{3\}}$ (dashed line), average of the 95% Bayesian confidence interval for $\eta^*_{k,\{3\}}$ (dotted lines), and 2.5% and 97.5% empirical quantiles of $\hat{\eta}_{k,\{3\}}$ (shaded area). The bottom panel displays the empirical coverage rate of the 95% Bayesian confidence interval for $\eta^*_{k,\{3\}}$. Note that all averages and empirical quantiles are computed point-wise across all replicates.

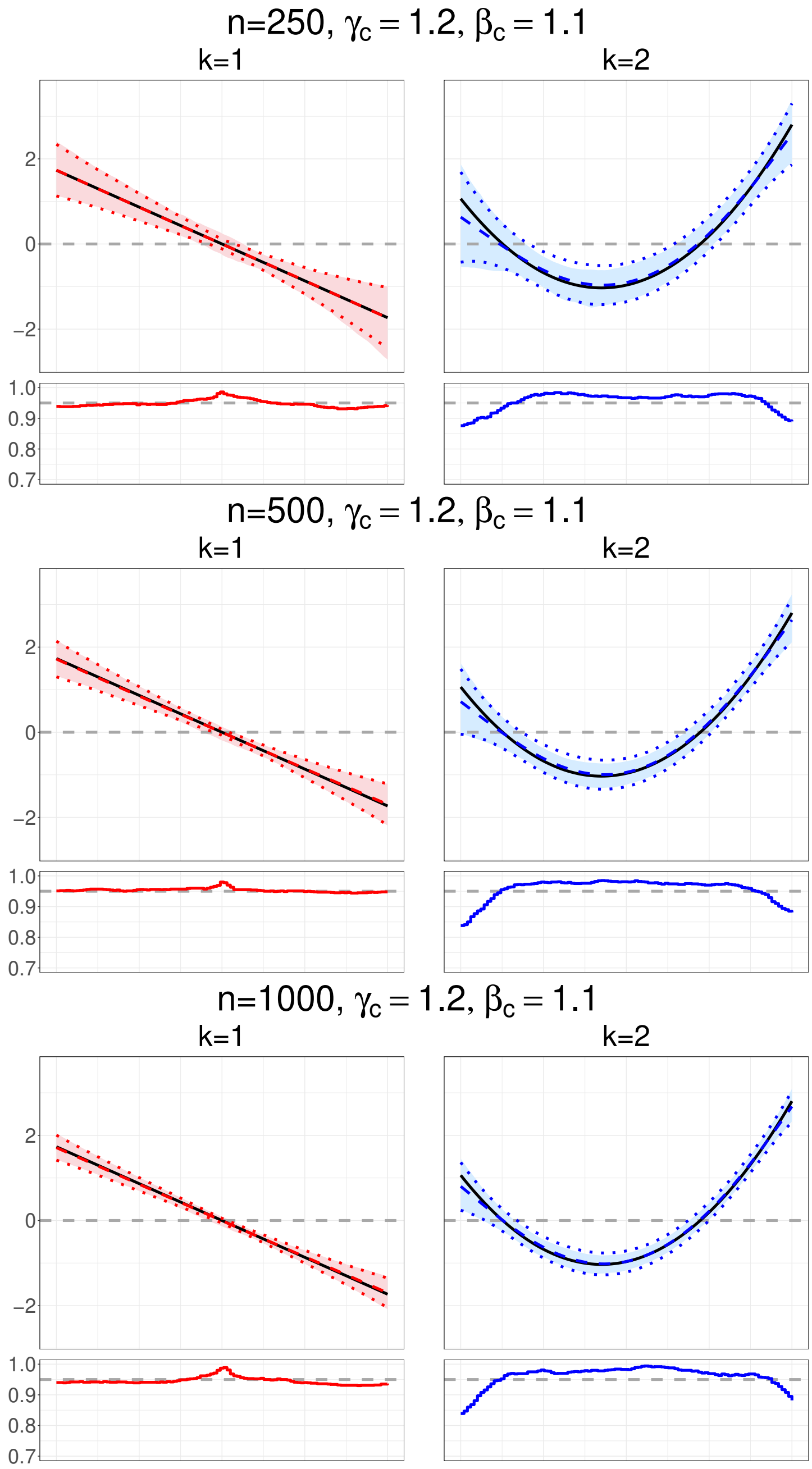

Figure S6 Simulation results for $\mathcal{X}_{[3]}$. For each simulation setting and each risk factor, the top panel shows $\eta^*_{k,\{3\}}$ (solid line), average of $\hat{\eta}_{k,\{3\}}$ (dashed line), average of the 95% Bayesian confidence interval for $\eta^*_{k,\{3\}}$ (dotted lines), and 2.5% and 97.5% empirical quantiles of $\hat{\eta}_{k,\{3\}}$ (shaded area). The bottom panel displays the empirical coverage rate of the 95% Bayesian confidence interval for $\eta^*_{k,\{3\}}$. Note that all averages and empirical quantiles are computed point-wise across all replicates.

Table S1 Simulation results for tensor-product structure 1. The first subtable reports the simulated data summaries by settings, and the second subtable reports the $\mathcal{L}_2$ losses by simulation settings. Each cell shows the mean (standard deviation) over 1,000 replicates.

n	h_c		Data Summary		
	γ_c	β_c	$\sum_{i=1}^{n} \mathbb{1}(G_i = 0)$	$\sum_{i=1}^{n} \mathbb{1}(G_i = 1)$	$\sum_{i=1}^{n} \mathbb{1}(G_i = 2)$
250	0.8	0.7	144.24(7.91)	53.55(6.46)	52.21(6.51)
	1.2	1.1	113.10(7.72)	69.63(7.03)	67.27(6.93)
	1.6	1.5	92.02(7.58)	81.03(7.50)	76.95(7.31)
500	0.8	0.7	287.85(11.48)	106.92(9.16)	105.23(9.43)
	1.2	1.1	225.59(11.47)	138.94(9.96)	135.47(10.09)
	1.6	1.5	184.29(11.18)	161.81(10.67)	153.90(10.50)
1000	0.8	0.7	576.89(15.57)	213.68(13.08)	209.44(12.70)
	1.2	1.1	450.55(15.66)	279.54(14.43)	269.92(13.92)
	1.6	1.5	368.30(15.35)	324.53(15.00)	307.17(15.24)

n	h_c		$\|\hat{\eta}_{k,S} - \eta^*_{k,S}\|_{\mathcal{L}_2}$					
			$S = \{1\}$		$S = \{2\}$		$S = \{1, 2\}$	
	γ_c	β_c	$k = 1$	$k = 2$	$k = 1$	$k = 2$	$k = 1$	$k = 2$
250	0.8	0.7	0.34(0.17)	0.50(0.45)	0.58(0.21)	0.65(0.45)	0.66(0.22)	0.73(0.39)
	1.2	1.1	0.32(0.18)	0.42(0.37)	0.53(0.21)	0.56(0.38)	0.59(0.21)	0.65(0.35)
	1.6	1.5	0.29(0.13)	0.33(0.32)	0.49(0.15)	0.46(0.31)	0.54(0.15)	0.55(0.30)
500	0.8	0.7	0.26(0.12)	0.29(0.29)	0.47(0.14)	0.42(0.28)	0.51(0.14)	0.49(0.28)
	1.2	1.1	0.22(0.10)	0.23(0.22)	0.44(0.12)	0.34(0.20)	0.46(0.11)	0.40(0.23)
	1.6	1.5	0.21(0.09)	0.19(0.17)	0.42(0.10)	0.30(0.16)	0.44(0.10)	0.36(0.19)
1000	0.8	0.7	0.19(0.09)	0.16(0.16)	0.40(0.09)	0.28(0.13)	0.42(0.10)	0.32(0.17)
	1.2	1.1	0.17(0.07)	0.13(0.10)	0.38(0.08)	0.23(0.08)	0.38(0.08)	0.27(0.10)
	1.6	1.5	0.16(0.07)	0.12(0.08)	0.37(0.07)	0.22(0.06)	0.37(0.07)	0.24(0.08)

Table S2 Simulation results for tensor-product structure 1. The first and second subtables report the empirical pointwise coverage probabilities and interval lengths of the Bayesian confidence intervals, respectively. The reported values are averaged over equally spaced grid points in the domain. Each cell shows the mean (standard deviation) over 1,000 replicates.

n	h_c		Empirical Coverage Probability of the Bayesian Confidence Interval					
	γ_c	β_c	$S=\{1\}$		$S=\{2\}$		$S=\{1,2\}$	
			$k=1$	$k=2$	$k=1$	$k=2$	$k=1$	$k=2$
250	0.8	0.7	0.84(0.22)	0.77(0.35)	0.77(0.25)	0.74(0.31)	0.82(0.18)	0.63(0.31)
	1.2	1.1	0.83(0.22)	0.78(0.34)	0.77(0.23)	0.76(0.30)	0.83(0.17)	0.67(0.30)
	1.6	1.5	0.86(0.20)	0.83(0.32)	0.79(0.21)	0.82(0.25)	0.86(0.14)	0.73(0.28)
500	0.8	0.7	0.86(0.21)	0.83(0.31)	0.77(0.22)	0.82(0.24)	0.85(0.15)	0.76(0.27)
	1.2	1.1	0.88(0.18)	0.87(0.28)	0.76(0.20)	0.85(0.20)	0.87(0.13)	0.82(0.23)
	1.6	1.5	0.89(0.17)	0.89(0.26)	0.78(0.17)	0.87(0.18)	0.87(0.12)	0.85(0.19)
1000	0.8	0.7	0.90(0.16)	0.90(0.25)	0.78(0.16)	0.86(0.17)	0.87(0.11)	0.87(0.17)
	1.2	1.1	0.90(0.17)	0.89(0.26)	0.76(0.15)	0.85(0.15)	0.87(0.10)	0.89(0.12)
	1.6	1.5	0.90(0.16)	0.90(0.25)	0.74(0.14)	0.83(0.14)	0.86(0.10)	0.90(0.09)

n	h_c		Empirical Length of the Bayesian Confidence Interval					
	γ_c	β_c	$S=\{1\}$		$S=\{2\}$		$S=\{1,2\}$	
			$k=1$	$k=2$	$k=1$	$k=2$	$k=1$	$k=2$
250	0.8	0.7	1.09(0.24)	1.16(0.37)	1.36(0.38)	1.31(0.27)	1.55(0.41)	1.18(0.35)
	1.2	1.1	0.98(0.20)	1.00(0.29)	1.23(0.32)	1.17(0.22)	1.39(0.30)	1.08(0.28)
	1.6	1.5	0.93(0.16)	0.88(0.26)	1.16(0.22)	1.10(0.18)	1.35(0.25)	1.05(0.25)
500	0.8	0.7	0.83(0.12)	0.78(0.22)	1.05(0.19)	0.98(0.15)	1.21(0.20)	0.95(0.20)
	1.2	1.1	0.75(0.10)	0.67(0.16)	0.94(0.16)	0.88(0.13)	1.09(0.17)	0.89(0.16)
	1.6	1.5	0.71(0.08)	0.60(0.15)	0.88(0.12)	0.82(0.11)	1.03(0.14)	0.84(0.12)
1000	0.8	0.7	0.64(0.07)	0.54(0.14)	0.81(0.11)	0.74(0.09)	0.93(0.12)	0.75(0.10)
	1.2	1.1	0.57(0.05)	0.46(0.10)	0.72(0.09)	0.64(0.07)	0.84(0.10)	0.67(0.08)
	1.6	1.5	0.54(0.05)	0.43(0.10)	0.67(0.08)	0.60(0.07)	0.79(0.08)	0.63(0.07)

Table S3 Simulation results for tensor-product structure 2. The first subtable reports the simulated data summaries by settings, and the second subtable reports the $\mathcal{L}_2$ losses by simulation settings. Each cell shows the mean (standard deviation) over 1,000 replicates.

n	h_c		Data Summary		
	γ_c	β_c	$\sum_{i=1}^{n} \mathbb{1}(G_i = 0)$	$\sum_{i=1}^{n} \mathbb{1}(G_i = 1)$	$\sum_{i=1}^{n} \mathbb{1}(G_i = 2)$
250	0.8	0.7	144.02(7.79)	51.35(6.37)	54.63(6.48)
	1.2	1.1	119.57(8.02)	62.33(7.00)	68.10(7.12)
	1.6	1.5	103.78(7.96)	69.50(7.05)	76.73(7.56)
500	0.8	0.7	288.21(10.98)	102.50(9.32)	109.30(9.37)
	1.2	1.1	237.92(11.05)	125.20(10.02)	136.88(9.79)
	1.6	1.5	207.22(11.17)	138.91(10.08)	153.86(10.19)
1000	0.8	0.7	577.06(16.47)	204.59(13.56)	218.35(12.91)
	1.2	1.1	477.69(15.47)	248.69(13.51)	273.62(13.70)
	1.6	1.5	416.20(15.31)	276.07(13.98)	307.73(14.34)

n	h_c		$\lVert\hat{\eta}_{k,S} - \eta^*_{k,S}\rVert_{\mathcal{L}_2}$					
	γ_c	β_c	$S = \{1\}$		$S = \{2\}$		$S = \{1, 2\}$	
			$k = 1$	$k = 2$	$k = 1$	$k = 2$	$k = 1$	$k = 2$
250	0.8	0.7	0.57(0.69)	0.41(1.46)	0.46(1.11)	0.40(2.39)	0.58(0.73)	0.40(1.46)
	1.2	1.1	0.48(0.45)	0.29(0.23)	0.36(0.56)	0.26(0.26)	0.51(0.56)	0.28(0.23)
	1.6	1.5	0.43(0.23)	0.25(0.21)	0.29(0.27)	0.22(0.20)	0.44(0.25)	0.26(0.20)
500	0.8	0.7	0.38(0.20)	0.22(0.17)	0.25(0.23)	0.19(0.18)	0.38(0.22)	0.21(0.17)
	1.2	1.1	0.34(0.16)	0.19(0.15)	0.21(0.18)	0.16(0.13)	0.34(0.17)	0.18(0.14)
	1.6	1.5	0.32(0.15)	0.17(0.13)	0.19(0.16)	0.15(0.13)	0.31(0.16)	0.17(0.13)
1000	0.8	0.7	0.28(0.13)	0.15(0.11)	0.17(0.14)	0.12(0.10)	0.29(0.15)	0.15(0.11)
	1.2	1.1	0.26(0.13)	0.13(0.09)	0.16(0.13)	0.11(0.09)	0.26(0.13)	0.12(0.10)
	1.6	1.5	0.24(0.12)	0.12(0.09)	0.15(0.13)	0.10(0.08)	0.25(0.12)	0.11(0.09)

Table S4 Simulation results for tensor-product structure 2. The first and second subtables report the empirical pointwise coverage probabilities and interval lengths of the Bayesian confidence intervals, respectively. The reported values are averaged over equally spaced grid points in the domain. Each cell shows the mean (standard deviation) over 1,000 replicates.

n	h_c		Empirical Coverage Probability of the Bayesian Confidence Interval					
	γ_c	β_c	$S=\{1\}$		$S=\{2\}$		$S=\{1,2\}$	
			$k=1$	$k=2$	$k=1$	$k=2$	$k=1$	$k=2$
250	0.8	0.7	0.84(0.20)	0.95(0.18)	0.94(0.24)	0.96(0.19)	0.83(0.22)	0.94(0.21)
	1.2	1.1	0.84(0.21)	0.95(0.18)	0.92(0.27)	0.96(0.19)	0.81(0.23)	0.95(0.19)
	1.6	1.5	0.83(0.21)	0.94(0.20)	0.92(0.27)	0.96(0.19)	0.82(0.22)	0.94(0.20)
500	0.8	0.7	0.84(0.21)	0.95(0.20)	0.91(0.28)	0.96(0.19)	0.83(0.22)	0.95(0.20)
	1.2	1.1	0.85(0.20)	0.95(0.20)	0.92(0.27)	0.96(0.19)	0.83(0.21)	0.94(0.20)
	1.6	1.5	0.85(0.20)	0.95(0.20)	0.92(0.27)	0.95(0.22)	0.85(0.20)	0.94(0.20)
1000	0.8	0.7	0.87(0.19)	0.94(0.21)	0.93(0.26)	0.96(0.20)	0.83(0.22)	0.94(0.22)
	1.2	1.1	0.85(0.21)	0.95(0.20)	0.89(0.31)	0.95(0.22)	0.83(0.22)	0.94(0.21)
	1.6	1.5	0.86(0.21)	0.94(0.21)	0.89(0.31)	0.96(0.19)	0.84(0.22)	0.95(0.19)

n	h_c		Empirical Length of the Bayesian Confidence Interval					
	γ_c	β_c	$S=\{1\}$		$S=\{2\}$		$S=\{1,2\}$	
			$k=1$	$k=2$	$k=1$	$k=2$	$k=1$	$k=2$
250	0.8	0.7	1.95(4.73)	1.56(3.93)	2.13(9.28)	1.75(7.67)	1.89(4.75)	1.52(3.94)
	1.2	1.1	1.54(1.38)	1.15(0.38)	1.47(1.73)	1.17(0.54)	1.50(1.40)	1.12(0.39)
	1.6	1.5	1.34(0.37)	1.03(0.26)	1.21(0.43)	1.03(0.32)	1.28(0.42)	1.00(0.27)
500	0.8	0.7	1.18(0.25)	0.88(0.20)	1.04(0.31)	0.88(0.24)	1.13(0.30)	0.85(0.21)
	1.2	1.1	1.05(0.22)	0.76(0.15)	0.89(0.24)	0.75(0.15)	1.00(0.26)	0.73(0.15)
	1.6	1.5	0.97(0.17)	0.69(0.14)	0.82(0.20)	0.69(0.14)	0.93(0.22)	0.67(0.14)
1000	0.8	0.7	0.87(0.13)	0.60(0.10)	0.71(0.14)	0.59(0.10)	0.82(0.17)	0.58(0.11)
	1.2	1.1	0.78(0.11)	0.52(0.09)	0.62(0.12)	0.51(0.08)	0.74(0.14)	0.50(0.09)
	1.6	1.5	0.73(0.10)	0.48(0.08)	0.58(0.11)	0.47(0.06)	0.70(0.12)	0.46(0.08)

References

Du P, Ma S, Liang H (2010) Penalized variable selection procedure for Cox models with semiparametric relative risk. The Annals of Statistics 38(4):2092 – 2117

Fleming TR, Harrington DP (1991) Counting Processes and Survival Analysis. John Wiley & Sons, Inc, Hoboken, New Jersey

Gu C (2013) Smoothing Spline ANOVA Models, 2nd edn. Springer Series in Statistics, Springer, New York

O'Sullivan F (1993) Nonparametric Estimation in the Cox Model. The Annals of Statistics 21(1):124 – 145
